# Immunoinformatic Approach to Contrive a Next Generation Multi-Epitope Vaccine Against *Achromobacter xylosoxidans* Infections

**DOI:** 10.3389/fmed.2022.902611

**Published:** 2022-07-11

**Authors:** Kashaf Khalid, Umar Saeed, Mohammad Aljuaid, Mohammad Ishtiaq Ali, Awais Anjum, Yasir Waheed

**Affiliations:** ^1^Multidisciplinary Laboratory, Foundation University Islamabad, Islamabad, Pakistan; ^2^Biological Production Division, National Institute of Health, Islamabad, Pakistan; ^3^Department of Health Administration, College of Business Administration, King Saud University, Riyadh, Saudi Arabia; ^4^Department of Microbiology, Quaid-i-Azam University, Islamabad, Pakistan; ^5^PerkinElmer Inc., Newport, United Kingdom; ^6^Clinical and Biomedical Research Center, Foundation University Islamabad, Islamabad, Pakistan; ^7^Office of Research, Innovation and Commercialization (ORIC), Shaheed Zulfiqar Ali Bhutto Medical University, Islamabad, Pakistan

**Keywords:** *Achromobacter xylosoxidans*, gram-negative bacteria, Immunoinformatic approaches, multi-epitope vaccine, docking

## Abstract

Achromobacter xylosoxidans, previously identified as *Alcaligenes xylosoxidans*, is a rod-shaped, flagellated, non-fermenting Gram-negative bacterium that has the ability to cause diverse infections in humans. As a part of its intrinsic resistance to different antibiotics, *Achromobacter* spp. is also increasingly becoming resistant to Carbapenems. Lack of knowledge regarding the pathogen’s clinical features has led to limited efforts to develop countermeasures against infection. The current study utilized an immunoinformatic method to map antigenic epitopes (Helper T cells, B-cell and Cytotoxic-T cells) to design a vaccine construct. We found that 20 different epitopes contribute significantly to immune response instigation that was further supported by physicochemical analysis and experimental viability. The safety profile of our vaccine was tested for antigenicity, allergenicity, and toxicity against all the identified epitopes before they were used as vaccine candidates. The disulfide engineering was carried out in an area of high mobility to increase the stability of vaccine proteins. In order to determine if the constructed vaccine is compatible with toll-like receptor, the binding affinity of vaccine was investigated *via* molecular docking approach. With the *in silico* expression in host cells and subsequent immune simulations, we were able to detect the induction of both arms of the immune response, i.e., humoral response and cytokine induced response. To demonstrate its safety and efficacy, further experimental research is necessary.

## Introduction

*Achromobacter xylosoxidans* is a non-fermenter, Gram-negative bacterium that belongs to the *Alcaligenaceae* family. Since the past few years, it has gained notoriety as a pathogen responsible for nosocomially acquired infections among immunocompromised as well as immunocompetent people (1, 2). Researchers Ybuuchi and Ohyama first isolated it in 1971 after observing chronic otitis media patients’ ear discharge (3). The opportunistic pathogen is associated with a plethora of lethal infections such as septicemia (4), pneumonia (2), urinary tract infection (5), meningitis, peritonitis and other infections (6–8). Recently, it has been discovered that the bacterium plays a prominent role in causing cystic fibrosis worldwide (9).

*A. xylosoxidans* is an opportunistic motile bacterium with peritrichous flagella and often confused with *Pseudomonas* (10). The pathogen is extensively found in water, soil and hospital environments (11) and has proven to be extensively resistant to antibiotics as they harbor excellently characterized resistance mechanisms and at least 50 intrinsic resistance genes (12). In addition to intrinsic resistance, acquired resistance is widely being reported across several regions of the world such as the United States (13), Europe (14), China (15) along with many other regions. In spite of that, the optimal treatment for *Achromobacter spp.* is still unknown. In the face of increasingly prevalent pathogenic bacterium, limited attention is being put toward its treatment, posing an insurmountable clinical challenge and a state of worldwide emergency.

In the current post-genomic era, emphasis has majorly been shifted from antibiotic-based approaches to non-antibiotic-based approaches for the treatment of pathogen induced infections. Particularly, multi epitope vaccine constructs (MEVC) have been shown to be a credible alternative to the obsolete antibiotic-based treatment regimens (16). A multitude of attempts have been made to design potent, safe, and immunostimulant vaccines that are superior to traditional vaccines using AI and immunoinformatic tools (17). In spite of the plethora of available information and the computational resources, no attempt has been made yet to develop a vaccine against this looming threat. Therefore, we made an effort to build a rational multi-epitope next generation therapeutic vaccine against the MDR pathogen by using several *in silico* approaches.

To accomplish this purpose, we used the reverse vaccinology (RV) technique to perform genome mining to filter the proteins that are most immunogenic, as well as non-allergenic and safe to use. A successful working example of the RV approach is the vaccine design against Meningococcus B pathogen where genomic based approaches were used to predict ninety novel antigens (18). This invention led to initiation of plethora of studies on development of vaccines against several dominant and emerging pathogens such as *Klebsiella pneumoniae* (19), *Acinetobacter baumannii* (20), *Helicobacter pylori* (21), *Yersinia pestis* (22) and many others.

## Materials and Methods

### Retrieval of Complete Proteome

**FIGURE 1:**
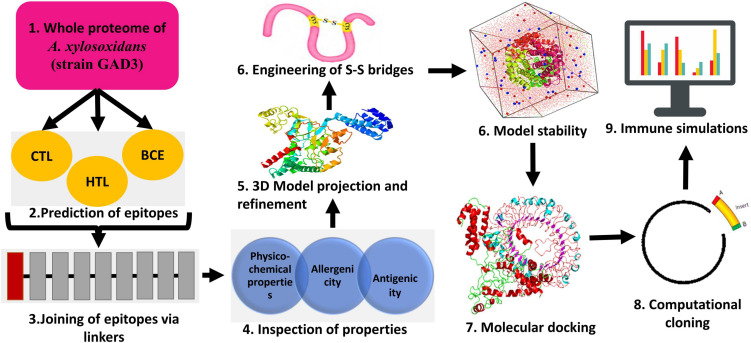
A general workflow employed during the vaccinomics study throughout.

As a first step, GenBank Database was employed to retrieve the complete proteome of *A. xylosoxidans* comprising 5,729 proteins (strain GAD3: Accession no. GCF_003031105.1). Figure 1 presents the study’s step-by-step process.

### Determination of CTL Epitopes

Using NetCTL 1.2, we identified CTL epitopes in the polyprotein sequence (23). Three elements are combined in this prediction: first, the binding peptide for MHC-I is predicted, then the C-terminal proteasome cleavage is predicted, and it is finally predicted that the Transporter Associated with Antigen Processing (TAP) program is performed. Artificial neural networks were used to estimate the first two parameters, whereas weight matrices were used to determine the TAP transporter’s efficiency. In the prediction of CTL epitopes, a cut-off value of 0.75 was permitted. We used the PSORTb to determine the localization of the proteins from which all the epitopes were derived (24).

### Determination of HTL Epitopes

With the help of IEDB, 15-mer amino acid length HTL epitopes with excellent affinity were predicted for a reference panel of seven alleles. Our predicted peptides were sorted based on their IC_50_ scores; good binders had an IC_50_ of < 50 nM, intermediate binders had < 500 nM, and low affinity binders had < 5,000 nM. A lower percentile rank represents a higher binding affinity, as revealed by the inverse correlation between percentile ranking and binding affinity (25).

### Study of Population Coverage

Analyses of the world population coverage was conducted with the IEDB server (25). We used the selected epitopes and compared them to the respective allele sets in a wide range of global populations. In the coverage analyses, it was primarily measured whether the selected epitopes were able to cover large populations. Analysis was done on the United States (13), European countries (14), and China (15), which are being hit hardest by the outbreak of *Achromobacter.*

### Determination of Continuous B-Cell Epitope Regions

B-cells produce antibodies that serve as a long-term source of immunity. Therefore, to stimulate the protective immune response of the host, the continuous B-cell epitopes were anticipated in the bacterial proteome using an online webserver, BCPred (26). To predict epitopes, BCPred employs a support vector machine (SVM) algorithm and a successive kernel approach. A threshold of 0.8 was established initially as a cut-off point to eliminate the top predicted B-cell epitopes.

### Contriving the Immunogenic Construct

Having prioritized carefully determined epitopes from previous steps, we were able to design a multi epitope vaccine construct. Linkers AAY, GPGPG and KK were, respectively, used between CTL, HTL, and B cell epitopes to accomplish this (27). Linking sequences are essential for extending the conformation of proteins (flexibility), allowing foldability, and separating functional domains, which ultimately makes the overall structure of protein more stable and therefore facilitates the protein expression (28). The complex was adjuvanated by connecting an immune potentiating protein (50S ribosomal protein L7/L12, Accession: P9WHE3) at the amine terminus so as to trigger an adequate amount of immune response against the pathogen (29).

### Determination of Allergenicity and Antigenic Potential of the Proposed Construct

The vaccine sequence’s allergenicity was assessed using AlgPred, an established allergy prediction server (30). According to the server, allergenic sequences are predicted to an accuracy of 85%. Identifying allergens can be done based on their scores (> 0.4). The vaccine construct must have antigenicity to trigger the proper immunogenic response. To further confirm the non-allergenicity of the proposed vaccine, AllerTop v2 was employed (31). We employed the VaxiJen server to determine the construct‘s antigenicity, retaining the default threshold value of 0.45 (32). By converting protein sequences into a vector of key amino acid properties using automatic cross correlation, VaxiJen predicts antigens without alignment. Multiple biological functions depend on protein solubility and stability. Therefore, vaccine‘s solubility was determined using two web servers SOLpro (33) and Protein-Sol (34). In SOLpro, solubility is predicted using the probability scores. Thus, soluble proteins receive scores of ≥ 0.5 and insoluble proteins receive scores of < 0.5. In contrast, Protein-Sol predicts the vaccine construct’s solubility based on solubility data.

### Physicochemical Profiling of the Proposed Construct

On the ProtParam website, the vaccine’s physicochemical properties were revealed which involve theoretical pI, molecular weight, construct‘s half-life, instability index, and GRAVY (35). 2-D assembly of the designed construct was calculated using PSIPRED (36).

### 3D Modeling, Refinement and Quality-Check

For 3D model prediction, I-TASSER was harnessed which is a web tool for computer-assisted function and structure determination of proteins using sequence-to-structure-to-function analysis, with the PDB used to identify similar structure patterns (37). The initial 3D atomic models produced by I-TASSER originate from numerous threading alignments and reiterative simulations of structure beginning with a protein sequence. Models with TM-values greater than 0.5 generally express precise topology, while those with TM-values lesser than 0.17 show random similarity. A protein’s length has no bearing on the cutoff value (38). The community-wide CASP analysis of vaccine’s 3D structure evaluation using I-TASSER projection, refinement, and validation has consistently listed it as the best server for protein 3D structure determination (39).

The projected 3D structure was subjected to further refinement to improve local and global structure quality using ModRefiner (40) and Galaxy Refine (41) web servers. CASP10 refinement was (42) used to reconstruct and repack the side chain of the protein, which was then relaxed through MD simulation. Thereafter, a model validation step was performed to pinpoint probable limitations in the refined structure (39). Initially, ProSA-web server was employed to gauge the overall quality in terms of Z-value (43). The Z-scores are erroneous if they do not fall within the range of properties for pre-determined protein structures. In order to investigate non-bonding atoms–atom interactions, the ERRAT web server was used (44). By displaying the percentages of residues around favorable and unfavorable regions on the Ramachandran plot, the overall quality of the modeled structure was described using RAMPAGE’s web server (45). The loops were optimized using ModLoop, a server that analyzes the proteins’ angles so that the loops have proper φ and ψ angles (46).

### Stabilization of Vaccine Through Loop Remodeling and Disulfide Engineering

Providing more stability to the refined protein model was required before proceeding with the next step. Loop re-modeling in the 3D structure of vaccine was carried out by using Galaxy Loop server (47). Furthermore, disulfide bonds were introduced into the protein model *via* an online server-Disulfide by Design 2.0 (48). Disulfide bond is a covalent interaction that mimics stable molecular interactions and demonstrates the precise geometric conformation of proteins and therefore contributes to their stability. Using disulfide engineering, one can introduce disulfide bonds into a protein structure. Therefore, the initial model of the refined protein was uploaded and used for the residue pair search necessary to engineer disulfides. Total 4 residue pairs were selected to mutate them with cysteine residue using create mutate function of the Disulfide by Design 2.0 server.

### Non-linear B-Cell Epitope Determination

Nearly 90% of the B-cell epitopes, owing to their spatial arrangements, are discontinuous and found close together (49). Due to conformational B-cell epitopes playing a significant part in eliciting an antibody-mediated immune response, it is imperative to determine their presence in the developed vaccine construct (42). This was accomplished by using the ElliPro server (50), which determines epitopes on the basis of PI values, where PI equal to 0.9 includes 90% of residues in an ellipsoid, and excludes 10% from it. The epitopes with the top PI values were selected.

### Folding Stability Analysis *via* MD Simulation Technique

For proper regulation of cellular protein networks, which perform various biological functions, such as cell-cell communication and activation of immune response and so on, appropriate folding of proteins is necessary (27). This study utilized all-atom molecular dynamics simulations approach to determine how the vaccine constructs fold and function. Using a freeware-GROMACS (version 5.0), we set up a simulation system that included water and ions, as well as the designed vaccine construct (51). Initially, pdb2gmx module of GROMACS was harnessed to generate a force-filed compliant topology of the designed construct. The system was then applied the force field with OPLS-AA, after which the editconf module was used to create a rhombic dodecahedron box that later on was solvated with water molecules. The solvated system after going through electro-neutralization stage, was checked for steric clashes and inappropriate geometry *via* the energy minimization (EM) technique. Following relaxation of the structure with EM, ions and solvents were equilibrated under NVT and NPT ensembles for 100 and 1,000 ps, respectively. The equilibrated system was then subjected to dynamic simulations for 50 ns. By examining the MD trajectories, we were able to compute data such as RMSD, RMSF and RG (52).

### Molecular Docking With Immune Receptors

It is imperative that the vaccine interact with the target immune cell receptors in order to cause a stable immunogenic response. Therefore, we conducted a molecular docking analysis to decipher the interaction among the designed construct as well as the human toll like receptor. Specifically, TLR4 was studied as it is actively involved in invoking the immune response against the Gram negative bacteria (53). To fetch the pdb format of TLR4 from the Protein Data Bank, ID: 4G8A was used. The docking process was driven using the ZDOCK server (54). Using rigid-body docking programs, the ZDOCK web server generates quick and accurate complexes. We used the option of selecting contacting/blocking residues and selected the active residues (I48, D50, F54, Y72, S73, F75, S76, S100) from the B chain of the TLR 4 (55). Furthermore, the complexes generated by ZDOCK were sent for refinement and post processing *via* the FireDock server (56). The FireDock server allows high throughput refinement of docked complexes *via* side chain optimization. As a final step, using PDBsum, interactions between the vaccine and the host TLRs were mapped (57). In order to measure the epitope binding efficiency, the crystallographic ligand KDO was used as a positive control.

### *In silico* Cloning Experiment

Optimizing codons in the host cell ensures maximum expression. We therefore optimized the sequence using JCat tool in accordance with our preferred expression organism, *Escherichia coli K12* (58). In addition to GC content, the result provides a codon adaptation index (CAI) score. The score reflects the level of favorability of protein expression. SnapGene software was used for *in silico* cloning using pET28a (+) vector backbone.

### Immune Simulations

Foreign particles and the immune system can be studied by simulating their interactions. For this purpose, C-ImmSim was harnessed (59). The reactivity of the host immune system to foreign particles is studied by an agent-based modeling approach. Researchers are exploring immune responses in realistic virtual environments due to the recent interest in computational learning and development. With the PSSM model, the server measures the immune system’s reaction to an antigen. Estimates are provided for antibodies, interferon, cytokines, and other immune substances produced by vaccination. During the entire simulation, 1400-time steps were run, which is approximately 15 months (each time step lasts 8 h).

## Results

### Data Retrieval

From the NCBI database, we downloaded the completely sequenced proteome of *A. xylosoxidans* and examined T cell epitopes and B cell epitopes so as to stimulate the respective arms of the immune system in order to design a potent multi-epitope vaccine against the pathogen.

### Proteome Mining for CTLs

Modern vaccines consist of a combination of epitopes, out of which CTLs are important in vaccine‘s efficient stimulation of the immune response. A thorough analysis of complete proteome of *A. xylosoxidans* revealed a total of 35 potential CTL epitopes as 9-mer peptides (Table 1). These peptides were filtered on the bases of good binding affinity. Ultimately, only eleven epitopes showed the greatest antigenic potential and combined score and were found to be localized in the outer membrane and periplasmic regions which made them perfect candidates to be incorporated into the final vaccine construct.

**TABLE 1:** Predicted 9-mer peptides as the potential cytotoxic-T cell epitopes.

Sr. no	Residue ID.	Peptide Sequence (9 mer)	MHC Binding Affinity	Rescale Binding Affinity	C-Terminal Cleavage Affinity	TAP score	combined Score	MHC-I Binding	Antigenicity Score	ToxinPred	AllerTop
1	79	PTDKTVDAL	0.1763	0.7484	0.8108	0.1380	0.8770	✓	Non-antigen	-	-
2	13	STAGTGHFY	0.7048	2.9925	0.8299	2.8060	3.2573	✓	0.99	Non-toxin	Allergen
3	41	PVARKHVDY	0.1391	0.5907	0.9643	2.4610	0.8584	✓	Non-antigen	-	-
4	29	LSEQGFNVF	0.2172	0.9221	0.8489	2.3830	1.1686	✓	Non-antigen	-	-
5	99	GLEMGADDY	0.2783	1.1815	0.7952	0.7952	2.6680	✓	1.06	Non-toxin	Allergen
6	228	IQTVWGLGY	0.1721	0.7309	0.9704	2.8802	1.0206	✓	0.46	Non-toxin	Allergen
**7**	**05**	**MCLICGWVY (O)**	**0.1306**	**0.5543**	**0.5592**	**2.9540**	**0.7859**	**✓**	**1.04**	**Non-toxin**	**Non-allergen**
8	64	TTVQQAIDY	0.4884	2.0736	0.8348	2.9690	2.3473	✓	Non-antigen	-	-
**9**	**87**	**VKDLPGVRY (O)**	**0.1157**	**0.4912**	**0.9662**	**2.9920**	**0.7857**	**✓**	**1.18**	**Non-toxin**	**Non-allergen**
**10**	**133**	**PTTTVHGVF (O)**	**0.1261**	**0.5355**	**0.9066**	**2.1510**	**0.7790**	**✓**	**0.59**	**Non-toxin**	**Non-allergen**
**11**	**141**	**FLDVLGLGV (O)**	**0.2209**	**0.9381**	**0.8986**	**0.1100**	**1.0784**	**✓**	**0.88**	**Non-toxin**	**Non-allergen**
12	09	FSSDMAIDL	0.1743	0.77402	0.5425	0.8440	0.8638	✓	0.68	Non-toxin	Allergen
13	156	VSDASGSMV	0.4340	1.8425	0.1304	0.3950	1.8818	✓	0.86	Non-toxin	Allergen
**14**	**171**	**TTEVAVISL (P)**	**0.1609**	**0.6830**	**0.6335**	**0.7380**	**038149**	**✓**	**0.88**	**Non-toxin**	**Non-allergen**
15	176	VISLGGMVY	0.1991	0.8455	0.9705	3.2650	1.1543	✓	Non-antigen	-	-
16	261	LTDPLNQIV	0.5305	2.2523	0.7255	0.1750	2.3699	✓	Non-antigen	-	-
17	44	ILLSSMTGY	0.1260	0.5350	0.9524	3.1170	0.8337	✓	Non-antigen	-	-
18	58	QMTGVVHEY	0.3152	1.3382	0.9744	3.0700	1.6379	✓	Non-antigen	-	-
19	131	LTDAGKLEM	0.3861	1.6394	0.8950	0.2520	1.7862	✓	Non-antigen	-	-
20	192	RTDLDKLVL	0.2002	0.8500	0.9140	0.9520	1.0347	✓	Non-antigen	-	-
**21**	**268**	**CLKAENIYY (P)**	**0.2354**	**0.9993**	**0.8162**	**3.0420**	**1.2738**	**✓**	**1.23**	**Non-toxin**	**Non-allergen**
22	20	VSDGIAHVH	0.2379	1.0101	0.3047	0.4680	1.0324	✓	Non-antigen	-	-
**23**	**38**	**ITDRQGNAL (O)**	**0.2231**	**0.9472**	**0.9165**	**0.7900**	**1.1242**	**✓**	**0.83**	**Non-toxin**	**Non-allergen**
**24**	**73**	**TAGRVALEY (P)**	**0.2929**	**1.2435**	**0.9378**	**2.6260**	**1.5155**	**✓**	**0.81**	**Non-toxin**	**Non-allergen**
25	03	MSDPIADML	0.3963	1.6826	0.6579	0.9200	1.8273	✓	Non-antigen	-	-
**26**	**121**	**GVGGEVLCY (P)**	**0.1417**	**0.6015**	**0.9548**	**2.8620**	**0.8878**	**✓**	**1.34**	**Non-toxin**	**Non-allergen**
27	01	MSETQNTQV	0.3647	1.5484	0.9547	0.2560	1.7044	✓	1.42	Non-toxin	Allergen
28	47	HVDTGDYIV	0.2039	0.8658	0.6309	0.0410	0.9624	✓	Non-antigen	-	-
29	108	MLPKGPLGY	0.1866	0.7923	0.9546	2.9460	1.0828	✓	Non-antigen	-	-
30	181	GTDGHAHIV	0.3340	1.4182	0.9094	0.2290	1.5432	✓	1.74	Non-toxin	Allergen
**31**	**252**	**DAEEIKLRY (P)**	**0.3619**	**1.5366**	**0.9363**	**2.3720**	**1.7957**	**✓**	**2.44**	**Non-toxin**	**Non-allergen**
**32**	**100**	**GSCANGGGY (P)**	**0.3658**	**1.5533**	**0.3409**	**2.8310**	**1.7459**	**✓**	**2.48**	**Non-toxin**	**Non-allergen**
33	13	FTQQNPLFK	0.1771	0.7517	0.22313	0.4170	0.8058	✓	0.41	-	-
34	306	NAESVSSLF	0.1621	0.6883	0.5846	2.5340	0.9026	✓	Non-antigen	-	-
35	412	MTGSQPMLF	0.2936	1.2466	0.3933	2.2300	1.4171	✓	0.78	Non-toxin	Allergen

*Bracketed letters describe the location of the protein from which the epitope was obtained. The letter P indicates periplasmic origin, and O indicates outer membrane origin. The selection criteria included (i) TAP values, (ii) C-terminal cleavage, and (iii) antigenicity values. Bold lettering refers to CTLs that have been finalized for further review.*

### Proteome Mining for HTLs

For predicting HTL lymphocytes with high binding efficiencies, a seven-allele HLA reference set was chosen. When selecting epitopes for the final construct of the multi-epitope vaccine, only those with the lowest percentile ranks were selected. The final selected HTL epitopes as well as the location of the proteins used to extract these epitopes are listed in Table 2.

**TABLE 2:** Predicted 15-mer peptides as the potential helper T lymphocyte epitopes.

Sr. no	Position (Start-end)	Sequence (15-mer)	HLA allele	Percentile rank	Antigenic values	ToxinPred	Allertop
**1**	**24–38**	**AQFGLRMQKATQQLA (P)**	HLA-DRB4*01:01	**0.16**	**0.97**	**Non-toxin**	**Non-allergen**
**2**	**145–159**	**AFGPYVLNLSTRTLT (P)**	HLA-DRB3*02:02	**0.01**	**0.43**	**Non-toxin**	**Non-allergen**
3	11–25	KILVVDDDPRLRDLL	HLA-DRB1*03:01	0.03	Non-antigen	-	-
4	175–189	ADDAVEFSRTAPNMI	HLA-DRB1*07:01	0.03	Non-antigen	-	-
5	117–131	AQLIDLLRIYLGKKL	HLA-DRB1*15:01	0.08	Non-antigen	-	-
6	199–213	IVNYIRRNYGMLIGE	HLA-DRB3*02:02	0.22	Non-antigen	-	-
7	35–49	SSRVLVDTPEVRGMI	HLA-DRB1*03:01	0.16	Non-antigen	-	-
8	4–18	REGYRPNVGIILVNS	HLA-DRB3*02:02	0.05	0.45	Non-toxin	Allergen
9	393–407	GNFKSLLARMKEWFM	HLA-DRB5*01:01	0.10	Non-antigen	-	-
10	**54–68**	**KRHYKRIRSQQLPP (O)**	HLA-DRB4*01:01	**0.07**	**0.92**	**Non-toxin**	**Non-allergen**
11	71–85	EQLIARNAPLPIVFI	HLA-DRB3*02:02	0.11	0.72	Non-toxin	Allergen
12	54–68	RGLLRMVSRRRKLLD	HLA-DRB5*01:01	0.02	Non-antigen	-	-
12	98–112	RARIVAQAAQDAAQE	HLA-DRB4*01:01	0.38	Non-antigen	-	-
14	76–90	AVYGIRGILARGKFD	HLA-DRB5*01:01	0.06	Non-antigen	-	-
15	251–265	LQYQKHLRLQEARRL	HLA-DRB5*01:01	0.41	0.57	Non-toxin	Allergen
**16**	**114–128**	**KKSLARLQLDHIDLY (P)**	HLA-DRB4*01:01	**0.39**	**0.61**	**Non-toxin**	**Non-allergen**

*Bracketed letters describe the location of the protein from which the epitope was obtained. The letter P indicates periplasmic origin, and O indicates outer membrane origin. The selection criteria included (i) low percentile rank, (ii) antigenicity, (iii) allergenicity, (iv) toxicity. Bold lettering refers to HTLs that have been finalized for further review.*

### Study of Population Coverage

The epitopes and alleles associated with selected MHC class I and MHC class II epitopes were analyzed in various regions of the world (Supplementary Table 1). We found that the selected MHC class I and class II epitopes are shared by 57.55% and 98.9% of the world’s population, respectively. Among the countries with the highest coverage of class I MHC epitopes, Italy (88.28%) and China (81.39%) ranked first and second, respectively (Supplementary Table 2). As for the MHC II, the United States had the highest coverage of MHC class II epitopes (99.1%) (Supplementary Table 3).

### Proteome Mining for Linear BCEs

In the humoral immune response, B lymphocytes play a major role. To further incorporate B-cell epitopes into the final construct, we chose those with the highest scores. As a whole, scores greater than 0.9 suggested better potential for incorporation into vaccine formulations. A shortlist of ten optimal epitopes and location of their proteins can be found in Table 3.

**TABLE 3:** Predicted 20-mer peptides with values greater than the threshold value (0.9) as the potential B cell epitopes.

Sr. no	Sequence (20-mer)	Start position	Threshold	ToxinPred	AllerTop
1	NLQEHSVVLVR GGRVKDLPG	114	0.93	Non-toxin	Allergen
**2**	**ADITDKGIALT GGGALLRDL (O)**	**285**	**0.95**	**Non-toxin**	**Non-allergen**
3	VIADFTVTEQM LKQFIRMVH	81	0.91	Non-toxin	Allergen
4	ERGRASWAPYPD ITPLPPEH	121	0.91	Non-toxin	Allergen
**5**	**PLPPEHRTLEQ AWSIFRFGP (P)**	**135**	**0.96**	**Non-toxin**	**Non-allergen**
6	YGDLLRHFGRS IVIAHSEGA	195	0.92	Non-toxin	Allergen
**7**	**NADVQLDL LLDYASNVQKYP (O)**	**205**	**0.98**	**Non-toxin**	**Non-allergen**
**8**	**TGGSLPGTGSG GGGGPGGAG (O)**	**36**	**0.94**	**Non-toxin**	**Non-allergen**
**9**	**IDNGFDADPAT DHHKLSVAG (P)**	**156**	**0.97**	**Non-toxin**	**Non-allergen**
10	PADPVTPPDPAK PADPATPA	87	0.92	Non-toxin	Allergen

*Bold lettering refers to HTLs that have been finalized for further review.*

### Linking of Epitopes to Contrive Multi-Epitope Vaccine

**FIGURE 2:**
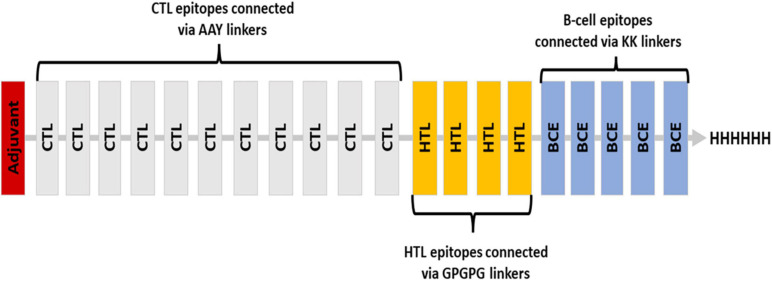
Blueprint of the proposed vaccine ensemble with different colors representing different components.

Various linkers were used to merge the 11 CTL epitopes, 4 HTL epitopes, and 5 B-cell epitopes. CTL epitopes were linked using AAY linkers (AAY aids the epitope in achieving good binding to TAP transporters as well as facilitates epitope presentation); HTL epitopes were linked using GPGPG linkers (GPGPG catalyzes the HTL responses and preserves the conformational immunogenic potential of helper and antibody epitopes). A further connection of epitopes to B-cell epitopes was made using the KK linkers. An adjuvant was placed at the N-terminus *via* EAAK linker, and a 6-his tag was placed at the C-terminus (Figure 2). In total, 451 amino acids were incorporated into the final construct.

### Inspection of Physicochemical Properties

Molecular weight of the vaccine was found to be 47.1 kDa. The theoretical PI of the vaccine was 8.33 which demonstrated the basic nature of vaccine. A half-life of approximately 10 h and 20 h was observed in *E. coli* and yeast, respectively. The vaccine was classified as stable by its reported instability score of 33.36 (< 40 represents stability). Moreover, the value 86.05 corresponds to thermo stability, which describes the vaccine’s ability to withstand a wide range of temperatures. GRAVY assessed the vaccine as a hydrophilic molecule with a GRAVY value of −0.114.

### Allergenicity and Antigenicity and Solubility Analysis

The final sequence of proposed vaccine was sent for allergenicity testing using the AlgPred and AllerTop servers. IgE epitope mapping and amino acid composition indicated the non-allergenic nature of vaccine. Furthermore, the antigenic potential exhibited by the designed construct was observed to be greater than the threshold value as measured by the VaxiJen 2.0 webserver with a value of 0.79 without adjuvant and 0.88 with adjuvant. Therefore, regardless of whether an adjuvant was present, the construct was antigenic in nature. As a result of overexpression in *E. coli*, SOLpro server had a solubility level of 0.949. Using the Protein-Sol webserver, the solubility of the vaccine construct (QuerySol = 0.49) was greater than the population average (PopAvrSol = 0.45), thereby confirming the soluble nature of the proposed ensemble.

### Investigation of Secondary Elements in the Designed Construct

**FIGURE 3:**
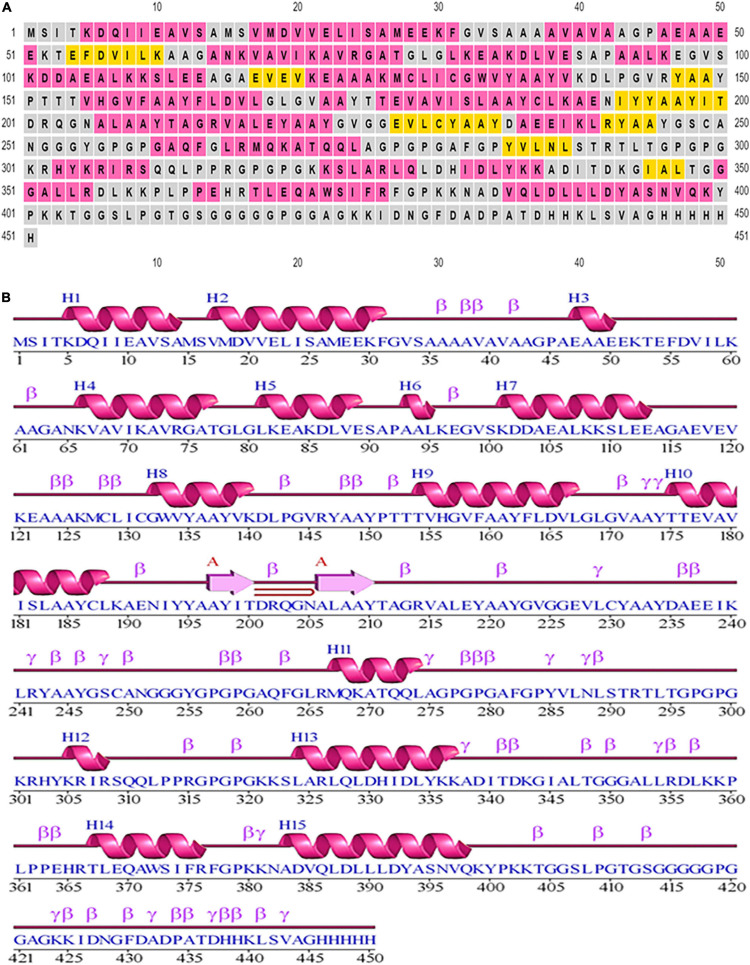
Secondary structural elements of the designed construct. **(A)** Annotation grid. **(B)** Illustration of the structure with each region denoted by its own symbol.

Based on PSIPRED, it was determined there were 9.3% strands, 48.11% helical structures, and 42.57% coil structures. Figure 3 is a diagrammatic depiction of the secondary elements of the designed vaccine.

### Tertiary Structure Prediction

**FIGURE 4:**
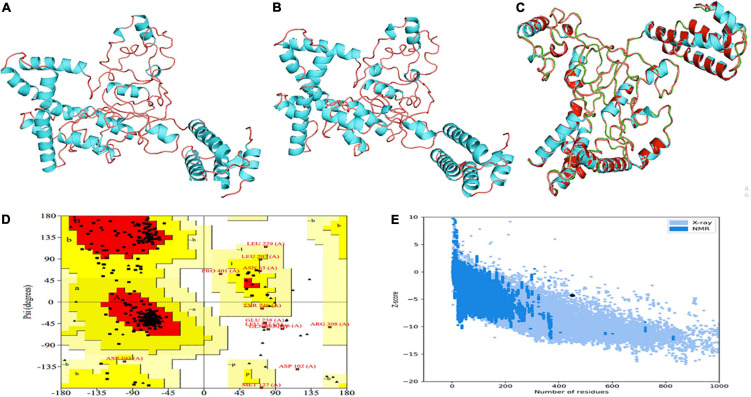
Projection, refinement and validation of the 3D structure of the MEV. **(A)** I-TASSER generated model. **(B)** Structure generated by 3Drefine server. **(C)** Structure improved by GalaxyRefine. **(D)** Ramachandran plot. **(E)** Z-score.

With the use of several threading templates (1rquA, 7s0yA, 1rqv, 7louA, 1rqv, 7mexA, 1rqv, 7mexA, 1rquA, and 7eeiA), the I-TASSER forecast the probable 3D model of the designed construct. These 10 templates showed good alignment based on their Z-score values ranging from 1.22 to 3.86. C-score values can range from −5 to 2, with a higher score denoting higher confidence. Among the five predicted models with a C-score between 0.58 and −2.70, model 1 was chosen based on its highest value. TM-scores are used to check for topological similarity between two protein structures. Our vaccine construct had a TM score of 0.58 ± 0.14, whereas RMSD score was 8.6 ± 4.6 Å. TM scores over 0.5 are indicative of accurate topology, while scores below 0.17 indicate non-specific similarity (Figure 4A).

### Model Refinement and Validation

**FIGURE 5:**
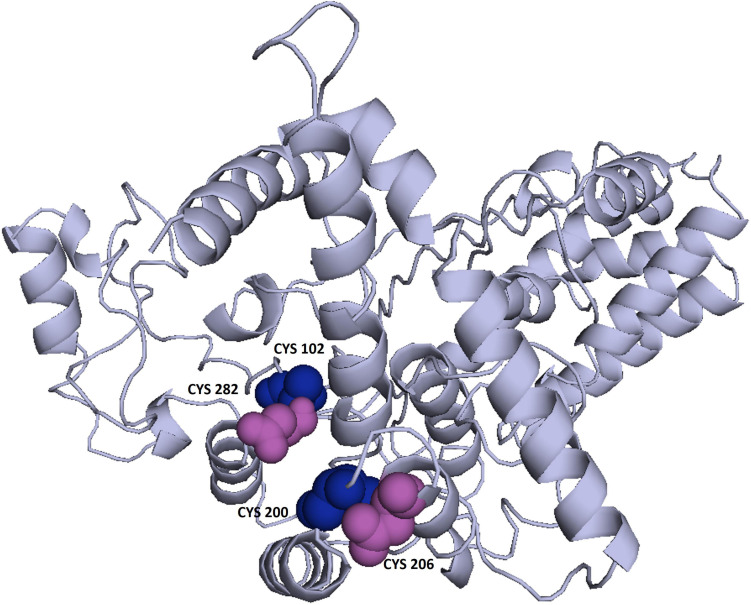
Enhancing protein stability through disulfide engineering. There are two sets of mutated residues displayed in magenta and gray. Residues were chosen according to their energy, chi3 value, and B-factors.

3Drefine was used to refine the potential chimeric vaccine model. Several parameters were used to shortlist one of five models generated by 3Drefine. Subsequently, the refined model was sent for further refinement by GalaxyWeb, where Model 1 was shortlisted based on poor rotamers score (0.6), clash score (9.8), and Ramachandran plot (90.2%) for further corroboration (Figures 4B,C). Based on PROCHECK’s Rama plot investigation of the protein model, 86.2 percent of amino acids were found in preferred regions. In addition, 10.5% of the residues were in the allowed regions, while only 1.4% of proteins were in the disallowed boundaries, so the model was of good quality (Figure 4D). To refine loops in the disallowed regions, 10 rounds of loop refinement were performed each for Val20-Ala26, Ala84-Glu89, Glu105-Ala114, Glu218-Val224, Cys230-Ala232, Tyr335-Lys337, Asp342-G344, Tyr393-Gln398, Gly423-Ily426 and Pro434-Gly445. Furthermore, we used Disulfide by Design v 2.0 (48) for the S-S engineering of the final construct to increase the steadiness of its modeled structure. It was determined that 32 residue pairs could be utilized for disulfide engineering. Nevertheless, after factors such as Chi3 and energy were evaluated, only four pairs of residues were considered final since their energy values and Chi3 values fell within the permitted range, which is less than 2.5 kCal/mol and between −87° and + 97°, respectively. Hence, a total of four mutations were introduced at residue pairs Asp102-Phe282 and Thr200-Ala206 (Figure 5). The ProSA-Web validated the overall quality of the refined model and any potential errors. With a Z-score of −1.26, the refined model appeared to be suitable (Figure 4E). Furthermore, the ERRAT web server gave a quality score of 84.32.

### Non-linear B-Cell Epitopes Determination

**FIGURE 6:**
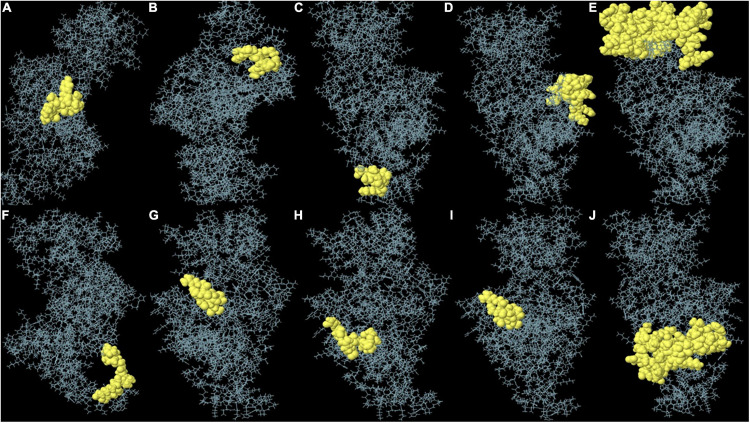
**(A–J)** Each monomer of the designed vaccine is predicted to contain discontinuous B cell epitopes.

Non-linear B-cell epitopes may be predicted based on the structure and folding of the new protein. By applying the ElliPro web tool, we were able to analyze the refined 3D models of non-linear B-cell epitopes (Figure 6). With values ranging from 0.51 to 0.79, ElliPro predicted 10 B-cell epitopes with non-linear characteristics involving 237 residues (Table 4).

**TABLE 4:** Non-linear B-cell epitopes as forecasted by the Ellipro webserver.

	Amino acid residue	Total residues	Score
1	A:A136, A:A137, A:Y138, A:V139, A:K140, A:D141, A:L142, A:P143, A:G144, A:V145	10	0.798
2	A:D392, A:A394, A:S395, A:N396, A:V397, A:Q398, A:K399	7	0.765
3	A:P362, A:Y400, A:P401, A:K402, A:K403, A:T404, A:G405, A:G406, A:S407, A:L408, A:P409, A:G410, A:T411, A:G412, A:S413, A:G414, A:G415, A:G416, A:G417, A:G418, A:P419, A:G420, A:G421, A:A422, A:G423, A:K424, A:D427, A:N428, A:G429, A:F430, A:D431, A:A432, A:D433, A:P434, A:A435, A:T436, A:D437, A:H438, A:H439, A:K440	40	0.747
4	A:M15, A:S16, A:V17, A:M18, A:D19, A:V20, A:V21, A:E22, A:L23, A:I24, A:S25, A:A26, A:M27, A:E28, A:E29, A:K30, A:F31, A:G32, A:V33, A:S34, A:A35, A:A36, A:A37, A:A38, A:V39, A:A40, A:V41, A:A42, A:A43, A:G44, A:P45, A:A46, A:E47, A:A48, A:E50, A:E51, A:L59, A:K60, A:A61, A:A62, A:G63, A:A64, A:N65, A:K66, A:V67, A:A68, A:I70, A:K71, A:A72, A:R74, A:G75, A:T77, A:G78, A:L79, A:G80, A:L81, A:K82, A:E83, A:A84, A:K85, A:D86, A:E89, A:K96, A:G98, A:V99, A:S100, A:K101, A:D102, A:D103, A:A104, A:E105, A:A106, A:L107, A:K108, A:K109, A:S110, A:L111, A:E112, A:E113, A:A114, A:G115, A:A116, A:E117, A:V118, A:E119, A:V120, A:K121	87	0.744
5	A:P319, A:K425, A:I426, A:L441, A:S442, A:V443, A:A444, A:G445, A:H446, A:H447	10	0.72
6	A:T293, A:L294, A:T295, A:G296, A:P297, A:G298, A:P299, A:G300, A:K301, A:R302, A:H303, A:A338, A:D339, A:I340, A:T341, A:D342, A:K343, A:G344, A:I345, A:A346, A:L347, A:T348, A:G349, A:G350, A:G351, A:A352, A:L353, A:L354, A:R355, A:P379, A:K380, A:N382, A:A383, A:D384, A:V385, A:L387, A:D388, A:L389, A:L390, A:L391	40	0.711
7	A:T200, A:D201, A:R202, A:Q203, A:G204, A:N205, A:A206	7	0.609
8	A:G223, A:G225, A:G226, A:E227, A:V228, A:G247, A:S248, A:C249, A:A250, A:N251, A:G252, A:G253, A:G254, A:Y255, A:G256, A:P257, A:G258, A:A261, A:Q262, A:L265, A:R266, A:K269	22	0.591
9	A:D356, A:L357, A:K358, A:K359, A:P360, A:L361	6	0.557
10	A:Q268, A:Q272, A:A275, A:G276, A:P277, A:G278, A:G280, A:R315	8	0.51

### Stability Check *via* MD Simulation

**FIGURE 7:**
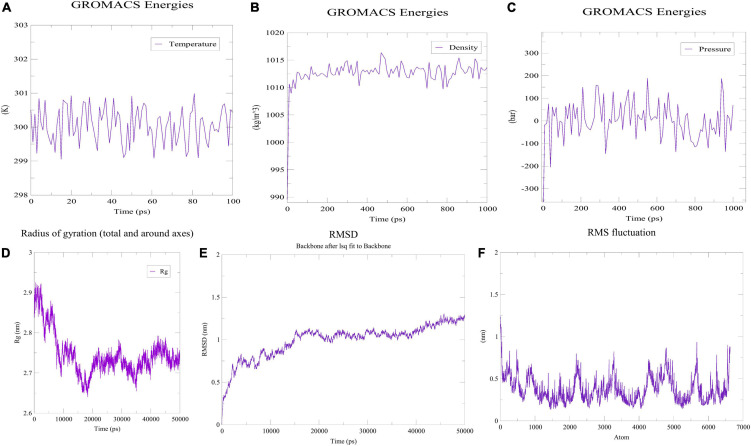
MDS analysis of the designed construct. **(A)** Temperature of the simulated system. **(B)** Pressure of the system. **(C)** Density attained by the system. **(D)** Radius of gyration plot indicating compactness of the construct around its axis. **(E)** RMSD graph depicting stability of the designed construct. **(F)** RMSF plot showing flexibility of the construct.

Prior to determining how the MEVC interacted with the immunoreceptor, the folding stability of MEVC was evaluated. The use of MD simulations to understand protein folding and stability could provide insight into different properties of proteins—their loops, their interactions with other proteins, and the effects of mutations on these interactions. The previously obtained three-dimensional structure was therefore simulated with the MD technique. Using the OPLS-AA as the force field, the vaccine construct’s mass was determined to be 47,011.99 amu. To neutralize the net charge on the protein, four Cl ions were added at atoms 65,580, 17,853, 21,750 and 13,863. The water molecules remaining behind were 43,756. Following that, 50,000 energy minimization steps were performed where steepest descents converged to Fmax < 1,000 in 1,455 steps. The potential energy was found to be −2.4462095e + 06 kJ/mol while the average potential energy was found to be −2.38951e + 06 kJ/mol with a total drift of −195,161 kJ/mol. The average temperature after 50,000 steps was 299.757 K with a total drift of 1.11929 K (Figure 7A), the average pressure was −0.466784 bar with a total drift of −0.403029 bar (Figure 7B), whereas the average density was 1,012.79 kg/m^3^ with a total drift of 0.55504 kg/m^3^ (Figure 7C). Trajectory examination was performed for 50 nanoseconds. Throughout the experiment, the radius of gyration graph indicated that the designed construct was quite stable (Figure 7D). In terms of RMSD backbone, there seems to be very few fluctuations, indicating the vaccine’s stability and structural integrity over time. The RMSD plot has been depicted in Figure 7E which showed that RMSD levels had gone up to ∼1.12 nm. On the other hand, high points in the RMSF plot suggested a high level of flexibility in the vaccine design (Figure 7F).

### Docking Analysis of the Ensemble With Immune Receptors

**FIGURE 8:**
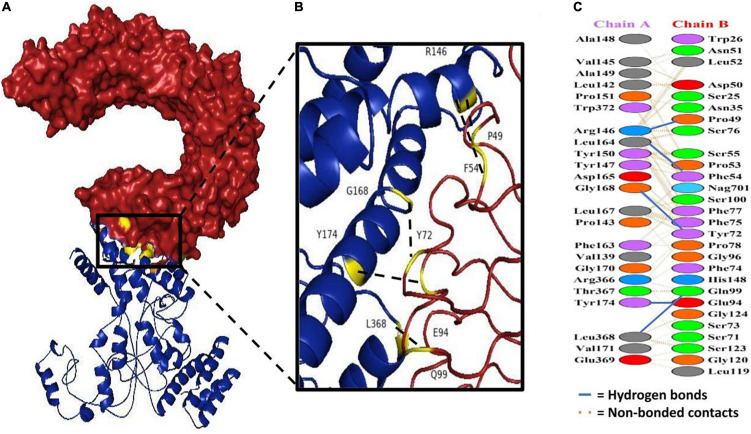
Docking study of subunit vaccine with immune receptors. **(A)** TLR4 chain B can be seen in red, while the vaccine construct can be seen in blue. **(B)** The interacting interface of receptor and designed construct showing all the residues involved in bond formation. **(C)** Protein-protein interaction residues across the interface.

Docking analysis between TLR-4 and the multi-epitope vaccine was accomplished *via* an online server, ZDOCK (54). Overall, ten vaccine-TLR combinations were generated, but only the optimal combination was selected. Fourteen hydrogen bonds were reported to be present between the vaccine construct and the receptor according to the PDBsum server (Figure 8 and Supplementary Table 3).

### Codons Optimization and Computational Cloning

**FIGURE 9:**
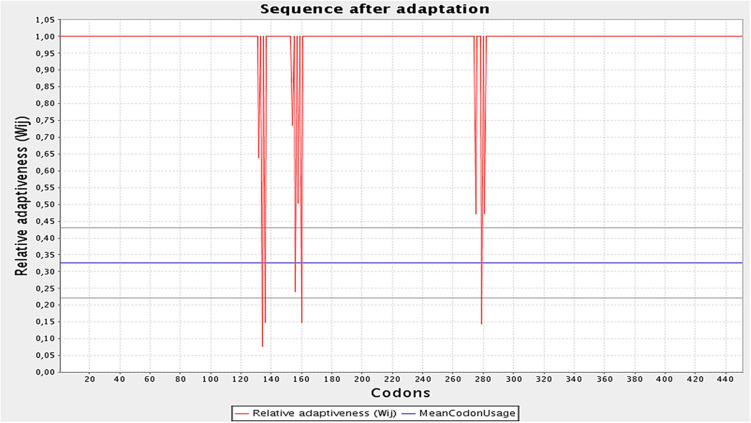
Optimization of the codons of the final design. Graph illustrates the codon adaptation index of the optimized construct to be 0.97. The GC levels were found to be 53.28%.

**FIGURE 10:**
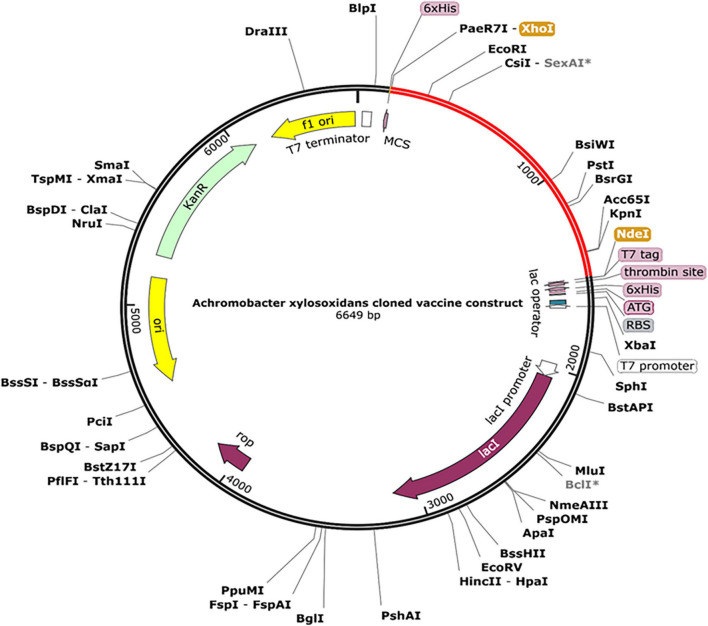
A final clone of the protein in *E. coli* plasmid [pET28a (+)] *via in silico* cloning. The red portion depicts designed vaccine, whereas the black portion is structure of *E. coli* vector backbone. The C-terminus of cloned construct contains a 6xHis tag.

The designed construct was tailored to use codons consistent with those of *E. coli* (strain K12) to maximize protein expression (Figure 9). Optimal codon sequence length was 1,353 nucleotides. GC content of 53.28% and a codon adaptation index score of 0.97 indicated the vaccine’s high expression in host cells. Finally, the optimized sequence was cloned into the pET28a (+) vector (Figure 10).

### Immune Simulations

**FIGURE 11:**
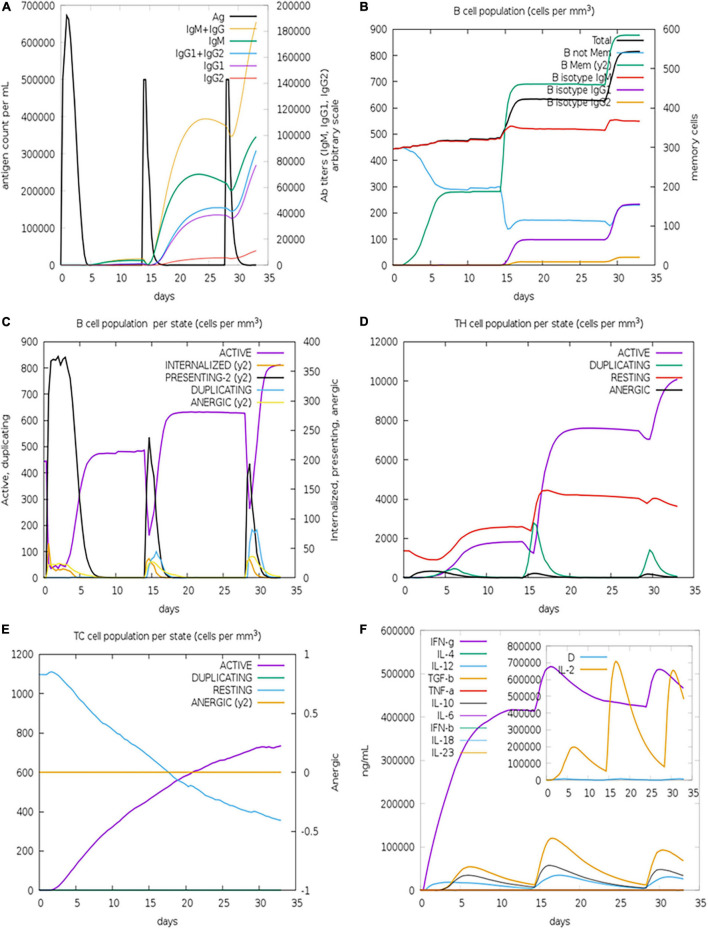
Immune simulations of the designed construct. **(A)** Immune responses to three doses of vaccination. **(B)** Increase in antibody generating plasma cells. **(C)** B-cells/state. **(D)** TH population/state. **(E)** TC population/state. **(F)** Cytokine production levels.

By simulating the *in silico* human immune responses to numerous doses of antigen using the C-ImmSim web server (59), we evaluated the immune system response. The simulation produced significantly more secondary and tertiary responses after injections than primary reactions. Moreover, the antigenic concentrations declined after doses, but antibody titers (IgG1 + IgG2, IgM, and IgG + IgM) increased significantly, indicating that the immune system had been greatly stimulated (Figure 11A). Additionally, numerous long-lasting B cell isotypes were identified, signifying potential switch-over between isotypes and possible memory development (Figures 11B,C). Similarly, a proliferating level of helper and cytotoxic T cells suggested the development of secondary and tertiary immune responses (Figures 11D,E). Likewise, we tested the Interleukin (IL) and cytokine levels. The high levels of IFN-γ and IL-12 prompted in the simulation further contributed to generating a consistent and robust immune response (Figure 11F). As a result, an effective immune response was shown to be elicited by the designed construct. Figures 11A–F illustrates the results as generated by the immune server.

## Discussion

This genome era brought with it a large amount of proteome and genome related data of nearly every clinically relevant organism. By using this knowledge, we are able to choose putative candidates for drug development, identify strains of bacteria or antibiotic resistance and develop diagnostic kits. Furthermore, *in silico* approaches improved the development of vaccines; vaccinomics is an emerging field that allows for *in silico* vaccine design. The *A. xylosoxidans*, like other Gram-negatives, elicit host immune responses *via* interaction with toll-like receptors (60). In this study, few immunoinformatic approaches have been employed to detect epitopes and design a construct that interacts efficaciously with toll-like receptors.

The whole proteome of reference strain of *A. xylosoxidans* was retrieved to carry out the study on epitopes prediction. So far, no studies have been performed to design a T cell and B cell containing multi-epitope vaccine. Here, we have performed a study to design a vaccine that is not only safe and potent but also stable in nature. Studies have reported that epitopes or antigens induce cellular as well as humoral immunity that is instigated by T-cells and antibodies, respectively (61).

The combination of multiple tools led to the selection of twenty epitopes, eleven CTLs, four HTLs, and five B cell epitopes that were non-allergenic, non-toxic, and immune potentiating. The selected epitopes were adjoined using linkers to finally construct a vaccine ensemble. Proteins typically require an adjuvant at the N-terminus in order to stimulate the immune response; we added ribosomal protein L7/L12 to enhance immunostimulatory properties (62).

After we proposed 3D structure of the construct, we sent it for additional refinement following which the Ramachandran plot informed us that more than 86% of residues occupied the favored region, emphasizing the modeled vaccine’s high quality.

The presence of TLRs (particularly TLR 4) is linked to the important role of recognition of antigens from Gram-negative pathogens (63). Therefore, our research investigated how designed chimera interact with these receptors. Using ZDOCK, we determined that the vaccine was a good ligand for immunological receptors, because when compared to the binding ability of the KDO crystal ligand (−622 kcal/mol), the binding energy of the vaccine construct was much greater (1559.49 kcal/mol) (Supplementary Table 3). In protein-receptor interactions, hydrogen bonds were found to be present that contribute significantly in influencing stability of complexes. The simulated immune responses elucidated the effectiveness of the designed construct in eliciting the immune response. Injections of vaccine increased memory cells whose levels persisted even after a third injection. The most preferred host is *E. coli* for producing more recombinant vaccine (64). A GC content of 53.28% and a CAI of 0.97 and was found using the JCAT tool, which supported the favorability of vaccine design for high-level expression in the chosen host i.e., *E. coli*.

## Conclusion

In the wake of recent emergence of *A. xylosoxidans*, our research focused on the possibility of developing a multi-epitope vaccine *via* computational modeling approach. As of now, no proper medical preventive measures such as vaccines are available in the market. Through *in silico* techniques, an effective vaccine can be developed in a shorter amount of time while maintaining low costs. Using immunoinformatic tools, we constructed a vaccine containing HTL, CTL and B cell epitopes that could instigate robust immune responses against *A. xylosoxidans.* Antigenic and immunogenic qualities were found to be present in the proposed construct. The MD simulations confirmed compactness of the designed chimera, and molecular docking studies showed stable interactions with immune receptor proteins. Finally, *in silico* cloning showed optimum expression and effectiveness of the construct in triggering the immune system based on immune simulation studies.

## Data Availability Statement

The raw data supporting the conclusions of this article will be made available by the authors, without undue reservation.

## Author Contributions

KK and YW: conceptualization and writing—review editing. KK, MA, and YW: data curation. KK, US, MA, MIA, and AA: formal analysis. KK, US, MA, and AA: investigation. KK, MA, MIA, AA, and YW: methodology. MA and YW: resources. KK: software. YW: supervision. KK, US, and MA: validation. KK and MIA: visualization. KK: writing—original draft. All authors contributed to the article and approved the submitted version.

## Conflict of Interest

AA was employed by the company PerkinElmer Inc. The remaining authors declare that the research was conducted in the absence of any commercial or financial relationships that could be construed as a potential conflict of interest.

## Publisher’s Note

All claims expressed in this article are solely those of the authors and do not necessarily represent those of their affiliated organizations, or those of the publisher, the editors and the reviewers. Any product that may be evaluated in this article, or claim that may be made by its manufacturer, is not guaranteed or endorsed by the publisher.
