# Supplementary material for: Immunoinformatic Approach to Contrive a Next Generation Multi-Epitope Vaccine Against Achromobacter xylosoxidans Infections
Source: Front Med (Lausanne). 2022 Jul 11;9:902611. doi: 10.3389/fmed.2022.902611 (PMC9309517; doi:10.3389/fmed.2022.902611)
Supplement: Supplementary file 1 [file Data_Sheet_1.docx]

**Bioinformatics-based proteome mining to contrive a next generation multi-epitope vaccine against *Borrelia burgdorferi*: the cause of Lyme borreliosis**

Table S1. Shortlisting of the putative vaccine candidates from the borrelia burgdorferi proteome

| Localization |  | Proteins (FASTA) | Virulent pred | Vaxijen | Algpred | Size (kDa) |
| --- | --- | --- | --- | --- | --- | --- |
| cytoplasmic-membrane | 1 | >WP_002556692.1 MULTISPECIES: ATP synthase subunit K [Borreliella]  MDIGLIGVNSALTISAIGSALGMGAAGSAAIGAWKRCYMQGKPAPFLLIVFVSAPLTQIIYGYILMNTLYEVMMQTNPWL  LLGAGIGGGFAIAVSGFAQGKAAAGACDAFSETGKGFATYLLVLGLIESVALFVMVFLMIFKFV | Non-Virulent | - | - | - |
|  | 2 | >WP_002556747.1 glycine betaine/L-proline ABC transporter ATP-binding protein [Borreliella burgdorferi]  MSRVAVKIKDCYKVFSYYVNKKQISRAIKDYEDGKDRMQIYKESSIFIANANINLDVYENEILVIMGMSGCGKSTLVRCL  NGIYKIDSGSILVNNMEMNAINRKDLSNLRKDKFAMVFQNFGLFPHMNVLRNVTYGLEVKHIPRKIREERAIEVLNLVGL  EDSKYKYINELSGGMKQRVGIARALVVNPDILLMDEAFSALDPLIKGEMQEELLRLVAKLKKTVVFITHDLNEAFKLGHR  IAFMRDGKIIQVGKPLEILANPSTDFISDFIKNLPVLNILKIKDILKDDFDLNSGGDNGLNVIIKCQGENFSLYDKALNK  RYDNLVSLNLDLNDEIKKIVEYLNKQDYLIIKEKGTVVGYISLNEISYLLSR | Non-Virulent | - | - | - |
|  | 3 | >WP_002556662.1 hemolysin family protein [Borreliella burgdorferi]  MLGFFNFKNKKKKKGSPENDLEDKSNIETSLIKNFNALKETIVKEIMIPRIGVIFVDYAKSKDELLKVVTSSSHSRFPVY  HGTIDNIVGIIHTRDILLHMCKKDFYEIDLKDIMRKVMFVPESKKTDSLLKEFQENHVHIAIVVDEYGGVSGLVTLEDIL  EEIVGDIQDEFDNELDEIVRLDDGSYLCDARILIEDLNEKLNLNLPNGDFDTLGGFVYDLFGRIPLKNEKVEYNNLVFSI  KNMHQRNIKVIKISEKEGL | Non-Virulent | - | - | - |
|  | 4 | >WP_002556755.1 hypothetical protein [Borreliella burgdorferi]  MIQIYFMSVSLNILGGIILAFPILIERISFFKIFEDFVNLINDNKKVKSIFGLLFLITSALEIIKPYKLPIIGNLIPAVS  LFLIGFILFLKQKLPTEVQNNKKYGKFKSILESNQQIIGIISITIGIIHFLAAEIPLL | Virulent | Non | - | - |
|  | 5 | >WP_002556784.1 MULTISPECIES: tRNA (adenosine(37)-N6)-threonylcarbamoyltransferase complex ATPase subunit type 1 TsaE [Borreliella]  MILEFKSEKKMINFSKSFFYPLPIGKIFVLSGDMGSGKTSFLKGLALNLGISYFTSPTYNIVNVYDFVNFKFYHIDLYRV  SSLEEFELVGGLEILMDLDSIIAIEWPQIALSIVPKDRLFSLTFKIVGSGRVVELNG | Virulent | **0.51** | **Non allergen** | **15.4** |
|  | 6 | >WP_002556815.1 phosphate ABC transporter permease subunit PstC [Borreliella burgdorferi]  MHLSLKTKRKIIEIIFKSFILISAIISSLSILFLGLFILKTGIMPFLNNKIKILNFLFSTNWDPTSNLQKSYGILAFIIN  SFLTTLFSILIALPIGLGFAIYLLEKAKGFYRQFLQTVIELLAGIPSVVYGFFGSTFIAALVKNIFQREDNLGYNLISSS  LILSIMIIPTIISVCYSSLKAVPKSYKFASLALAATDWQTIYNIIIPSASRGILAGTILAIGRAIGETVAVLMVGGGSPL  FIKNIFSPIRTLTVNIAMDMGYASGVHREALFSTALVLLLFSIITNLLKNFILSSNKGLKKK | Non-Virulent | non | - | - |
|  | 7 | >WP_002556818.1 MULTISPECIES: ZIP family metal transporter [Borreliella]  MIKSILDYLLTLHPVLLGLLGSTFTWFTTAFGAAAVFFFRKVDNKIMDAMLGFSAGIMIAASFFSLIQPAIERAEELGYI  TWVPAVFGFLVGAFFIYIVDVFVPDLDKLTFIDEDLTKHGKKDFLLFTAVTLHNFPEGLAVGVAFGALASNPDIQTLVGA  MLLTLGIGIQNIPEGAAISLPLRRGNVALAKCFNYGQMSGLVEIVGGLMGAYAVYSFTRILPFALAFSAGAMIYVSIEQL  IPEAKRKDIDNKVPSIFGVIGFTLMMFLDVSLG | Non-virulent | - | - | - |
|  | 8 | >WP_002556839.1 aquaporin family protein [Borreliella burgdorferi]  MNYTKFQEFISEFLGTFILLALGTGSVAMTVLFSSSPEIPGEIIKGGYTNIVFGWGLGVTFGIYTAARMSGAHLNPAVSI  GLASVGKFPVSKLLHYIVAQILGAFTGALMTLVVFYPKWIEMDPGLENTQGIMATFPAVPGFLPGFIDQIFGTFLLMFLI  SVVGDFTKKHSDNPFIPFIVGAVVLSIGISFGGMNGYAINPARDLGPRILLLFAGFKNHGFNNLSIVIVPIIGPIIGAIL  GATIYEFTLKNNKD | Non-Virulent | - | - | - |
|  | 9 | >WP_002556848.1 phosphatidylcholine/phosphatidylserine synthase [Borreliella burgdorferi]  MKNINLILAWLVHIFTASGLIVGLYSIISIVNGNYSLLLKLTVIGLIIDGIDGTMARKLKVKELIPEIDGTLLDNITDYI  NYTFIPVIFFYLGEFIEEKYKVAICIGILLSSAYQFSRTDAKTNDNYFRGFPSLWNLFVILNIIFKMEQITNLITMSICI  ITSFIPIKFIYPSKTKELRKITIPITIISCLIFVVSIFSELSTTALKMAKTVLILYFAYLTLASIYLTYKTRNR | Virulent | **0.52** | **Allergen** | **-** |
|  | 10 | >WP_002556868.1 MULTISPECIES: MinD/ParA family protein [Borreliella]  MEDQAQSLRDMMRLNGKFNFSVDEKVQNSKTRFIAVSSGKGGVGKSNIAIGLALKYSELGKKVLILDADIGMANVNILLG  VIPKYSIYHMIAQSRDIREVITKTEYNIDLLAGASGTMELLDLSDVDVNKFIKELLKIYEYDIVVIDTSAGISRQVISFL  LSSDDVVIVTTPEPTSITDAYGIIKVLSYKMENLKNLRLIVNRVANVSEAKGVAKKVIDISGQFLNLNIDYLGHIYEDQN  IRSSVFKQKPFVLVNPNSKASYCLDSIVATLEEVSLDNRKRRGVIGFILRFFGVE | Virulent | **0.52** | **Allergen** | **-** |
|  | 11 | >WP_002556874.1 flagellar type III secretion system pore protein FliP [Borreliella burgdorferi]  MRKCFNFFLFFSVTSLSFAQTKSLQTTNGLNFPFLNFDSIGGSEIAFSLQLLILLTIITLSPAFLVLMTSFLRISIVLDF  IRRALSLQQSPPTQIVMGLALFLTIFTMWPTFNSIYEQAYLPLKESKINFNEFYNKGIAPLRIFMYKQMSDGRHEEIRLF  MSMSNYDRPKNFSEVPTHVLIAAFILHELKVAFKMGILIFLPFIVLDIIVASVLMAMGMIMLPPVMISLPFKLILFVMVD  GWTLITSGLIKSFM | Virulent | **0.7** | **Non-allergen** | **29kDa** |
|  | 12 | >WP_002556879.1 flagellar motor protein MotB [Borreliella burgdorferi]  MALRIKKPSKCDEGSPDYMLTYGDMVTLLLVFFVTMFSLNDIIFQENVIRIMSASFTGAGFFKGGKTLDFSKLSYLSNSF  MSLPSTVRNKQASQTAKNKSMIEFIEKIQSKNIVVRQEERGIVISLAADAFFDSASADVKLEENRDSIQKIASFIGFLSP  RGYNFKIEGHTDNIDTDVNGPWKSNWELSAARSVNMLEHILNYLDQSDVKRIENNFEVSGFGGSRPIATDDTPEGRAYNR  RIDILITTDASLSFPKEIKQ | Virulent | 0.52 | Allergen | - |
|  | 13 | >WP_002556880.1 MULTISPECIES: motility protein A [Borreliella]  MNLASIIGWGVGFGAILISMAFTPTGLGVFWDLSSVFITVVGSFSALMASSEVVAVKKIPTYLGFFFRRNSYAKVSIIKI  LVELSEKARKEGLLSLDDELEQINDPFFKSGMRLVVDGADPEVIRTMLYLELDQMQERHKVGSDLFKTWAKLAPAFGMTG  TLIGLVALLGNLEDKSALGSSMAVALITTLYGTIMANLMFTPVQLKLEKIDTEEAAVKTMIIEGVLSIQSGDNPRILEQK  LMTFLTPKDRSQLNSSIGGE | Virulent | non | - | - |
|  | 14 | >WP_002556902.1 MULTISPECIES: phospho-N-acetylmuramoyl-pentapeptide-transferase [Borreliella]  MFYLLGLRLLKYITFRMAYATIFAFLLSLIVGPYIILKLKKLRADQILREDGPKRHLSEKAGIPTMGGILIFFCVFISLV  FWSNILNVYFLIMVFVMLGFAFLGFIDDFLKIKKKTSDGLKARFKIYGQIIFSFFSVGILYYFGGEHVSVIYFPFIKSFQ  IDLGLFYIPFGMFILISASNSFNLTDGLDGLAIGLSIVITGALIIIAYLTSRADFAAYLHIPNIKGSEELVIFLGALLGG  SFGFLWFNAYPAKIMMGDTGSLALGAILGMAALILKSEILFSILAGVFIIETMSVIIQVLVYKKTKKRVFKMAPLHHHFE  ELGWSEMQVVIRFWIIGLIFAIIALSTIKIR | Non-Virulent | - | - | - |
|  | 15 | >WP_002556930.1 ABC transporter permease [Borreliella burgdorferi]  MNSLEKQNEENNSKLERRAWSRFKENKLAFGSLFVIGFYISIAILQPILPIYKYHTQIVEHSDLPPSFQAAGELWYNKEK  KFIEKLAKKEKREINEEELKKLEDIKRKIENEVQIIDKKEVKIHKRVYLLGTDNLGRDLLARLIQGSQISLSVGFIGAFL  SMIIGTILGSIAGFFGGLPDKIITKTIEILYALPYLLIVIILMAIMERSIIGLFIALAFVSWLTVARVVRGQVQSLSSSE  FIQAAKTLGATNQRIILKHLIPNSIGMIVIFTTIRVPSFIMAEAFLSFLGLGISAPMTSWGELVQNGIATFVEYPWKVFI  PAIVMTIFLLFMNFLGDGLRDAFDPKDSI | Non-Virulent | - | - | - |
|  | 16 | >WP_002556931.1 ABC transporter ATP-binding protein [Borreliella burgdorferi]  MEKENILEIKNLTIEFRLKHTTIHPVSNVNLSVKRGEIRAIVGESGSGKSVTSMAILKLLPELTTVYKSGEILFENQDLL  KLSEKELLKIRGNKISMIFQDPMTSLNPFLRISTQLEETIILHQGLGKKEAKEKAIEMLKTVGVVNAEERIKHFPHQFSG  GMRQRVMIAMALSCHPSLLIADEPTTALDVTIQEQILLLIKNLSKKFNTSTIFITHDLAVVAEICDTVSVMYQGKIVEEG  TVEEIFNNPKHPYTIGLLKSILTLEHDPNKKLYSTKENPMKITKTSTEEF | Non-Virulent | - | - | - |
|  | 17 | >WP_002556932.1 ATP-binding cassette domain-containing protein [Borreliella burgdorferi]  MSSKKEIILKVENLMQTFTTGEDFLFWKNKQKVNAVNNVSFEVEKNKTLGLVGESGCGKSTTLRSIMQLYTPTSGNIYFN  GKNITKLSKKELLKTKKDMQMVFQDPHTSLDPRMTIKEIIAEPLEIYNENKILPKTKQEIEQRVNELTDIVGLHKSMLTR  YPHEFSGGQRQRIGIARALALNPKLLLLDEAVSALDVSIRAQILNLLKALQKEFNLSYLFISHDLAVVKYMSDKIAVMYL  GVILELAPRETLFSNPIHPYTKMLIASIPEINPEKRKNKNIKLDEQTLANIRKIHLSTQVHPKLEEVEKDHFVSKYLFDE  MNR | Non-Virulent | - | - | - |
|  | 18 | >WP_002556957.1 prolipoprotein diacylglyceryl transferase [Borreliella burgdorferi]  MPNYINYPSWLHPEVIQGIPITWYSLSYILIILISYKFIWYQIQSDNVDIKKEDYEIFMFSLVLGAILGGRLASTLVYDK  SGIYYSNPWLILLPFDQHWNFTGFRGMAIHGGFLGAIIAPLITINTKLKNTNVQKYFLKLTDYGSIAFSSGYILGRLANF  ANAELYGRVMKGGIIFPNAEPFDTNIPGVKEFASSVGLEISPHDLLINLPRIPSQLIEGFFEGPVTFLLLWFLFKKIKKY  DGFIFGVYVMLYAFFRFFIEYLREPDKELGFIITYKPITSLSEFSFLNISMGQILSLTLMLSGLIWIIVTKKIADKKIKN  NTNLAYKN | Non-Virulent | - | - | - |
|  | 19 | >WP_002556990.1 MULTISPECIES: preprotein translocase subunit SecE [Borreliella]  MFRFIKDSILELKKVTWPKYNEVVGNGKQVFWLVLFVSIFLGIVDYLMFLVVTYVF | Virulent | non | - | - |
|  | 20 | >WP_002557229.1 ABC transporter ATP-binding protein [Borreliella burgdorferi]  MDNCILEIKNLSHYYDNNGNKTLDNINLKIKKNEFITLLGPSGCGKTTLIKILGGFLSQKNGEIYFFSKEISKTSPNKRE  INTVFQNYALFPHMNVFDNISFGLRMKKTPKDIIKEKVKTSLSLIGMPKYAYRNINELSGGQKQRVAIARAMVMEPKLLL  LDEPLSALDLKMRQEMQKELKKIQRQLGITFIYVTHDQEEALTMSDRIVVMNEGIILQIGTPEEIYNEPKTKFVADFIGE  SNIFDGTYKKELVVSLLGHEFECLDKGFEAEEAVDLVIRPEDVKLLPKGKGHLSGTITSAIFQGVHYEMTLEIQKTNWIV  QSTRLTKVGEEVDIFLEPDDIHVMHKE | Non-Virulent | - | - | - |
|  | 21 | >WP_002557295.1 MULTISPECIES: hypothetical protein [Borreliella]  MKAFKVKNLRRFSNFIRILVIVLFLNSLLSLFVFLAGSYNIFVYNFQKFYLDLAIILSSVSFGLESTRLIFFYFLKNKKI  KYYLILIFSFIIFFIALVFKIFLSGNK | Virulent | **0.83** | **Non-allergen** | **12.7** |
|  | 22 | >WP_002557338.1 ABC transporter permease [Borreliella burgdorferi]  MKIDLKQSLSLSKKELKILFGTPTAYVVMLFFLIFINFSFIFLSGFFIKDNASLTSYFSSMPIILMLVLPALSMGVFSEE  HKTGSIELLYALPLSPQEIVLGKFITLKIFTLILFSLTLPLTIMTIFMGEFDLGIILLQYLGIILYSLSVLSMGTFISSI  TKSQIVSYILTVFTLILILFSGKLVMIFGKENIIGEILNFVSITNHFSYFNMGILNLSDFIYFITFTVTFLILSTHSITL  KKWR | Virulent | **0.53** | **Non-allergen** | **27.6** |
|  | 23 | >WP_002557352.1 CvpA family protein [Borreliella burgdorferi]  MIINDPVKITGIVDILIIIIFTSLGFRGFLRGFIKEISGFAEVFVLILLLYKKTEEFRRFVEPIIELSYIQALLVFFLLI  HIGFLILQSLIESIISQLKLLFFNRILGLVLGLLEAFGIIAIVVYIIHSQQIFKPEYFLKESKLLDYLNPGINYLFKISK  TK | Virulent | non | - | - |
|  | 24 | >WP_002655941.1 phosphatidate cytidylyltransferase [Borreliella burgdorferi]  MLSKVKRFAFFARLGTFLFFVPLILFLIFLDFKNYLFLNILIFIFSGFAAKEVNDLLKLKFKFSGLSSILSFFLGFAPPI  LTYIHFNVFYLGMNVIYYLFIALVFSNWIVDLVFIKEHEIGNFLSQATSILFILIYPGVLMSFTVSITTLPKAPFLMLML  FAMVSGNDTFAYLFGYFLGKNSYRPTIISPNKTLMGFFGGILFSVLTAIFAVVFRLINLSYGESIILGILIGVFTIIGDL  FESGLKRSAGVKDSGKIVPGRGGALDSIDSFLLTGPIFYLYLS | Non-Virulent | - | - | - |
|  | 25 | >WP_002655947.1 hypothetical protein [Borreliella burgdorferi]  MKKYLFFILFLISSNNLIVSYPLSFGGGFSYQFTNYTDKTGATKFAPNFTRADHGINLNLFFDANYVLFEMSYKEAFVVT  HNGRYFSLGLYGTYPMVFKEQVRMLFPLIGFKYAFDLSSNNFNLFFLSMGLAADLFIPDLDGLYIRPLFMLSISPFSNYK  NFSGLTTEIMLGFNIGWRFFN | Virulent | **0.85** | **Non-allergen** | **181** |
|  | 26 | >WP_002655959.1 hypothetical protein [Borreliella burgdorferi]  MRFIIAFLMILNQGFSNLFSLPPEDIIFESSYEVAIKKAQKLNKNVLILVGRDIKENLIKDFLNSFTNGEIIHKVSRKSV  FLVIDKDNEIFNKINLQKSPTIFFVDSKNEQIKAAYVGAVLSSVQFDKDFLSYVMGTIKSTSVLKKQKDYEINTADGRTF  FYKTLKGDWRLKFNGKDRKLVLFDTDLKEFLVFKDINENKLYAIPKSRIGNIYFSLLGNEEWKLFGKIK | Virulent | non | - | - |
|  | 27 | >WP_002655968.1 ABC transporter permease [Borreliella burgdorferi]  MTISKNVFSKFILKFLNSSAFVSVFALFVGFLIVGLVVMGLGHSPFRMYFIILEIIFSSPKHLGYVLSYSAPLIFTGLSI  GISLKAGLFNIGVEGQFILGSIVALIASVLLDLPPILHVITIFIITFLASGSLGILIGYLKAKFNISEVISGIMFNWILF  HLNNIILDFSFIKRDNSDFSKPIKESAYIDFLASWKLSPEGLAYRSSHPFVNELLKAPLHFGIILGIIFAILIWFLLNKT  IIGFKINATGSNIEASRCMGINVKAVLIFSMFLSAAVAGLAGAIQLMGVNKAIFKLSYMQGIGFNGIAASLMGNNSPIGI  IFSSILFSILLYGSSRVQSLMGLPSSIVSLMMGIIVLVISASYFLNKIVLKGVKRVKYNNILD | Non-Virulent | - | - | - |
|  | 28 | >WP_002655974.1 signal peptidase II [Borreliella burgdorferi]  MSAKSKQYFNIFVFIISLIFFDQLSKYLVAKYVKLGSIYFSFFDDFFRIIHVRNTGILFSMGSNIHYSLKKIFFLAMPIF  ILIFVFYLSLKERNCIARISLLLIFSGGVGNVIDRLFRPSGVVDFLDLKFYGIFGLDRWPTFNFADSYVVIGMILFLVYD  FFIKRKVLNK | Non-Virulent | - | - | - |
|  | 29 | >WP_002655975.1 ABC transporter ATP-binding protein [Borreliella burgdorferi]  MINVEKVTKMYGPFTALFNVSFKVEEGEVLGILGPNGAGKSTLIKILTSFHYPSKGNVKIFGKDIVEHSKEILQQIGYVP  EKLALYPELSVKEYLKFISEIKGVKKLKKEIDRVISIFKLKEVEDKLISQLSKGFRQRVGIAGALINNPKLVILDEPTNG  LDPNQIIEFKEFLRELAKESTILFSSHILSEVESICKRIIIVNNGVIVADDTKENIIKNKLKEIEIELIVSKKSENEKKI  FNSKNDIFSLIKLEEHEKDLNISLKLSQGKTEEDLFSYIVKNNIILKAMIPKHESLEKIFSKLTKEREK | Non-Virulent | - | - | - |
|  | 30 | >WP_002655982.1 flagellar biosynthesis protein FliQ [Borreliella burgdorferi]  MTAGHILYLIRISIENIIILSAPMLIIALIVGLLISIFQAITSIQDQTLSFIPKIIVILLVIVIFGPWILNKLMQFTYMI  FSQLQNV | Non-virulent | - | - | - |
|  | 31 | >WP_002656001.1 MULTISPECIES: Bax inhibitor-1/YccA family protein [Borreliella]  MIDLTQEKQEILIKNKFLAKVFGLMSIGLLISAVFAYATSENQTIKAIIFSNSMSFMAMILIQFGLVYAISGALNKISSN  TATALFLLYSALTGVTLSSIFMIYTQGSIVFTFGITAGTFLGMSVYGYTTTTDLTKMGSYLIMGLWGIIIASLVNMFFRS  SGLNFLISILGVVIFTGLTAYDVQNISKMDKMLQDDTEIKNRMAVVASLKLYLDFINLFLYLLRFLGQRRND | Non-Virulent | - | - | - |
|  | 32 | >WP_002656052.1 rod shape-determining protein MreD [Borreliella burgdorferi]  MATFFTYFISSAFLGKIFQHYFATYFYFSIDIFLIFLVFNSLNFIFNVGLLSSILYGLLMDYFTGLPLGFFVFGYTIIFY  FNNKIKLFMPKNMLSMTIFFILSKIILWFLAIVFYDFVDLKSFNYSIFNLDLIVNIMSINFLYPIQNYFTRNFYSFKEDY | Virulent | **0.71** | **Non allergen** | **20.9** |
|  | 33 | >WP_002656061.1 ABC transporter permease [Borreliella burgdorferi]  MSNIIIFLISETLINSQTLILAGLGGLISEKSGIINIGLEGIMTIGAFSGATVAYFTNDPLFSIFAGGLAGLVLAILHAV  FTIFLKSDQIITGMALNFLGPAIAVFISTLIFSSISTPPIEIKLPILFDGILNKTSFIFQIFGKRYSVYIAILSVVLFHI  VFKYTKIGLRINASGENPEVLESVGVSVNKIRFFCVLLSGFLAGVSGAVLTTVVASSYVQGVTGGQGFIAIVMLIFGKWT  PLGVLIGSFLFSFVKTLAIVLAQLPFFSLIMPPKMLVITPYLIIILSLIFFSKKNYAPKFLGITYKKH | Non-Virulent | - | - | - |
|  | 34 | >WP_002656065.1 magnesium transporter [Borreliella burgdorferi]  MIDIDELRIFLKEKSYSKIKEKFLKHDSFDIAEALKRLNGTELILLYRFLPKKIAVETFSNFDQSTKNKLANSFTNKEIS  EMIDELNLDDVIDLLEEVPANVVQRFLASSTEENREIINKFLSYSDDSAGSIVTIEYVELKEDFTVGKALDYIRRVAKTK  EDIYTYYITDDEKHLKGVIKIEDLILAKDDVILSSIMRSSGFYIVGVNDEKEDVALLFQKHDITSVPVVDNEGRMIGVII  IDDILEVIQSVNTEDFQMIAAVKPLDTSYLDTSILVMTKNRIIWLLVLMVSSTFTATIISNYQNLMLSLVVLASFIPLLM  DTSGNAGSQASALIIRELALGTVKVKDFFKVFLKEICVSILVGAILASVNFLRIVFFVAPHHSDKLKIAFVVSSCLMVSL  TVAKILGGLLPIVAKLLKLDPALMAGPLITTIADAITLIAYFNIAKWVLVSYAV | Non-Virulent | - | - |  |
|  | 35 | >WP_002656087.1 RIP metalloprotease RseP [Borreliella burgdorferi]  MYILFSVLALSFIIFIHELGHFLFAKLFKVKVEVFSVGIGPSILKFKINNTEYRLSPILLGGYCKLKGFDHLEKELKANK  ELEADKDSLFGISHFKKILIYFAGPLFNLIFSFVVFIFISMAGVIYFDYSSRVSILNKDSFLKDKFRDGDVILKVNNKKI  EYFSDLRKFIPEEKSTVTFDVLREKENITFKETISLQDFLKEIGPWADLVIADVVSNSPAKIAGMKPGDEIISIDNVILK  NKRDLDYFLKNLNSDVVEIKFSRNGEIFSSKLVFHDKNKMIGIYFSPPLKRVVKVENVSSAIKNSFFKVVSALQDILYSI  FLLMTNFLNTSKSVSGPVGIVGILSSSYSLGILYWINSISFLSLILAGMNLFFIVIPIFDGGQIFISFIELLRGKRFKAK  TIYSFYSFGIFFGLFLFGLGLFNDLKGLLSIFN | Virulent | non | - | - |
|  | 36 | >WP_002656210.1 cation:proton antiporter [Borreliella burgdorferi]  MNKKIFYITILLHLPNLLFSYSTRYDIEVKMSAFVMSLAIIVISSISIGNLVAKLGIPKVIGQITAGIILSPNAFGKIQI  PLLFPLGITQIGENYLINEKIFAISTIASIILLFTAGLETDLKLFIKFLPRGGIIGITEVVGTFTSFVLMASIIFNVPLI  SPTSLFIGIIGTPTSAGIAASILSAKKKMSTSEGVTIISTSIIDDVLSMLMLTSVITISRSISDLDIASSIKAIVQNIVI  WLCLTFSLIYISETLSRLLKKLNSVTLATVITLSLALTIASIFQNLGMSFVVGAYVFGLAMSKTDIVYVIQDKLTIFERF  FIPIFFTSIGLMSDINEILSKEVLILGLAISAIAIITKSIFCFIPALFLGFNKLGALKIATGMVPRGEVSLIMANVALSS  GFISQKIFGIIIIMVFLPTIIATPIINFLFKINKSGLKKELPIDQNTHICVSFEYDNLAKILIWDFKNELRKEGFFTQQI  KNDSSQYINARKNNISFSIKREGSKITFECPNNHLIIIQDLFRETILNLEKITKEVETVSLRAKKLDYSINYDKILSNIN  LNKRIKKENIILELKSSNKADVIRELLSVINIEIDKERIFQDLMEREKLITTALKEGFAIPHLKTNLISKIHIAIGISHE  GIDFNALDKNLSHVFILILCPAKDYVSYPRILASVVGKVDLYKKEILNAKTDKEIYNIIVS | Virulent | non | - | - |
|  | 37 | >WP_002656216.1 preprotein translocase subunit SecY [Borreliella burgdorferi]  MKELFLSLFTVKDLRNKFLFTLFVLFLFRVGSYLPIPGIDSVALKSYFKSQSDFSIANYFDFFSGGAFSNFSIFMLSIGP  YISASIIVQLLVYSFPSLKKMQEGDGGRQKTKKYTKYLTIVAAVVQGYATSLYAKGIPGAVTIPFYRYIFVAILTVTTGT  FILLWFGEQINQRGVGNGTSLIIFSGIVVRLQAALFNLFQSMQDPSQNVNPVFVILIISIFILVVILIIYEYKAQMRIAI  HYARANSNSTVSSYLPIKLNPSGVLPVIFASVLITLPLQILSGFAETSSIARQILSYLRPNGFYYTFLNVILIIGFTYFY  SKIQLSPKDISNNIRKNGGTIPGIKSDEMEKYLDEIMNKTLFSGSIFLSIIAIIPFLVQNIFRFPHDVSRIMGGSSLLIM  VGVALDTLIHIDAYLKTQGFSHGNKKNYAFLQKI | Non-Virulent | - | - | - |
|  | 38 | >WP_002656245.1 maltose/glucose-specific PTS transporter subunit IIC [Borreliella burgdorferi]  MLKGFEQAQKFGRSFMLPIAILPAAGLLLGIGGSLSNPETVRTYSFLNIFFLQSVFKIMSASGSIIFSNLAPIFSIGIAV  GLAKSDKGTSGIAAFIGYLVMNATIGVLIDVSGRAESFSSGAVGFVLGIKTLETGVFGGVVVGILTYYLHSRFNKVDLPK  VLGFFSGSRFVPIIVSFSSIFLAVIMFLVWPFVQSGINKVGGLVDSTGYIGTLIYGIFLRMLGPFGLHHIFYLPFWTTGL  GGSVIIDGKLIEGTQNIFFAELAAQGTDRFFIGTSRFMSGRFITMMFGLPGAALALYYTAKREERTKVFGLLMSSALTSF  LTGITEPLEFSFLFVAPILYVVHATFDGFAFMLAHILQITIGQTFSGGFIDFILFGILQGNSRTNWLLVPVIGIVWFFLY  YFTFIFLINKFDFKTPGRTQDLNSEDSPSSKSSEFEENYATKVIIGLGGASNIVELDCCATRLRITVRDVLKVSEKILKK  TGSKGVIIKGNGVQVVYGPGVSVLKNEIEELLEA | Non-Virulent | - | - | - |
|  | 39 | >WP_002656250.1 ABC transporter permease subunit [Borreliella burgdorferi]  MKTDTIIKKIYIVLFNIFIVLLIITPSLVNENSKIAIYKKDPNKVYLKSIKNVPMPPTKDNPLGIDKMGRDIMARLIIAT  RNSILLSLSYATISAIIGIFIGTIIGMFSFEICMLISKPIETLQTLPFFYVVSLVFYYFLKQKTYNMLQTATLLALIHGW  IRFAFIARNNTLIIKNLDYIKASEAMGASKIRIILYHIFPEVFSSISSIIPLQMGRSLTTFEVVSFLQKQDKNLYPSLGE  LLNYMQMGNKYLWIWINPLLILIGINIILAIINFKLRKKMKHLISS | Virulent | non | - | - |
|  | 40 | >WP_002656309.1 protein translocase subunit SecD [Borreliella burgdorferi]  MKKGSKLILILLVTFFACLLIFPTLKWYFLMSVEDKKISSYSQEALRDYSKKKALNDLVKLKELYNKDPNSSIPASLSYL  IPIAKNNYRSSMKIPPNIFTAKTLREGFLTDSDMGEVSLEIYRYYENIKKGKSRIIHLGLDLSGGMSVTISLDYSSVEKK  LGRSLTFAEREDAIYRIMQILKDRVDRFGLTEPKIVREAGGNKIFLDIPGEKDESRVSTLLSGKGNLTFYVVDDESTSLL  HRKILEAGSLFSIPEIQASMNLPDSKQIFPWYVKDSYGVDDESSVRYYVVDASPENSFDGAHIKDAGVSNDPRTGRDTVA  FSLDVDGSEKFFKFTQKNVGKSLAVVMEGKIKSVAGIGYAITGGNVSIQGDSFDKKEAQDLALVFKTAAFPVDIKIDDLR  IIGPTLGARTIDLGIKASALALCLVFLFMCFYYGLSGVVAGFSLVIYNVFLILAILSAFNFTLTLTSIAGLILTMGMAVD  INIVIYERIKEEIREGRRFENAFEDGFKKAFLSIMDANITTFIAVLFLTLLGTGVIQGFAWSLSVGIVASLFSSLIFSRF  ILEFIISVRKSKFISISWSSKYAKSN | Non-Virulent | - | - | - |
|  | 41 | >WP_002656384.1 P13 family porin [Borreliella burgdorferi]  MNKLLIFVLATFCVFSSFAQANDSKNGAFGMSAGEKLLVYETSKQDPIVPFLLNLFLGFGIGSFAQGDILGGSLILGFDA  VGIGLILAGAYLDIKALDGITKKAAFQWTWGKGVMLAGVVTMAVTRLTEIILPFTFANSYNRKLKNSLNVALGGFEPSFD  VAMGQSSALGFELSFKKSY | Non-Virulent | - | - | - |
|  | 42 | >WP_002656398.1 ABC transporter permease [Borreliella burgdorferi]  MKIFILKNTIYLLINLICASFFCVSLVNLFSNEQQYTPFVKTNVIKNYLQYIGVYKSIERYALIHDFNPKSKLEKDCFLK  HIAGNSYIIYKTKNEGMLWGDHRYSLLSKGKPTTKIIFQKIFNTLKISIPGALLSYIAAIILIIIWKIYIKNNLINNILE  YLMLLLHSMPRNLTVFLILSLIYYLNLNPKNLIMGGFAWFFSFFIFNSVIFKQSLDKTLSEFYIKAAKSRGINKLQIILK  HALIPSITPLLTNMRPIITTAFFGASMIESMFEIDGIGALYLNALKFNDYAISKDLIFIGVFIMLIPNIITDILIYKINP  YKDTLN | Virulent | non | - | - |
|  | 43 | >WP_002656414.1 D-alanyl-D-alanine carboxypeptidase [Borreliella burgdorferi]  MNSIYVIGKLLLTLFLIFFPFCYNLFAVNLAEINKLSEYAKSIVLMDFDTKRILYSKKPNLVFPPASLTKIVTIYTALIE  AEKRNIKLKSIVPISDSASYYNAPPNSSLMFLEKGQIVNFEEILKGLSVSSGNDSSIAIAEFVVGNLNSFVNLMNINVLN  LGLFNMHFVEPSGYSSENKITALDMAFFVKSYIEKFKFMLNIHSLKYFIYPKSRNLGTALSSKFLNLKQRNANLLIYDYP  YSDGIKTGYIKESGLNLVATAKKGERRLIAVVLGVEKGINGFGEKMRSSIAKNLFEYGFNKYSKFPLIVKLKEKVYNGTV  DTVALFSKEPFYYILTKDEFDKINISYTVDKLVAPLSGDMPVGRAMIFLENEKIGDVALFSGKVKRLGFWQGLYKSFINL  FSREY | Virulent | Non | - | - |
|  | 44 | >WP_002656426.1 hypothetical protein [Borreliella burgdorferi]  MVEKIRLGFNIIFLGLFIYFLAILRFQMKLSFILFNYQFIVVYFLLVIVFNVIYSQYFFPRLYFILNGMEDTFTFLKLKM  VRKKLKSVFEISILLRFILIKQDKKSLDELYFYLKDTRLRHKTIIELYSVLISFREKEKASSLILNYKYSRNKWVKYCEA  LSMLSFEEHSKLKELVNFLDKFFLKNDIFTIYFYYLLRKSKTSFDLLESKKIEIRNRYYKFKNRIDSKHTKLLGSNLFFV  VFYYIYDFSKKDVFY | Virulent | 0.79 | Non-allegern | 31.4 |
|  | 45 | >WP_002656446.1 MULTISPECIES: flagellar motor switch protein FliM [Borreliella]  MANNPGALSQDDIDSLLESINSSESLSLDESLSNVISSPTGKKQKVKVYDFKRPDKFSKEQVRTVSSFHEAFARYTTTSL  SALLRKMVHVHVASVDQLTYEEFIRSIPNPTTLAIINMDPLKGSAIFEVDPTIAFAIVDRLFGGDGDTIKDKSRDLTEIE  QSVMESVIIRILANMREAWSQVVDLRPRFGHIEVNPQFAQIVPPTEMIILVTLEVKIGKVEGLMNFCLPYITIEPIVSKL  STRYWHSLIGVGTTSENLDALREKLENTAMPLVAEIGEVKLKVREILSLDKGDVLNLESSLINKDLTLKVGTKEKFKCRM  GLMGNKVSVQITEKIGDIKGFDLLKELTEEVE | Virulent | non | - | - |
|  | 46 | >WP_002656468.1 proline/glycine betaine ABC transporter permease [Borreliella burgdorferi]  MTKDFFILKIDNFFDFLVDNFSISDGVGFSKSIIFLYENLKNLFLFVNPLLFILTVCLLSFVFLKKRLIFLILPGFFFIL  YFNLWEASMDTIAIIFVSVLVSVILGIPIGILGGYFPRFYVFLKPILDLMQAMPPFIYLIPAIPFFGMGTASAIFATIVF  AMPPVIRYTRLGIVQVSDEVIEAAKSFGSSNLRILFQVQLPLSLQSIIEGINQSIMMAISMIVIAAMVGSSGLGRTVIYS  IERLNFGEGLISGLAVVIIAIILDRIMQSIFIKFSYLNTDHYGGKKENKFKRFLEIYNK | Non-Virulent | - | - | - |
|  | 47 | > WP_002656477.1 SEC59/DGK1/VTE5 family protein [Borreliella burgdorferi]  MFDEFKRTILREDIKYEIFRKFFHIFSLIVLVFYRINFWIGLFSNILFMILYLSSEIFRITEKKILFFKNISNIILKSRK  ILPNKVSFSPVFLFLGILISYCLSMHPFNYIGIFSVCLGDGFASLIGKLIPSFKLVNGKTISGSLVVFCVTFFSYYYFFP  YLTVALILGILAVLVELFDAANYDNLFLPLVVSASSYFLTSFFYSQ | Virulent | non | - | - |
|  | 48 | >WP_002656487.1 ATP-binding cassette domain-containing protein [Borreliella burgdorferi]  MVEFKNIVKYFPDIDKPILDSINLKIGEVKIFTVVGKNGEGKSTLAKIIAGLIEFDEGEILVNGIKQKNWNVDKAKNNGI  YLVSQVPNLKMNLRVWEYLSIYWFGYEFFMPMNKSKTYKYYRWLMQFYKISFDLDKKIKDLNIKEIYFLLIIAALKENAK  IIIFDESAAYFSQKEAQAFIKLLVLLKKSGVASLFITHSEITDAIKFSDEFIILKDGKCFRTVNKESILSKLESSSDKVF  VANINCNKFEKDPIKFNLFFEDFWKYDVSFSLNKRGVLGIIGEEAVIKTWEKLFLGELIFVGCIKIDGIRYERINIFECK  AGFLPLGIGNLFPDNSSILDNFLAKFMNFENKIFIRQSYINQIKDFFKKKMEFYSEEKIYRILYSKSLAFSGGTLKKFAL  YREMYIAKSFLICFSPLSNLDHKAYNEMSVAIRNYSKEKPVLLITSNLDELLLLSDNILAMKMGEVLLNVSREKISKEKL  KELLFL | Virulent | non | - | - |
|  | 49 | >WP_002656519.1 extracellular solute-binding protein [Borreliella burgdorferi]  MKKVIILIFMLSTSLLYNCKNQDNEKIVSIGGSTTVSPILDEMILRYNKINNNTKVTYDAQGSSVGINGLFNKIYKIAIS  SRDLTKEEIEQGAKETVFAYDALIFITSPEIKITNITEENLAKILNGEIQNWKQVGGPDAKINFINRDSSSGSYSSIKDL  LLNKIFKTHEEAQFRQDGIVVKSNGEVIEKTSLTPHSIGYIGLGYAKNSIEKGLNILSVNSTYPTKETINSNKYTIKRNL  IIVTNNKYEDKSVIQFIDFMTSSTGQDIVEEQGFLGIKT | Virulent | non | - | - |
|  | 50 | >WP_002656553.1 MATE family efflux transporter [Borreliella burgdorferi]  MIKKFKNYDQIIKELFVIAIPTAFESLLFQMVTFFDNFMISYLGSFHVTGVSLANRVTFLFFIIVFGLGTALSAYVSQAI  AKKKFLQVRQSFAYILLIGTTIGIIFFIFSFLFPRNIIKLFTANQDSLNFGSEYLKIISLSYVLMAYSFLSAMGFKSAKE  VKIPLYVTSIVVLINIVFNYILIFGFSMGIKGAAYATVLARIVEFVFYFLYSLISSNSYYRIKFGDFFAPKVVTRANLKL  IIPVLSHEIFWVLSITILHAFYARVGSIEYASFAVASNLFDICFVLLHGMGLATGVVIGHLMIYDKKHVRSVGFFLSFLG  FLLGIFVVILLIGISSFAPYIFSKLDSPELVSVFIYVFASIVIFKAFTAQVLVGVFRASGIPNVCFFIESGVIVFYTLPV  AYLLVFYTNLSLPIVVFIVNLEEIIKNIFIIIEFFHDDWIREIEYEELA | Non-Virulent | - | - | - |
|  | 51 | >WP_002656570.1 LptF/LptG family permease [Borreliella burgdorferi]  MKIDKLFIKSIILTFLSMNLLFMILIMLGDLFVNLLNYLEKNIGLKDILYIYYLYLPKAFSDGVALSFLFAISNLIGNLS  MRNEIIGLFSCGVPLTRILKPIILISIFISVVLFFFDNYLVIDTIARRDVLIKNSIGDSRSGDKTIIIRDFAREIYNIKS  YDIDENTFANLMIIIKDNKDEFQTRYDINKAEWKDNKWRLYGIREFVKVGKKIKENAYDVLDGTGIIKLAPDYIRTVMLS  SKALNFTKLINWISFLKAERLNYSDAFFDLLNRIFFSFRLILLSFTVGFIALALKKNIFILSLLNSIAFAVVYVISIVIF  NFLADLGYLHIYIASSFTTIFFLIINFFVYRIVRK | Virulent | non | - | - |
|  | 52 | >WP_002656613.1 DUF3996 domain-containing protein [Borreliella burgdorferi]  MRTKIIIMTIIILLAPISGFSNSKESARGKFGAGIILPLPIALQINIGNFDLDIGLYSGVNNLFSDWKTLFIALDYIFYI  YTFPGAANILDFSVGAGGYGTIWFSRFGGSKSGSGPMSIGARLPLALNIAVFRKKFDIFLRIAPGLGMNVWSNGVGFRWE  VFAGLGLRFWFT | Non-Virulent | - | - | - |
|  | 53 | >WP_002656679.1 chromate transporter [Borreliella burgdorferi]  MTKQMYKKKQKKIYEILDLFLLVFKTTTLTIGGGLIIISELKKIFVKKRKIISEDDFNKILATSNVIPGVTAINFVFLVG  RKFGGFPCALLLVVAGILPSIIAIIMVFLYLKLVPDSIHVKKFLEGAKISSIIIMITVVLKFSKKMLNDSIIKWTICFLV  IFAIFKLKIKISYILLIFFLVYTFKYITIKKILTK | Virulent | non | - | - |
|  | 54 | >WP_002656686.1 MULTISPECIES: hemolysin family protein [Borreliella]  MLELIIILLFIILSAIFSASETAYTSLSIIQIQDIRKKGKSGISVYNLVQSPSKLITTILIGNNISNIVASTLTTKFVLE  KYGNSALAISTGLITIIVLIFAEILPKQIAILNNEIIALSTSFFLKPLIFIFTPLIYIINKIIKKILNLFKVKTSSQMTK  ESIKNMLSLAGSLGILKNDSRIFMQKMLDIDQVRASEIMTHRTGVFSLSSSSKLKDVIKLIKEEGYSRIPIYKGQSREQI  IGILIAKDLIEVNKKDMNKNVSQFIKPAVFVQQNKRIKDILDIMRKKQKIMAIVIDEYGGFSGILTIEDIVEKIFGAISD  EYDIKEEKPLITQINDNTYSILGETTFDEIEEAIGISIKHKEYTNTIGGYLIDLLDKIPTKNETVKTNDGEYFIKEIQNN  KIETITFIKSKK | Virulent | non | - | - |
|  | 55 | >WP_002656803.1 J domain-containing protein [Borreliella burgdorferi]  MPSPIRVFFLVLLFIFIFNPVLIAMLFILFPFILILFSFLGVFRIYFTRDYSYSRSREFEFYKLSFLLMAKLLSILGTVT  GEQLNYVNFIINSLNLSERGKSELYTIFHSAITKNNNADKILYTLKLGYFQHKDLFIWLFATLKEINRLSRYKNLEAEKF  ISYVGVFLELESDGYEAYKDINIKIVNPYSVLGLTYSASDDEVKKAYKSLVIKYHPDKFANDPVRQKDANDKFIKIQDAY  EKICKERNIR | Non-Virulent | - | - | - |
|  | 56 | >WP_002656819.1 hypothetical protein [Borreliella burgdorferi]  MNLQKYLFLTALLLISTSVFAQTNTIAKENVIPNGNLSQFGVEEICPICGYVNCICDEQSTEIAAKVSQSNTTGFFTSIA  ILALLVITGLALAKKKLYKSKLKI | Virulent | non | - | - |
|  | 57 | >WP_002656831.1 YggT family protein [Borreliella burgdorferi]  MVVLIQILMVFLQIYRILILIRILLSWLVSSGINTNVFFRFIHVVTEPFLSFFRKIPLFTFGMFDFSPIAALITLTIFER  MLTYGNYKLSTFIILFIVEVWEIFRSIFIAIVFFLLLRLLLLLLNLFQGSDFFKTVDSFLLPLSTKISGVITDKHMSYTL  RLIVGSALMVAFIIIIEQVVFAIRALGTYLPF | Virulent | 0.63 | Non allergen | 22.2 |
|  | 58 | >WP_002656884.1 L-lactate permease [Borreliella burgdorferi]  MNSYDFITALVPIILIIIGLGIIKKPAYYVIPISLIATVAIVIFYKNLGIVNTSLAMLEGALMGIWPIATVIIAAIFTYK  MSEDQKDIETIKNILSNVSSDRRIIVLLVAWGFGNFLEGVAGYGTAVAIPVSILIAMGFEPFFACLICLIMNTSSTAYGS  VGIPITSLAQATNLDVNIVSSEIAFQLILPTLTIPFVLVILTGGGIKGLKGVFLLTLLSGMSMAISQVFISKTLGPELPA  ILGSILSMTITIVYARFFGNKETTERQSKNTISLSKGIIACSPYILIVTFIVLVSPLFNKIHEYLKTFQSTISIYPEANP  LHFKWIISPGFLIILATTISYSIRGVPMLKQLKIFTLTLKKMALSSFIIICIVAISRLMTHSGMIRDLANGISIITGKFG  PLFSPLIGAIGTFLTGSDTVSNVLFGPLQTQMAENIGANPYWLAAANTTGATGGKMISPQNITIATTTAGLIGQEGKLLS  KTIIYALYYILATGLLVYLV | Virulent | non | - | - |
|  | 59 | >WP_002656931.1 ABC transporter ATP-binding protein [Borreliella burgdorferi]  MENILIIKNLCKAYKKNKTKIQVIENLNLTVEKGEFISIQGKSGCGKSTLFNMISGIDKIDSGEIISCGIFLKNANEKTL  SLYKNRQIGLVFQNYNLINEFSVIENIILPQIISGQKTKETINKKALDLMKILKIENRANHYPSELSGGESQRVAIARAL  INEPNIILCDEPTGNLDLSTAKTVENLLINTAKNFKKTLILVSHNPQFANKADSKYEFKDRTLKKL | Virulent | non | - | - |
|  | 60 | >WP_002657207.1 aminoacyl-tRNA hydrolase [Borreliella burgdorferi]  MGLLILGLGNPGLEFSLTRHNVGFSLLDKIVSKNGLFLKRKKKYEYSELKMISGRVILVKPLTYMNLSGSLFPLIFSDFY  MCIKNLLVVLDNVDLPLGKCRLKERGGVSTHNGLRSISSVLGSSNYSRLYIGVGSNLMRDIKSFVLSRFCKDEMDRLEKL  YDFLSDELIDISEANFKNKVQKINSSNF | Virulent | non | - | - |
|  | 61 | >WP_002657210.1 ATP-dependent zinc metalloprotease FtsH [Borreliella burgdorferi]  MNGNNNMNNNGKSNNKKKNKNWILGLVVVFLISAIFMSYFIRGGKSYKNVPYSTFQSYLDNGLVESVVIIDKNLIQFVVK  GSNFAKSYFSTSIPYLDINLLSELKNKKVELSSGKSQASLIGVLLQTLPWILFFIFFFFIFRQTQGGGGKVFTFGKSNAQ  KYEAGKNKITFKDVAGQEEVKQELREVVEFLKNPKKFEKIGAKIPKGVLLVGSPGTGKTLLAKAVAGEAGVSFFHMSGSD  FVEMFVGVGASRVRDLFDNARKNSPCIIFIDELDAVGRSRGAGLGGGHDEREQTLNQLLVEMDGFGTHTNVIVMAATNRP  DVLDSALLRPGRFDRQVTVSLPDIKEREAILNIHSLKTKLSKDINLQVIARATPGASGADLANLINEGALIAARNNQDEI  LMKDMEEARDKILMGVAKKSMTITDRQKLETAYHEAGHALLHYYLEHADPLHKVTIIPRGRALGVAFSLPREDRLSINKH  QILDKIKICYGGYASEQINLGVTTAGVQNDLMQATSLAKKMVTEWGMGEEVGPIFLVDDEAPIFLPKEFSKAKAYSENTA  DKVDREVKRILEECLKEASDILLKHKDQLVKLAKELVLKETLTDKEVRELLGFEANKDEYDLFSSDSTTKEVKGEDVKG | Non-Virulent | - | - | - |
|  | 62 | >WP_002657235.1 LptF/LptG family permease [Borreliella burgdorferi]  MKILKNSYESYIIAEFFKYFLITFLFFFFVFFINQILFFMRILLQNYVPFFKAFIFIIYSLPMVIALSPPFASLISVILT  IHKFKLHNEILAFRSIGISIFDLIVPFFKLGIVIAFVSFISNDILLPLGSIGRLKIFNEIKEEVPHLVLKPYSSKQYGDL  IFVSGEKSENGYKNVTFFDNTGLKGFDRIFMAKNLDIRKENFQVYFILNDVLSIALTDSESGFYDYFYADKMKYSIDQVT  FSDSFLLNYVTPSQMSMRDVIKLIKKQNNLIADSNIKNNLEGDFLSLNFSNLYLNYLYNQNYYVDESYVFENLNYMYNLN  LNFKPYQDRSMKQNLALFNLEFYQKISLPLSVLFFIFLAFSMGMYSNRKYSIILELVISIIVCVFYWVMFIGGKVYTVQY  APSPIIVTILPNLILIIAGAILFLRLLKK | Virulent | **0.64** | **Allergen** | **-** |
|  | 63 | >WP_002657239.1 murein biosynthesis integral membrane protein MurJ [Borreliella burgdorferi]  MNKYVVSTILVMISTFFSRIMGFVKIKIFSYYFGANLDADIFNYVFNIPNNLRKILSEGAMTSAFLPEFTHEKNKSHEKA  VSFFRTVITFNIISIGLIVLVMIIFAKPIMYFISYYRGENLIFASSVFGYLVLYILLISLSSIFVSVLNSYKIFFIPSFS  PIMLSFGIILSIFLFYGRFGIYSAVIGVILGGVLQFLIPFANCLMIGFAWKPTFYFREKVFLNFLTRWLRMIFGFSISII  TQQISFALASTLEIGSVSILSNAVVYYQLPVGIFYISIATVIFPKMAEHAVLGNNIKLNALLVDGIKILLLIFIPVSFLM  FIWSDYILNLFLMGGKFSIYDTQKTASVLKCFLLGLLFYSMFGFFQKYYFSIRDAKTPFYLSVLFSILDIALSVFGINYY  GLNALALAQSISFMICVIVFYFIILKRGVKIDLIEILFVLLKSIITLFPLYAIYFFFEKFQWDVGFSFKNLYFLMAAGIV  SIFVLFICYSVLGINKLFRYIRRDAL | Virulent | **0.64** | **Non allergen** | **58.0** |
|  | 64 | >WP_002657248.1 sodium/pantothenate symporter [Borreliella burgdorferi]  MLLNKYFLANRNINFIVMALLFSSSYISASSFISGPSAVYKYGLSFILLATIQIPTTLIVFIIVGQRLNRESKKINAINI  IDYIRYRYESDVLALMSGFVLIFFSMFLISAQLIGGAKLIEVFWGIDYVVGLTFFAFLVFIYVFFGGFKAVAYTDLIQGF  LMLVSSVILFSKMLDLGGGINNLFKTATSSLDKSLLLPSNADLKPQYIISFWILIGIGILGQPQIINNFIAFKDGNAIKF  SLPISTFIISFLIVLMHLIGFFAIILFPDLSPNDKVVLNVALKVLNPFSCVMFFIGLLSAIMSTVDSNLLLITSVLIKSI  FIYKEDLKEDVKIGRVIMISNIFFILIILIFSLFPPNFLFFINIFAFGALEVSFFPIIVFGLYLNFVSKIAAFASMFLGL  IFYLSIVFFGLNIWFFHPVFPSFFVSIFTFLVVNFFCKKNSKVC | Non-Virulent | - | - | - |
|  | 65 | >WP_002657298.1 YfcC family protein [Borreliella burgdorferi]  MIKMPSSFTIIFSLIVFVTILTYVIPAGKFDKEFKQMGDGSKREIIVAGTYQYVDRGSRGFLHPIMTILTAMSKGMEHAV  EVIVFVLIVGGAYGIIMKTGAIDVGIYFLIKKLGHKDKLLIPLLMFIFSIGGTVTGMSEETLPFYFVMIPLIVALGYDSL  VGAAIIALGAGVGTMASTVNPFATGIASAIASISLQDGFYFRIVLYFVSVLAAITYVCVYASKIKKDPSKSLVYSQKDEH  YQYFVKKDGLSTGDNAQNALEFTFAHKLVLLLFGFMILILIFSIVNLGWWMQEMTMLYLGVAIISAFICKLGETEMWDAF  VKGSESLLTAALVIGLARGVMIVCDDGLITDTMLNAATNFLYNLPRPIFIILNEIIQIFIGFVVPSSSGHASLTMPIMAP  LADFLSIPRASVVIAMQTASGLINLITPTSGVIMAVLGISRLSYGTWFKFVLPLFMIEFFISILVIIANIYLSF | Non-Virulent | - | - | - |
|  | 66 | >WP_002657319.1 cation transporter, partial [Borreliella burgdorferi]  MVKVISLKNIHKFAYLKLDPLKKEDIYIVYIETNSKLIANLKAKTKVDQIEIINFYIDDDFKSEGIERIMISNLIHYGKK  NKFKTISCQIAEIQEELLSLGFEYNDSKYKKELASEIEEDKFVMGIGIISIFTEVASISSKLTVGILFNSFALIADAFHV  MADFVLSTITYFSLKITSKPETIHYPYGHKLMESLIAFIMGIIILMTGFTLFLNTTGLNKFITLGGESGFNLHIHQNKNK  NDTIYEHDHYHSHDHDHDHNHDHNEEDKKNILEIFSNKSLKKSLWIPLTPFIFFIVKIIEYLTKFQIGKRYNNQLLLALA  SADKNCIFSHGGITLSLLLATYMWSGFDKIMSIFIGFIIIKEGLNVIINNANNLLSKQNIDLKRSVKDTLKNSNINFKTL  NFHNQGNKL | Virulent | non | - | - |
|  | 67 | >WP_002657338.1 fructose-specific PTS transporter subunit EIIC [Borreliella burgdorferi]  MQNLFSKNLIVLNYNATSKEDVIRKMASMFNENGYLNDMEAFIKEIKKREETNGTGIEEHIAMPHAKGNFIKKHGIAILR  VVGNGFDFNSSDQKLSKLFFMMALPEETPSNAHIKAISYLSNTFSNNLLRHELMSTNNEDRFLEIILNNDNINESNNLNT  KKDFILAVTACPVGIAHTYMAAESLKKAALELNINIKVETNGSSGTENPITEEEIKKAKGVIIASGKTIDKERFSGKPLI  EVGVKDGIHKAKELIQTILKNEAPIYKKSNTNKTTETLQKQNKKTGIYKHLMNGVSFMLPFVVSGGIIIAISFMFGIKAF  DINDPSYNKIADILMQIGGGSAFALMIPILAGYISFSIAERPGLAPGMITGLMMNNGNAGFLGGILAGFISGYVTLTVKK  ISDKIIPSNLRGINPVLTYPFLSVIISGILIYGMLSPISVINESITNMLNQLSGTNMAILGALLGGMMAIDMGGPVNKAA  YAFGIAMITAKNYIPHASIMAGGMIPPIGIALATSLFKNRFSKEERESGKVCYFLGACFITEGVIPFAAADPLRVIPACI  LGSSVGGFISALFKVEVIAPHGGIFILPIVVNPLMWITSILVGSIITAVLIGILKKEYKNIND | Non-Virulent | - | - | - |
|  | 68 | >WP_002657347.1 Na+/H+ antiporter NhaC family protein [Borreliella burgdorferi]  MENIEVRGQPNFFGLIPFFVFIIIYLGTGIYLGVIGVEMAFYQLPASVAMFFASIVCFLVFKGKFSDKIHIFIKGAAQYD  IILMCLIFMLSGAFSSLCKEIGCVETVANLGIKYINPNWIVSGIFFVTCFLSFSAGTSVGSIVAIAPIAFNIAVKSGINP  NLIAASVMCGAMFGDNLSLISDTTIVSSRTQGSSILDVFISSSFYAFPSAMLTFFSFFFLSENLSNATNFLHESSIDLVK  TVPYLMIIFFSLAGMNVFIVLFLGILSICLISVLYGNLYFLDVMKNINKGFLNMADLIFLSILTGGVSFAVIHNGGFKWL  LIKLKSLIRGKSSAEFSIGAFVSIVDVFLANNTIAILICGKVAKKIAFENNISVQRSASILDMFSCIFQGIIPYGAQMII  LVNFSNGLVSPISILPFLVYFGFLLFFVILSILGLDIKKVFLFFFKK | Non-Virulent | - |  |  |
|  | 69 | >WP_002657349.1 Na+/H+ antiporter NhaC family protein [Borreliella burgdorferi]  MERDLKLERETNLVPNFWGLMPFFLFIGIYIGTGLVLYFNGVERAFYQMPPVVAMFIAIVLTFIIFRGSFLAKMNKFIEG  CAQQDVIFISLIFMLSGAFSAVCKEIGSVDAVVNIGLKYVPLNLIVCGIFLITLFLSFSTGSFMGTIVAVAPIALELADK  VNIPLPLIAGAILSAGAFGDNMSLISDTPIISSHTQKVNIVDVFKNGAFYTFPAAILASIAFAFLGSYYCKIDSFIIEPG  EINFFKIIPYIFVMVFAISGFDVFLALFLGIVVAGIIGIYYSDITFLLLAKKINEGFLGLGEMFILVISTGGISYMTIKY  GGFDWLLLKLQKMSKSKRTSEFVIVVLVGILTMFLANNGLAILMSGSVVRSITKENNLNSKRIAALLCMSSCFFLSILPH  SMHVIALVDFTKGKLSPFDIFPFLIYQGFLILLIALSIIGLDIKLIFKSFLKIVTKLKSFKF | Non-Virulent | - |  |  |
|  | 70 | >WP_002657353.1 ABC transporter permease [Borreliella burgdorferi]  MFRAFKNIFLFLILSFIYLPIIILIIYSFNSGDSGFIWQGFSLKWYKEIFVSSQIKSAIFNTILIAIISSLTSVVIGIIG  AYAIYKSENKKLKTILLSVNKITIINPDIVTGISLMTFYSAIKMQLGFSTMLISHIIFSTPYVVIIILPKLYSLPKNIID  AAKDLGASEIQIFFNIIYPEIVGSIATGALIAFTLSIDDFLISFFTTGQGFNNLSILINSLTKRGIKPVINAISAILFFT  ILSLLFIINKFIGIKKLTTDAEL | Virulent | non |  |  |
|  | 71 | >WP_002657355.1 ABC transporter permease [Borreliella burgdorferi]  MKKLILIIYSIFLLTFSILPLLIIILLGFLNEKNEFTIYNFIGLLNPSYLNIFSRSLKLATIATIFCILIGYPAAWLISL  SKKSAQNKLIIMIILPMWINTLLRTYAWMRILGKNGFINNLFEKIGIGTLDLLYNEQAVTIGMIYNFLPFMILPIYTGLL  KIKPEYIEASQDLGARMWQILLYIKIPLTLSYLATGIIMVFIPSITVFIISDLLGGSKQILIGNLISKQFLFIEDWNTGA  AISFILMLVILIFNLIIIKLMRKNNGE | Non-Virulent | - |  |  |
|  | 72 | >WP_002657363.1 preprotein translocase subunit YajC [Borreliella burgdorferi]  MYSMGGFVFLLQEFSGNSSFLRSLLVFVPVIAIFWFLVISPQRKEEKNKKEMIKNLKKGDKVLTIGGIFGVVKKLGDTDV  ILELSPNNEAVFIKNSIDKVLSEKK | Virulent | non |  |  |
|  | 73 | >WP_002657365.1 protein translocase subunit SecF [Borreliella burgdorferi]  MQRVINFSKYGSNVLIVSAVLILVGLIYTFFYHGGYNWGIDFSSRVNINLSIEKSNIKENEIKEIFSPIYKTLDVNSIFS  PDQNKSEFSIMVKSDVIDYAFKTEVQKTILDKLKETFDANIEVLDSYFIDSSFSSTLRIRSIFLVLGTFILILIYITLRF  KLSYAIASILSILHDIFFIVAFLGVFRIEINSYIIVAILTIIGYSLNDTIIIFDRIRDNVKRLTDNTFLNVLNISISQTL  SRTVLTSVTTFVAVFSIYVFTEGSIKDFSLVFMVGVIVGTYSSVFIASPILLNLYKKIK | Non-Virulent | - |  |  |
|  | 74 | >WP_002657380.1 GTPase Era [Borreliella burgdorferi]  MKSGFAAILGRPSTGKSTLLNSICGHKISIISPIPQTTRNNIKGIFTDDRGQIIFIDTPGFHLSKKKFNIAMMKNIHSSI  GEVELILYIIDIQDKPGEEENKMLEIIKNSKIKFLVILNKIDLKNTKIKEITQFLKEKGIEDSNIIKISAEKKINTEELK  NKIYENFSEGPLYYPQEYYTDQEINFRISEIIREKAIENLKEELPYSLYVDIDTLENKKGSLFIRANIFVANESQKGIIV  GKNGKEIKSIGERARKTIAKIFETKCNLFLQVKLKKNWNKEDKLIKRLIN | Virulent | non |  |  |
|  | 75 | >WP_002657405.1 hypothetical protein [Borreliella burgdorferi]  MVKKFSIFLKAIIIFSIFELLIEELSIILFLPYKIRFALIFLGFLFDTIFIFIFLYKITKAYLSQRLEIYVRNNLFFDII  HCLIPLAFYSSYQLKNIIVAHETILNPIMLSLFKLRFLRLLRFNDLIIEIYYNSKEKNLILIAFARTFSMSLLIPFTFFI  IISSSKIVNSIPEKQEFNIIKNISIINEKAYIKEKYPFILIIKEKDDIIYSKSDEIFVYYSPSEYRVIEMEKTKFYIDKY  LQRKSDSILGIFLFTLFASFTIFLMNFYKFFKASFLNPIILMTKILQDPLEYRKIQIPFTLSEEKVYELAKSFNNLLLKE  KLNSKRKSKIPLEIEKVKKIINKNQEIK | Virulent | **0.53** | **Allergen** | **-** |
|  | 76 | >WP_002657410.1 ABC transporter ATP-binding protein [Borreliella burgdorferi]  MEEYVLVLENITKKYGDFVANDNVSIKFKAGEVHAILGENGAGKTTLMKTIYGIHQVNSGRIILKGQEINFKDSSEAIRN  GIGMVFQHFMLIPQFTAVQNIILGYENSKFGFLDYKQARKKISSLSEKYGLKIDLEKRVEDLSVGMEQKIEILKVLYRNA  DIIIFDEPTAVLAPSEVDDFINILKVLAQEGHTVILITHKIKEIRSIAKKCTIMRLGKVVKTVNIADIDDKDLTKLMIGK  EVALRSSKIKFENHFNILEIKNLSVKDERGVLKVKDVNLDLRNGEILGISGIEGSGQEDLVDAILGLKSIFKGDILKKNS  SGNLESLKGLTIKQRIDKKIGNIPSDRQKHGLILEFNVMQNIGLKSFDNPDYLRLKTIHLKSNFDLKFNFFNFIKRQFNK  FKKQFVGFDLNILRKLSNQLVSHFDIRPRDILSKVKHLSGGNQQKVIVAREISLEPDILLAIQPTRGLDVGAVENIYKRI  IEQRDAGRSVLLVSLELDELVNVCDRIAVMHGGRIVGILEDNFDIDVIGKMMIGLS | Virulent | 0.52 | Allergen | - |
|  | 77 | >WP_002657415.1 methyl-accepting chemotaxis protein [Borreliella burgdorferi]  MLLKLKYRFVGFLLLFLIFILLLFSTIFNFVLCGYLEDYYKQLTRAQVRRAAFSLQSFLDTLHVIINGAASNLALETISE  FAMSENRGKDFSESELIDLRKNPKFVIDSVKVSKKYRQYLYNFMANLKNDTLFEEFAFFDFEGRIIVSTRHENNMDFGHS  EANTNYFKKAVEDYRQNQLKFIGWYSNLSEGISAEVAIRSKQSEKKAFAIIVPVYSPEDKLVCGYLAGYLLNDIVADSFD  RFRFGFYKRGNFIYVDPNNIAVNPFEEYNETSRVSSKFLNVLKDVFSKPPFPSNIASEVSVYTIDRILLSEMGEDCYYAM  LPISSKLGEKSGVLIARLPYKDIYGVISSLRFQYILYSVLGIIALSIVLSIRIDRIISFRLNAIRVLVQDMVKGNLDKDY  ALDDDENTLDELGMLSLQVVKMKKAISVAISSVLRNISYVNKASLEVASSSQNLSSSALQQASALEEMSANVEQIASGVN  MSANNSYETEQIALKTNENSQIGGRAVEESVIAMQDIVEKVSVIEEIARKTNLLALNAAIEAARAGDEGKGFAVVASEIR  KLADLSKISALEIGELVEDNSKVATEAGVIFKEMLPEIEETANLVKKISEGSSKQSDQIAQFKMALDQVGEVVQSSASSS  EQLSSMSDKMLEKSKELRKSVLFFKIKDSKIENPENDDYDFRLIDCPENSFKDENQNLKSNGISTSNASGHNNYSLDIES  ESSVRTINKRVDPKKAIDIADKDLNFDDDFSEF | Non-Virulent | - |  |  |
|  | 78 | >WP_002657417.1 methyl-accepting chemotaxis protein [Borreliella burgdorferi]  MKLKARMLLLVLILIAFFISILFFAFGMLINSKLVDQQFNLMINLIESIKSSFNLYISSMEEKVRVSSMYFNSAEKFNEA  SKIKSKRLSFISDQSEILIQTGSNMMVTDKEGKIVFTTAVKDNSDFGKSIGDREYFTKLKESNSIVYNSFVMLADPGSIE  ESLLKDISKIKNKKGQIPYILIGMPLRDFETDNIFGYFMFLYSMDYIYRSFRGINFGILSSGRALAYDTTGRLLVHHVVL  PGDILTDISASYSNIIKKTSEDLLQKNKEISTVYYYDPKSNKKYVGISQKVLLNLSNNKFILLMRTSEDDFYYMSRATTI  ILAISFVFTLLMLAIATLYLVKKLSSSLNKILEYSERLASGNFTADINFGKWDTVELYSLYEGLEQLRTNFSSVAKGVIE  NLDYLYENAIQIANASQNLSSGAVEQASTLEQMTANIEQISQGVSENTENAATTEKIAVNTNERTKEGHKSVVKAIEAMT  VITEKIGIIDEITRQTNLLALNASIEAARVGEKGKGFEVVAAEVRKLADQSKESAREIIDIANRSLTVASRAGENFEQIV  PGMEQTARLVKNISNESYKQSVQIEQFKNAIEQVSQLVQTTASSSEELSAMSEKMLESVKDLKESVDYFKIEK | Virulent | non |  |  |
|  | 79 | >WP_002657434.1 signal recognition particle protein [Borreliella burgdorferi]  MLESLGSNFRNFINYLSGKSTINDKNIAEAIEIIKNSLVDADVNLRVIRRFLNSIIEESKGVKVLRGIDPKSQFIKIVND  NLVKFLGGKNYELSLHPANKQSYILMLGLQGSGKTTTCAKLSLKLKKENRKVLLVAADTFRAAAVEQLKILGGQVGVPVF  SIEGEKDPIKIVKASMKFAESNFFDSVIVDTRGRLEIESLLVEEIKKIKGILRPAETILVVDSMMGQVAVNIAKEFNENV  GLTGAIFSKFDSDTRGGAVLSFKSICAVPIKFIGVGEKIEDLDSFYPERIASRILGMGDVVSLVEKVQSVVDKEEAIKLE  EKINKASFNFEDYLSQFRRIRQVGGFSNFVSFLPGVSKSMMNSNNLNEESFNKEEAIILSMTKKERINPVILNNPSRKKR  IAMGSGTTVFDVNKLIKKFSQTTLIMKKMKNKDFQNKIASLLGK | Virulent | non |  |  |
|  | 80 | >WP_002657457.1 penicillin-binding protein 2 [Borreliella burgdorferi]  MGVITNFRYKFGIFFLIVIMVLYLAILFQMQIGKHLFYDREANVFLSRLEKINASRGEILDSNSNVLANNLTMFILKISL  QQYYNMPATTRIEMIDFLSSTLDIDKSIILSKLQEPGGYLKDVEIIELTPKMLFKISEKKFYYPALLWTYSFKRNYLVDD  SYSHSIGYVGQINQRELRTFYNVSGYDNTSTIGKLGVEQVYDNYIRGQEGLIKYKVDSKERRIDDGSIIKNMVPGNDVVL  NINKDIQDLAKNALGKRYGSVVVLKPSTGAVLALHNYPYYSMKDVYNKDNKEDYSFLNKAIQSVYPPASIFKLVVAAAIL  EERVIDKDRKIYCPGYFKVGNRIFHCWKPGGHGYVNLEEAIAHSSNVYFYTLGLKYLGVDRIRKYAKEFGFGEKTGIDLP  NEVAGLLPSPEWKEKTFNQPWVGGDTVNFSIGQGFLNATPMQIVNMVAMIANEGVVYKPRIVNKILKGGTNKVVLENKPE  ILRKTNLISKNTFKLLKKYMRSVITYGTARYAVLTKAVKVGGKTGTGQTGIDGFENSSFIGLAPYNGSADNQIIVFSLVE  AKSNVDWWPAKSTDLIMQGIFANQSYEDILKGYRPWYIR | Non-Virulent | non |  |  |
|  | 81 | >WP_002657459.1 rod shape-determining protein RodA [Borreliella burgdorferi]  MVFRKNYDYLALISLLIVSFVGILLIYSSDYNISGSLTKNEYIKQTFWVIIGFFLIFIVGKYDLKFVYSMVYPLYFLLIL  ALIFTAFFGMTVNGARSWIGIWKLGGQPSEFGKVIIILTLSKFYTEKKGYNEFFTFITAFLLIFPSVILILLQPDFGTAI  VYLTIFIFISFFAGIDLHYVLAFALIGFFSFVFAILPVWYEYKVNMGNVFYLIFSNPFYFRVIMGVLLLILLISVLGFFI  SKYGLSIKIIYFYVFFASSILLVSIVFSKVLSKLMKTYQIKRFLVFLDPAIDAKGAGWNLNQVKIAIGSGGLSGKGFLKG  PYTHANYVPSQSTDFIFSILAEEFGFLGVSTILILFFFLFFKFLIIMNKSQDRYMALVISGILGLLFFHTSFNVGMSLGV  LPITGIPFPFLSYGGSSTITFFLAMSFYFNIESIVAMD | Non-Virulent | - |  |  |
|  | 82 | >WP_002657463.1 CDP-diacylglycerol--glycerol-3-phosphate 3-phosphatidyltransferase [Borreliella burgdorferi]  MNNLIKVITPNKITLVRIALSFIILILFFLENVFFSYLFFGIIWFLIIFNEFTDFIDGYLARKYGLVSNVGKILDPYADV  LQHLTYFVFFFYKGITPYYFFVIFIYREISIGFVRNLIIQFNVVQQANFLGKLKSLLYAVCTFASLLFYTLNQLNFTESV  QNFISYILTFEFKFLFIVQMTYVCAAFFAILSFLEYVLIFLNVKKYENK | Virulent | **0.57** | **Allergen** |  |
|  | 83 | >WP_002657469.1 TrkH family potassium uptake protein [Borreliella burgdorferi]  MLKFEFSDRFLLFSYFVLIMFIGSLLLMLPISWEGDGKLAYIDALFTAVSAVSITGLTTVKMEGFSTFGFILIMLLIQLG  GLGFISITTFYLLIPKKKMNLTDARIIKQYSLSNIEYNPIRILKSILFITFSIEMIGLILILICFKLRGVNISFLEALFT  TISAFCNAGFSMHSESIYAWRDVPEAIVVVSILIICGGLGFMVYRDVNNTIKNKKKLSLHAKIVFSLSFFLIIIGAILFF  FTEMHKLKAGYSMSTLIFNSIFYSISTRTAGFNYLDNSLISGRTQIISLPFMFIGGAPGSTAGGIKITTFFLIVLAVVKN  QNGNGYIIGSYKVSIDSIRFALLFFARAIFILSFSFFMLLFFEGGSGNWKVIDLGYEVFSAFGTVGLSVGVTQDLSFWGK  VIIIFTMFAGRIGLFSMAVFVSRKSRFEEFTRPRQDILVG | Non-Virulent | - |  |  |
|  | 84 | >WP_002657472.1 MinD/ParA family protein [Borreliella burgdorferi]  MTKIIPVASGKGGVGKTSFVANVGYKLSSLGKTVILVDLDLGGSNLHTCLGVKNKGVGIGSFINKKSKSFSDLVCKTSYD  KLYLIPGDALYTGTANLPFSIKKKIIESIQKDLIADFVFLDLGSGTSYNTIDFYLASYSGVIVTIPETPSILNAYSFLKN  ALYRLLYLGFPQKSPERDYIGNFFKDKIEGTNLGFKDLVVGIELISLSSSLKVKRMMNNFYPRVVLNRIETSEEIAMCEN  LINVVKNNINIPIEFIGFVPFAKSFREAINNRVPFIDFEKNSKLNKYFEFIAGNLIKSPIEGSPYIYDDIYDMIKDQSRF  IRK | Virulent | non |  |  |
|  | 85 | >WP_002657477.1 L-cystine transporter [Borreliella burgdorferi]  MDKISILYTLINIIIMLILISIVYLCKRKNVSFTKRVFIALAIGIVFGMTIQYFYGTNSEITNETINWISILGDGYVRLL  KMIIIPLIITSIISAIIKLTNSKDVGKMSLLVILTLVFTAGIAAIIGILTALALGLTAEGLQAGTIEILQSEKLQKGLEI  LNQTTITKKITDLIPQNIFEDFAGLRKNSTIGVVIFSAIIGIAALKTSIKKPESIEFFKKIILTLQDIILGVVTLILKLT  PYAILALMTKITATSEIKSIIKLGEFVIASYIAIGLTFLMHMTLIAINKLNPITFIKKIFPALSFAFISRSSAATIPINI  EIQTKNLGVSEGIANLSSSFGTSIGQNGCAALHPAMLAIMIAPTQGINPTDISFILTLIGLIIITSFGAAGAGGGATTAS  LMVLSAMNFPVGLVGLVISVEPIIDMGRTAVNVGGSMLAGVISAKQLKQFNHNIYNQKELVNK | Virulent | non |  |  |
|  | 86 | >WP_002657480.1 PBP1A family penicillin-binding protein [Borreliella burgdorferi]  MKLNSPNLKKINTHKLLIYLTYFAVSFSIITLSLAVSKTINIQKDKNFGYVNPAVPSRLLDINGKQITQFISDENRELMP  LRKMPDNLINTLLIREDIGFFSHRGFSLIGIFRAAFNIVLGRYFSGGSTLTQQLAKLLYTNQARRSILRKLHEIWWAIQL  EKKLSKYEILEKYLNKVYFGNGNYGIVAASKFFFGKSVNKINTAESVMMIIQLPNAKLYSPLYNPEFSKKIQRAVLNQVV  SNGIVKAEIAEKEFNEYWQNYDWTRMADTSAISNKKDQAPYFSEYIRQKILKYLPDGANIYKDGYSIYSTLDLEAQKYAD  KVTNDMINKARTMHNLNRSSETIIINSEIVPVVDAISDLLGIKNLRINGRQYKKLRKRKFYEDNIDLIASFGAILGIDKI  DKATKEYIIKNKLTPKLIAQPEGAMIAIDTTSGAIRAMVGGSGHTKDNEFNRATQAKVQPGSAFKALYFAAAIDLKKITA  ATMFSDSPVAFLNKNGEVYAPGNYGGKWRGNVLTRQALALSLNIPALRILDRLGFDSAISYSSKLLGITDPKEIEKTFPK  VYPLALGVISVSPIQMARAFAILGNSGSEIEPYGIRYIEDRAGRIITNEEASILAKIKNKEHQTQIVSPQTAYIITDMMK  STIQYGTLANQRYTNLKNFKSDIAGKSGTTQNWADGWAIGYSPYITTAFWVGFDKKGYSLGISGTGTGLAGPSWGEFMAE  YHKNLPKKVFVKPAGIISIPVQAETGLLPEEIADEKIINELFISGTQPVEKSKYYENKQEFKNTIEFNIYGIDEINNNDE  INFDTPEFEYLDNNLESFNNNSNNDNNLESFNNNNNDLESINDNEENKNEDEIEMNIEEPLNEIENKNPQQDLVNNNNNQ  EMLIENTKEIKDEVIVNETNIETQSTKELNSNNNENEKINNKDVNGEDIQLD | Virulent | non |  |  |
|  | 87 | >WP_002657522.1 DUF368 domain-containing protein [Borreliella burgdorferi]  MLNIYIKGILLGIANIIPGVSGGTLALILKIYYKIINSISEILKLTEIKKNLMFLTILATGMLTSILLTAKIFKTYAFDN  GIIEALLIVFFIGLAFGNILTLKTEISIKEINSNTKILNNLLFFIGMSIIVLFLILKESNIQLQSTIPKDKNSIKYYLLL  ISSGTISGASMILPGISGSAMLLLLGFYKEIILIVSEFNIILITIFAAAATMGIITSILIIKKIIDKHLNNFIYLSKGLI  FGSILQMILIVLKLNFKIGFTSFTSLGTSFILGIFINKKLAEKYK | Virulent | non |  |  |
|  | 88 | >WP_002657528.1 ATP-binding protein [Borreliella burgdorferi]  MNNFFKKALTKLNKLSNEQKTKFIEQIYKKIEIYDGIFASINEGIIVLDKQNNIIYANKILYQILALTSKSKIEILDDIQ  IPTLINLIKELVRTEDKIIGLEVPISNGIYIKISFMPYVKEKKLEGNIILIEDIKEKKKKEELFRRVEALASFTRHARNI  AHEIKNPLGAIDINLQLLKKEIEKQKMKNGKAENYFKVIKEEINRVDKIVTEFLLTVRPIKINLQEKDIKQVIGSVCELL  NPGLENKHIKLLLNLNKISNILIDEKLLKQVIINIVKNAEEALLETKKEIKKIEIFLFEKDNKIHINIKDNGNGIKDGVK  EEIFKPQFSTKEKGSGIGLTISYKIIKELGGEIFVESKEGKGTIFTITLPKLNKKNILIEGY | Virulent | non |  |  |
|  | 89 | >WP_002657532.1 undecaprenyldiphospho-muramoylpentapeptide beta-N-acetylglucosaminyltransferase [Borreliella burgdorferi]  MSNKKIIFFTGGGTGGHVFPGISIIQKLKEFDNEIEFFWIGKKNSIEEKLIKEQDNIKFISIPCGKLRRYFSFKNFTDFF  KVILGIIKSFYVLKKYKPQLIYATGGFVSTPAIIASSLLKIKSITHEMDLDPGLATKINSKFANNIHISFKESEKYFKNY  KNIIYTGSPIRREFLNPDPKIIKQLTQNTNKPIISILGGSLGANALNNLALCIKKDAEIYFIHQSGKNLNDLSEKNYLRR  QFFNAEEMASIVKFSNLIISRAGAGAIKEFANAGACAILIPFKKGSRGDQIKNAKLLTNQNACIYIDEDEILNINILKII  KKTLKDREKINSLKENIKKFNNKHSSTLIAKLLIKDIKETKSK | Virulent | non |  |  |
|  | 90 | >WP_002657576.1 SH3 domain-containing protein [Borreliella burgdorferi]  MVIFFIYFFSIARLYSLTGIDFVKNIKVLKGDKFIQIVRLNNPLQDIDISLLKVEINKEVQSNSNVLSISRTTDNNFSFI  EIRVEYLFESLGFIKIPPLKVIYKGDFYISSEVEVSVLRADEINSFGLPVDLYWDLDKKEIYEYQSIGLILRSNWLSDSN  SNEMSGSLPAIKDAMVEKMPIFGDIKYRTFHNKEILDVPFYNFVLTPLKGSKNVLIPSFSFNIGSGLVRNTPELLLKVKP  IPGEVKSLAVGTFRVDYETPTYSTIEQDIFTILIKITGQGNFPHFYFPEIETYNSKILNKKKNYSFKPSKNGYKGSISQI  YTVKPDTKGSVFLNIGDFNYLNPDNDTVYTLKGKKLKYEYSGEFNSINRAQNNVDYDFKLLSYADILNYKNKTFLFFVSY  YYLLLIPGFLLSLIILINYKKFFAASSFGLVILILAVGISLNAVNDGLLSEKNVNDLIENYNTKNYDTALIKIDNILKKY  PNYSGLWLNRALVLSKMDRDFDAIYSAYKAFLASPNNETSYKVIDLIEAKNGVTDNVRNNSFIFSNIFFIISLFLINFLV  VSISYRFLAKNLKKIIIFLLFSAVCFTIFETYYFYYEQQSEVGIIKGDLVSLYKVPDNFSRSWRFLKGNASVYILDSKDD  FVLIETSYGLQGWIHKNFVVSLKDDLI | Virulent | 0.52 | Allergen | - |
|  | 91 | >WP_002657582.1 VWA domain-containing protein [Borreliella burgdorferi]  MLTFNEPLYLFLLVIFPLIIYFNHFLKNRGGKIKFPISLYGNFNSLKLKDYRLNLMYFFTYSFLYLAAMVMVFALAGPSV  SKKKMIHLSAGADIVIVLDISPSMGAVEFSSKNRLEFSKELIRSFISQRENDNIGLVAFAKDASIVVPITTDREFFNKKL  DDIYIMDLGNGSALGLGISIALSHLKHSEALKRSIVVLTDGVVNSDEIYKDQVINLAQGLNVKIYSIGIGSSEEFSVEFK  LRSGKFYQGSFKEVYDPSMLVEISNKTGGLFYSVNDDFSFQFAIQDFSKKENLERKIKIAVDNKDIYKEFLVLAFCLLLV  YFIFSKIFLKEIL | Virulent | 0.73 | Allergen | - |
|  | 92 | >WP_002657611.1 hypothetical protein [Borreliella burgdorferi]  MTINILLLLLLLSSTGPTIVPNKMEKNAFNSSNKITSIETEKNIKTHIRKIHQLNERQEKIINTFNYIKKYFNTNEVKYM  EYSLQEIGFIGYSQKIIHSKIKGKNANTYNIITPIKIQKNLKNNLAIGIAIELLNKLKNTNPENNINFFFVEDDSLEQNT  IISSRILLSNKYLGKNTNTIYLMLNENKIKNKIYLENESFITNSKTNLGFLKAIIKTFEKNKLDFNTAKITEKKSNNLYN  LYRDESIPLLIINNNLNPILTQNKNTIFEIYKSIEETLTNEYKSKNTDETHYIVIDTPFKKIIINEIALIALIYICYNLI  IIVFIRKFKEAGIVMKKVKNNYYKLVRLFFTLFLSTYISLLLTNKIFAGYENTAIYSITNPKYLITFFATLLIHNLLSLF  TYNFTIYLNYRQLKYLAISTSIIEPIILMFIKIEFILIIIIKSILLLIKPNKNTIIRKIVILIIWIINFVLITLIQNTTS  IKNTLTLSYIISTCLFSSIFINISEHLKSKLPKIKQNLKKAENLGLVTFFLITTIIISSNIDKKEHEIQIEQTVSFPEKI  NKIKIEYPKDKKHPIQIISNDFNLTLQANEKILKTNIEIDDELMNIDLKKIDIAERAVYSIDLVTQKIANQIELHFKNAS  KLIIYQSNTPYKILANKIIFTLKNINSKKTTIAFTLKSQDDIAYEAFANIYIKKNQIKIYNKTNNTEEKNIKINYSYKIK  YSGILPKAEKYKNLEYFKLKDDKEIENLKNLKLN | Virulent | **0.5198** | **Allergen** |  |
|  | 93 | >WP_002657642.1 phosphate ABC transporter permease PstA [Borreliella burgdorferi]  MYNSLFYFKKKQTLFLNKTQSFVPFKTEDGKEIQIAFIVNKNINTDKISIEDIHNIYNNKISHWGSISDQGIDIIPIASS  IDSISNKAILKTLIKNNVFNKRYIKIEPSTKKILQTINSTIGSVGYLTKKEFESLDFKLYPNIKALKIKTMSVLISKKTL  TKNENEIINTLGVDEVEKLIKGKEKWVNLISKDIKLKIIKYLDQEENIIQTIEKTEGALAIVPWHYFQNFKAPFIKMHYV  DKSSPLNLNFILSIPRDSGAYGGISYLILNTFYVILLTTAISICIGIGTGIMLAEYTSSKIFYKILSMSVDILSSIPAII  FGLFGLIFFVPILGIGILSGAITSSLMILPMIVKTTEEAFKTIPKSYKYASFALGANKTETIIKVMVPAAIPGILTGIVL  AIGRALGETAVLLFTMGTNLGLATNLNEPSRTLTVHLLMLFQEGHLDKGFGTASILVIMVLIINLTSKFLINKLYRIK | Virulent | non |  |  |
|  | 94 | >WP_002657644.1 phosphate ABC transporter ATP-binding protein PstB [Borreliella burgdorferi]  MIKEKDTPKNEVIIETENLNLFYTDFKALNKINIKILKNSITALIGPSGCGKSTFLRTLNRMNDLVEGIKIEGNVIYEGK  NIYSNNFDILELRRKIGMVFQTPNPFLMSIYDNISYGPKIHGTKDKRKLDEIVEQSLKKSALWNEVKDKLNTNALSLSGG  QQQRLCIARTLAIEPNVILMDEPTSALDPISTGKIEELIINLKESYTIIIVTHNMQQAGRISDRTAFFLNGCIEEESSTD  ELFFNPKNTKTEEYISGKFG | Non-Virulent | - |  |  |
|  | 95 | >WP_002657664.1 hypothetical protein [Borreliella burgdorferi]  MRDLFFNNVKLISLFVFNVVICLLYLGFYDASFFYFVLALLLVNFFVIFFYLRYFYSISFKGSKIYYKGIFLSYNFDFSD  IISFEKRLSDNVLILQLKNKKKIKVCFWTGNGSSELFKELKIKRKDLFVPKLENFPIRYYLSCTYLFMFLARIMIALFVY  YISLSNIFIFLFICFIDIKVLLEDFSVISNIVLFYEFRKDSICERKIFKSEIYFYEFFEKIFIAREFEFNNKNYLCFLYK  ENHQTKKVYILNKKISYSMQKVFEYINQNYCTELT | Virulent | **0.70** | **Allergen** |  |
|  | 96 | >WP_002657683.1 DedA family protein [Borreliella burgdorferi]  MTKMYINTIIEYIDSNIAYSPIVFFSLLILAGLNVPISEDAIVLMGGILSSRKNEYTILIFLGIFWGAYLGDIISFYIGK  LMGNKLFKNKKDNNLLDKINYYYGQYGVLTLFIGRFIPFGVRNAIFMSAGMGNMKSNLFIVSDFFATLLSIVVYFTLSFK  LGQSFEIIFSKIKIIIFAIFIAVIATTIIIYVIKKNKKVDKNLK | Virulent | non |  |  |
|  | 97 | >WP_002657688.1 RND family transporter [Borreliella burgdorferi]  MDFEGLSIRYKGIIFTIFILITIFLGFFLKNLKFDANILKLIPKTKETESLIDIDKSNSLLSTIVIFQDKKNIFNKKNFE  TINSVISEITKILKVSPNAVTSIFSYFPQFKKEIYTDKDIEEIKSKIKSTPFVKNTFLGNSENLIYFIIIPSESDQINFS  RNLKTELDEMEKTIKKYETDDLKLYLTGDLIVREKILNYMVEDFKILGPLATFVVIISLYLIIKNLIGALIPIFIALLSL  IWTFGIKGFVQSPITVPETSMIVLLISIGCANAVHIINEIFKLIKKEQLSKESIKATIKKLKTPILLTSFTTAFGFLSLT  TSSINAYKTMGIFMSIGVIISMIISLTVLPGIITLIPFAKKKSFEKEKENKLNKISFLERLAKLNTQITKSILKRKYTSS  IMVLIILGISFVGLLKIEINFDEKDYFKESTSVKKTLNLMQKEMGGISIFKIEIEGRPGEFKNAKAMQILDLITDKLDAF  SAKTQSSSINGILKFTNFKIKKESPLEYKLPENKIILNKLINLIDKSDWTKDNKRMYINDDWSLISIIVRIEDNSTEGIK  KFEKYAINTINEYMKNNKYHFSGVYDKVLIAKTMVKEQVINIITTLGSITLLLMFFFKSIKTGIIIAIPVAWSVFLNFAV  MRLFGITLNPATATIASVSMGVGVDYSIHFFNTFILQYQKNQIYKTALLESIPNVFNGIFANSISVGIGFLTLTFSSYKI  ISTLGAIIAFTMLTTSLASLTLLPLLIYLFKPRVKLASNNNFKKLKQ | Virulent | non |  |  |
|  | 98 | >WP_002657697.1 undecaprenyl-diphosphate phosphatase [Borreliella burgdorferi]  MTNILSAIILGIIQGITEFLPISSSGHLLLFRHFINLKLSIIFDIYLHLATVLVIIIYYRKRILELFLTFIRFSLRKTVK  SDLTNLKLILLILIITIVTGVVGTFISKYESMFTLSFVLINFIITGILILMLEFNFLKVDFKGNILLAGIFMGLMQGLGA  LPGISRSGITIFSASVIGFNRKSAFEISFLSLIPIVFGAILLKHKEFYDIFMVLNFFEINLGALVAFVVGIFSINFFFKM  LNNKKLYYFSIYLFALSIIVCYFVRI | Virulent | **0.58** | **Non-allergen** | **30.2** |
|  | 99 | >WP_002657716.1 flagellar biosynthesis protein FlhB [Borreliella burgdorferi]  MIKDEFLIKSWYIPLDFFSADDEGRTELPTDQKKQKAREEGRVLKSTEINTAVSLLLLFALFFFMLSYFALDLIAVFKEQ  AIKLPEVMRMSVYTMGFAYIRSIMGYVVLFFFASLAVNFFVNIIQVGFFITFKSLEPRWDKISFNFSRWAKNSFFSAGAF  FNLFKSLLKVVIICLIYYFIIENNIGKISKLSEYTLQSGISIVLVIAYKICFFSVMFLAIVGVFDYLFQRSQYIESLKMT  KEEVKQERKEMEGDPLLRSRIKERMRVILSTNLRVAIPQADVVITNPEHFAVAIKWDSETMLAPKVLAKGQDEIALTIKK  IARENNVPLMENKLLARALYANVKVNEEIPREYWEIVSKILVRVYSITKKFN | Virulent | 0.60 | Allergen | - |
|  | 100 | >WP_002657717.1 flagellar biosynthetic protein FliR [Borreliella burgdorferi]  MNLNFLVLKSFTILPVLVRIFMFLKFSPFFSTIKIGYFNFFFSLILSVIVVEKIKIIYPLDNMLSFALILLGEAILGLIQ  AFFVNIIFNVFHLVGFFFSNQIGLAYANIFDVFSEEDSMIISQIFAYLFLLLFLSSDFLLRFFVIGIHDSVLNIRVEHLV  NMRNSGFVKLLLMSFGFLFEKALLISFPILSLLLLFYLVLGILSKSSPQINLLIISFSTSLFLGLLILYIGFPSLAISSK  RVIELSLDSLASFLKLFSRVLK | Virulent | **0.69** | **Allergen** | **-** |
|  |  |  |  |  |  |  |
| Extracellular | 1 | >WP_002656068.1 flagellar hook-associated protein FlgK [Borreliella burgdorferi]  MDSTFSGIEIGKRSLFAHKDAMNTVGHNLSNATKPGYSRQRVTMKTEIPLYAPQLNRAKKQGQLGQGIVVQSIDRVKDEL  LNTRIIEESHRLGYWTSQDKFISILEDVYNEPEDQSIRKRLNDFWESWHDLANQPQGLAERKIILERGKSFCEGIRNRFH  SLERIYIMANDEIKITTDEANNYIRNIANLNKQISKSQAMKDNPNDLMDARDLMVEKLGNIISVSIENKQDPNEFLIHAE  GRHLVQGSIANEFKLEATNGPTRTRWNILWANNDKAYLKTGKLGSLLNIRDEEIKNEINELNNIAANIIEIVNEIHEAGR  GMDKKNGRSFFSQELKLTDDRGRYDTNGNGQFDSVHIFKINSTNEIFPEEKLGFYGTLKFEATNSNEIVEIPYNAPDTVQ  DVINRINNSNAQVTARINSEGKLEIKAVKEQEDENITFKIKHIEDSGSFLTKYTGILNASGPEGAYDYKNIDTTDKLAPK  STYSISPLKNPAAWIKVADIIDSDPSKIASGIKNPTNEISIGDNQAALRISSFGNSQIMIGKNLTLNDYFANTASNIAIK  GQISEITKESQSQILKDLTDLRMSISGVNKDEELANMIEFQQAFIAASKFITVSVELIDTVINKMGV | Virulent | Non- antigen |  |  |
|  | 2 | >WP_002656519.1 extracellular solute-binding protein [Borreliella burgdorferi]  MKKVIILIFMLSTSLLYNCKNQDNEKIVSIGGSTTVSPILDEMILRYNKINNNTKVTYDAQGSSVGINGLFNKIYKIAIS  SRDLTKEEIEQGAKETVFAYDALIFITSPEIKITNITEENLAKILNGEIQNWKQVGGPDAKINFINRDSSSGSYSSIKDL  LLNKIFKTHEEAQFRQDGIVVKSNGEVIEKTSLTPHSIGYIGLGYAKNSIEKGLNILSVNSTYPTKETINSNKYTIKRNL  IIVTNNKYEDKSVIQFIDFMTSSTGQDIVEEQGFLGIKT | Virulent | non |  |  |
|  | 3 | >WP_002657035.1 DNA/RNA non-specific endonuclease [Borreliella burgdorferi]  MKKRSKFFLYCYILCLTGFLFFSLNPKALKQIKHKIYDYLEIIENKYTDITKSIPIKEAQLIPKGYLTTQIISKKHYTLG  YAESARQSEWAAYPLKREMVELALTLLKSKKIKRSTKFFEDTNIKGAFPKLEDYFKSGYDRGHIVSSADMSFSENAMKDT  YFLSNMSPQKSEFNSGIWLKLEKLVREWAISKGYIYIISAGILTENKGFIGKNKILIPKNFYKIVLAINNNNYYDIISFI  IPNEKAKDLDLENYVVSVDLIEKKTKIDFFEKLDSKIKKNIKKIKNTHSWKFK | Virulent | non |  |  |
|  | 4 | >WP_002657111.1 flagellar basal-body rod protein FlgG [Borreliella burgdorferi]  MMRALWTAASGMTAQQYNVDTIANNLSNVNTTGFKKIRAEFEDLIYQTHNRAGTPATENTLRPLGNQVGHGTKIAATQRI  FEQGKMQSTNLLTDVAIEGDGFYKILLPDGTYAYTRDGSFKIDSNRELVTSQGYKVLPNILFPEEYIQNSITISEEGIVS  VKIDTSNEPIELGQIEISRFINPAGLSAIGSNLFKETAGSGQEIAGIPGSEGMGRLRQGILEMSNVSIAEEMVTMIVAQR  AYEINSKAIQTSDNMLGIANNLKRQ | Virulent | non |  |  |
|  | 5 | >WP_002657535.1 tRNA (adenosine(37)-N6)-threonylcarbamoyltransferase complex transferase subunit TsaD [Borreliella burgdorferi]  MKVLGIETSCDDCCVAVVENGIHILSNIKLNQPEHKKYYGIVPEIASRLHTEAIMSVCIKALKKANTKISEIDLIAVTSR  PGLIGSLIVGLNFAKGLAISLKKPIICIDHILGHLYAPLMHSKIEYPFISLLLSGGHTLIAKQKNFDDVEILGRTLDDAC  GEAFDKVAKHYDMGFPGGPNIEQISKNGDENTFQFPVTTFKKKENWYDFSYSGLKTACIHQLEKFKSKDNPTTKNNIAAS  FQKAAFENLITPLKRAIKDTQINKLVIAGGVASNLYLREKIDKLKIQTYYPPLDLCTDNGAMIAGLGFNMYLKYGESPIE  IDANSRIENYKNQYRGKNNEKNFSNA | Non-Virulent | - |  |  |
|  | 6 | >WP_002657544.1 flagellar basal-body rod protein FlgF [Borreliella burgdorferi]  MVRGIYTAASGMMAERRKLDTVSNNLANIDLIGYKKDLSIQKAFPEMLIRRLNDDGLYKFPKGHLETAPVVGKIGTGVEE  NEIYTVFEQGPLKTTGNPLDLALTDQGFFVIQTSDGERYTRNGSFTIGKEGILVTKSGFPVLGEKGYIYLKKNNFKITHQ  GQVFHNSNFESDPKRLVSEYENSWENYELLDTIRIVNFENPRFLKKQGNSLWIDTKTSGKAQEIDISLRPKIETETLEAS  NVNAVKEMVLMIEINRAYEANQKTLQTEDSLLGKLINEIGKY | Virulent | non |  |  |
|  | 7 | >WP_002657723.1 flagellar hook protein FlgE [Borreliella burgdorferi]  MMRSLYSGVSGLQNHQTRMDVVGNNIANVNTIGFKKGRVNFQDMISQSISGASRPTDARGGTNPKQVGLGMNVASIDTIH  TQGAFQSTQKASDLGVSGNGFFILKEGKNLFYTRAGAFDVDSDRHLVNPANGMRIQGWMARDLEGEKVINTASDIEDLII  PIGDKEGAKSTKNVTFACNLDKRLPLIQEGANPADIARGTWVVNKSLYDSFGNVSVLELRVVKDLNTPNLWNATVLINGE  QNSNFTLGFDNEGALASLNGQPGQKGDILQIPITFNVLGANVGEVGEQQTVNLKLGTVGSYTDSITQFADSSSTKAIIQD  GYGMGYMENYEIDQNGVIVGIYSNGIRRDLGKIALASFMNPGGLAKSGDTNFVETSNSGQVRIGETGLAGLGDIRSGVLE  MANVDLAEQFTDMIVTQRGFQANAKTITTSDQLLQELVRLKN | Virulent | 0.52 | allergen |  |
|  | 8 | >WP_038955425.1 variable large family protein [Borreliella burgdorferi]  MMKAAEKAAAAGDGNSKIGDVANAGGGTGGNDASVKGIALGIKGIVDAAGNASGEKGGGGALKGVKEATGNSNADAGKLF  DTNGGGRRAGAADVGKASDAVSAVSGKQIIKAIVDAAEKGDHAGAAAGTATNPIAAAIVLRGLAKGGKFAATDADAGAKD  GKKGDAVTSVVQEVNKWLEEMMKAAEKAAAAGDTGNSKIGDVANAAGGGAGGNDASVKGIAEGIKGIVDAAGKASGEDGG  SALKGVEEAADNNNGDAGKLFATSAGGGNRANAADVGKASDAVSAVSGKQIIKAIVDAAEKGGHAGAAAGAAKNPIAAAI  VLRGLAKNGKFAADSADGAKSAKDGKKGDAVTSVVQEVNKWLEEMMKAAEKAAAVGDTGKSKIGDVAHGAGGGAGGNDAS  VKGIAEGIKGIVDAAGKASGENGGGGALKSVEEATDKNNADAGKLFATQGGRQAGAADVGKASDAVSAVSGKQIIKAIVD  AAGKASGEKDGDALKGVKEAADKNNGDAGKLFATSAGGRAGAADVGKASDAVSAVSGKQIIKAIVDAAGKASGENDGGAL  KGVEEAAAGAATNPIAAAIGQARQNGAAFNNNDMKKDDKIAAAIVLRGLAKGGKFAADSADNAKSAKDGKKGDAVTSVVQ  EVNKWLEEMMKAAEKAAAAGDTGNSKIGDVANGAGTKANDASVKGIAEGIKGIVDAAGNASGEDGGGALKGVKEAAGNSN  ADAGKLFATQGGRQAGAADVGKASDAVSAVSGKQIIKAIVDAAGNGGHTGAAAGTAKNPIAAAIGQAGQAGAAFVDNNMK  KDDKIAAAIVLRGLAKDGKFAAANADGAKSAKDGKKGDAVTSVVQEVNKWLEEMMKAAEKAAAVGDAGKSKIGDVANAAA  GAGGNDASVKGIALGIKGIVDAAGNASGENGGGGALKGVKEATGNNNGDAGKLFATSAGGRQAGAADVGKASDAVSAVSG  KQIIKAIVDAAGKGGHAGAAAGNATNPIAAAIGQANQAGAAFVDNNMKKDDKIAAAIVLRGLAKDGKFAATDENGAKSAK  SVIESARKLTNG | Virulent | 0.99 | Alelrgen |  |
|  |  |  |  |  |  |  |
| Outer Membrane | 1 | >WP_002557211.1 hypothetical protein [Borreliella burgdorferi]  MNIRKLLFCIFFMNISFLLFAGDYKGLDFKIKFFNQSIYRVNSNVFIEVSLSNASESVLTLEIGDINSFGFDFDVTDTTN  IKVKRPIEYVKKRSKNVAIPVRNMSLRPNEKFSVVINLNQFVKFSKDGVYFVKGIFFPDISDPSKKKESNIITLFLNDGF  DENPGSIDLVNLSENNDIQDILKKKKLSPDEIVKYLLKALQLGKKEKFFLYLDIEGLLLNDKGKAYLYKQKLSPIPNKNV  VEEYKEYLWNSNNSDISKAPNKFSIIETTYSDTSGKVIADLYFDDGQFYISKRYTFFFKKYDYYWIIYDYIVQNTGIKEK | Virulent | non | - |  |
|  | 2 | >WP_002655955.1 hypothetical protein [Borreliella burgdorferi]  MRDLILIWIFIIFINNIQATQIESSLKSERIKGSDEKSTNFTFKSDFAQGVVSSFYKKIVLKGNSEVISSDFNLRADEIE  IYGENGSYLEARGNVFYKDYKNKMNVKAQFLFFNRKLDNFYLQKGVELEDLENNMLVKAERIEGSNKTNVYIMQYSVKIY  KDDTFARAENGTYNKEEKEMILEGVPVIYQKDNYYSASRIIFNTKTNRYKLEGSVEGEFTQVENDASEEKK | Virulent | 0.80 | allergen |  |
|  | 3 | >WP_002656010.1 peptidoglycan DD-metalloendopeptidase family protein [Borreliella burgdorferi]  MKKRIRFKIILGFKGILKFFLAIYNSLFIVLKAIHSFFKQNISFIVIPHVKGNVKNIKISFLTLFFFSTFFLGGFIGFVL  LAVNYVTLSSIVKSTEKNYSLAESEIEDFRNTVVEINSVAKNFSKVLDELKTSLKINSNGVDLNKNKLDGDLSDFIDLQI  LEANSIKELSDLKNIKSKIESSIPPLKSIVKVLHAQDKLLNDIPSLWPLAGGSGIITLHFGPAIEPFTRQWYIHKGIDLG  GVRIGTPIVATADGEVVRASYQSAGYGNFVQIKHKYGLATLYAHMSRLNTSKGSYVKKGQIIGFMGQTGYATGPHVHYEV  RVGSQVINPDMYLNLATGASK | Non-Virulent | non |  |  |
|  | 4 | >WP_002656254.1 BB_0345 family helix-turn-helix protein [Borreliella burgdorferi]  MKENDFIKFGSYLRKVRDSKNLTLEMVAEDIKISIKYLKALEESNIEIFPNEVLAVGFLRTYSEYLDIDSRLISTLFKDY  KSRLNNSYIGIKSEDKISNLGFLSDNKVSEKKIFFFSLESLSIFKVFLGIVGVLLLFVFLYFREVEGYFKKFFNLSQDEK  IISNIHEVSFDKKNFWNVSLKEGDFLSLTYSDDIAKYRASFIGDDLVIVDESKKSKNILNLGEFKEINLDDNIRVKIIYE  NYYYDKLKIAHVSLESFALNVKYVSETNIDNRFNILNWQFDVKGTEKLPSSNYLTLYSSQKLSNVDLKIDFLNDTFFRYA  DENNLYGKSLFASKGIPINLAFEKSLILFFSRLSDVNIILNDRDITPFLKEQGKEIFAVQFFWVKTPSGFDLKVSEVY  >WP_002656257.1 MULTISPECIES: flagellar basal body-associated protein FliL [Borreliella]  MPNKDDDNLDMGDSNVSRKGGLLPDIIIKILQILAIGIFTVAIMIIVSYFVSKMVVSQSGAPSDFPVFSNEYLGKPPMLI  WYESIDEIRGNTLDVPPKTFVVKLALGYAENNVNILNELGRQKVRLKDIIREYFSQRTGQEIKNESQIKAEIKARINSIL  RNGEIKEIALTQIDIFDM | Virulent | non |  |  |
|  | 5 | >WP_002656531.1 hypothetical protein [Borreliella burgdorferi]  MTNVDAHEFWALPVCCNVKDSFIEDFSFVDEHVNSIFSISYKDNITLKFRDFVDSNEFNLKLFDNKISFEGQIPLVLYSD  NLDYLLKAEKSVLFNLRVGDANSSYSSGFTKKESGTCNVLNACYEFSDYNSFSDDFILVKIFFDSENLIIDANVDNISFL  KFFISENFGVSKDKIKINSLDNYWSSSIFPISFNAILQGIVISKKINKRVNIVYYKRHFGILDNLNLTFSVSNCLVADNK  LSKIILEIIINRPLNFLYKFYFKFINNIFKNLFFDGFLEIFFVENKSDFIFFYDNLLAFETSVYNFIYSNFYNLALSMSC  EPIGYLLTQIKGDYAGFLKIFKKLDLKNSLIKKSAVLSVNKDYDIFDTSRRGVGFAYLNSSAYFLSEEQYVVAFLYKDGL  NVFLPYSIIDNNLSNYLKNSLAKTLGLPYSSVNFLIGESVLDFGNFYNLLFKDPYLIEKAILSIKDNFSSLIDSDFINEY  PVVFKEKIAISDVNDCVLGCSVELKFEFYSLSAVFSNVSFFVEQGKFTKLKLNNKRIRTIFGLAVDYVFCRANVSCDIKD  CLSLEFIEDGEFIFSFRSVFIVSVSAIRTALIQVFDFNASKTPLDFEEILNSWSIKIDIN | Virulent | non |  |  |
|  | 6 | >WP_002656578.1 hypothetical protein [Borreliella burgdorferi]  MSFNVEEGTIKFKKLKFFLILSLFLLFIILIDFFIRSTMNVSNFYDFKNFENKSDCKNINLSKNVFVSNKVLSLNFGESC  YSLLSDSLISYSDYYYVLFNSGEDYSVFSVKNNKFLFTLKLKDFVFAINNLIFTLNNLYKTLEVYDSSGNNILMLNFLSS  ILSVDYNNEVLVLGLSNGEIYIYKQGKIIYMENFLERKFPTCFVKLSSDNKYLVSLKGSSEYFLEIIDLENNYKKILELN  NLTINSFETFIKIDDYHNLFIEGKNSLAVINIKSGRIFKVENKNSILRASYDYFQNIYRVYFYSESEKIINIKTYSANSF  KLFDNIFIKDEISSFVEFGKGLLYFNSNNDLKYLGLAQ | Virulent | non |  |  |
|  | 7 | >WP_002657109.1 outer membrane protein assembly factor BamA [Borreliella burgdorferi]  MGSIRGLFFVSFLIFFVVFSFGQVENYKGKIIKGINFEGLKNKKERDFINILKPYIGVSYSNEIFDKLQIDLYSLDYFSG  LIKPIFKIDGEDLFITFIVKEKSLVNSVVFSDSSRVFWNSELVEKVNIKTNEPLNLASVNKGIGKLEEMYKDMGYLEVSA  NFEIKEEGNLVDIIFNIVAGPKYVVKGIDFEGNLSFKSSTLRKSLASRVVSLFSDGKYLKSNVDKDKRQLESFYKNNGYI  DVKIINSTVDIKDSLKDSKRLEKEVFLKYFLSEGNVFRFGKLEISGNSVFSLEELKSFITFSEGDIFNDSKFEQDFAKIK  ESYFKEGYIFTEIIPSQKIRGEFVDLLIKILEKDKAHIESITVSKNKNTASHVILREIPLQEGDVFSLDKFKMGMANLQQ  LGYFSNVIPDIVPGNTEGLMKINLNIEERATSNFGFGMNFGGNSNSSFPFSVFGQWELSNFLGEGYYFAARLNLSFLEQS  LSLTFRDNWFFQKRWTVGGFIDFSHSVNTAYQDINGPIFSGKREVPDPFTSWEEYRDAKSFSDFNAMNYSLLKLSFGAFT  GYTFSNYLGKQTLLGTLQTALKYVFYDNEVNRPSNYYLRDNYKTFRFENSLSLSAAWDTRNSTSLSNNGFLLKQQFDFFG  GFLFGQSHFIKSSTTFERYFSLLGYEDVFTPYFDIILTLRSVYSNILPPLGNGFEIEIQPHHHIILSENFMQARGWGILK  NIYSSFVNTVQISIPLLKNILVWDAFFIDFASYSLEGQENSLFRPFSSFAFSWGTGIRSLLPQLPLSFVIAYPFYFDNDK  VNSYYKYYSGFKFFLGIEMRY | Virulent | Non |  |  |
|  | 8 | >WP_002657286.1 LPS-assembly protein LptD [Borreliella burgdorferi]  MREFLYRNVFKKSFIVFLIFLTFSNAIFAQTIDDENSKKRDKLTLSQKSYLRELELSTDEDLKKWALKEGLKETDVSKIR  ELLLKKFGIDPELFIKGKGLAGSGRYKIIIETADNLENFTYGLTKDESIIFEGRVNILVEDIKENKKHNIKGDRIVLNKN  SKKLYAIGNVEYILDMDTNEKLYFYGNEFLVDFDSQNFLLKNGILQKKMQKNQIDHILSFGGKVLKKIDNDVTILEQAFA  TTSKIPEPYYSIKASKIWALPSGDFGFLNAIFYMGRVPVFYIPFFFRPGDSLFFNPSLGLNPRKGFSVFNTVYLFGNKSS  SEDSSFLDFDFNSVYNSGKKPYIRNGYLTYFFAENLAPSVNKDYVKLIFDIYANLGFYSGIDFNLGNTLGHFKTLEGNFG  LGFTRNVYSYDGGYYPFDNRTLKQSLFSFSNLNKGDVFGFEVPFRYLFKFKTEFLLSDALFSVVLEHYSDPYVNIDFRDR  IESATFFSLLNLDKDSVKEQTSISTFDWNLSSFYKRTFNDGSILDYKLNNLGLNFKLSDYENLYVKSPLEKPKDVNDPTR  KWFYLERIYAPYIDLNFQKDLYNNQWTFSTDTKEMIMRPEIKNLEDKDNDKKSVKEKNTKKTTELTKDLYIPPEPITLKN  IDQSDSFFIRFGINPYLRNNVFFDNYGITSPKDFNYQIKNYLFDIKNKTDIKIHADFYNRLITFENLLYLNTIEYSPLNK  DFKVEDKDKKSEHSIINQINLNLLPFIRYPLFSRSTLKFENKATLYSFNKKYDSDVKSLVNKNSSIFLSDPETFYQSLTA  SLIYDYDYFTTELSGELKNSFEDIKASSELKLSLDFPYLLQEAGIGIKYYKKFKEDAMKNSGISAVQSPLEPQKPSSPYK  NLEMSPALYYKIEPRYLDYFKFSFLVAYDPLINRVSELSFKLNVFDFQFLFAMKDDFEYNYDPLKGDFSKIGTTTKLVPY  SLDSSYKKELYVLTFFDNKLSFTLGVDVGWKINLQKFTDNELRSALTLKLKYTEFLEIYFSTLSINTKTFKYFKGYMDQI  GLEPVNVFVDLSKSFNFFNSQDRKDSLFKIKKFSSGFKFNFYDWKFVGEYNLEPDLLRGSDGIYSPIWRNNFTIYISWNF  FAPIKASFENNKDTNYELIINRKTKK | Virulent | 0.611 | allergen |  |
|  | 9 | >WP_002657484.1 septal ring lytic transglycosylase RlpA family protein [Borreliella burgdorferi]  MRNLIDAILRDNKNFVFLFVFFFIASHLNSATVGLASWYGEAFHGKTTANGEKFDMMALTAAHKELPFNTTVKVTNLLNN  RSVVVRINDRGPFRKDRIIDLSKHAAEKLDFLGIGVAPVKIEVIESSNEKRPSVSKSSDFKKTFNIPEVKEEKKTKQSDE  NISVENKPKDLLADYSVSADKETDFYIQVGSYRKKDYADRAYRILKKAGLFVVVNSHGPFYTVFIPTNADDVQKNIELIK  SAGYKDTLIRKTKVPGESLIMD | Virulent | non |  |  |
|  | 10 | >WP_002657669.1 hypothetical protein [Borreliella burgdorferi]  MLIFGFIGLFFLNIFSLHAQGIVTNKDAQEEFKWALNSYNNGIYDDALLSFKKILSFDPNNLDYHFWTGNVYYRLGYVEE  ALMEWRNLKDQGYKVPYLRHLISTIEQRRGIFSNYELNFKKLVKVASLDNSIYKRPHGYQITSLRADKYGGYYAANFVGN  EILYFDVNNNVNALVKDGFSYLKSPYDVIEANNLLYVTLYSSDEIGVYDKVLGVKRKSIGKKGTKDGELLAPQYMTIDKR  NYIYVSEWGNKRVSKFGLEGDFILHFGSRTSGYKGLLGPTGVTYLNENIYVADSLRNTIEVFDTSGNHLYSVFTSIEGIE  GLSSDFVGNNVIVSSKDGVYKYSIAKKTITKILKADKMNSKISSSILDANNQMIVSDFNNAKVSVYKSDASLYDSLNVDV  RRIIRLGGPKIYVELNVSSKSGLPVVGLKSENFSISNENYYIVNPKVAYNVNASKDINIAVVFDKSSYMKKYDTDQIVGL  NALMELSKNKNFSFINATSVPIIDNIESLTNSIRNTSSLGPYSTDAVKTDVSLKLAGSGLMSKSSRRAVVYFSGGILNRK  AFEKYSLDTIVSYYKNNDIRFYLILFGNDPINSKLQYLVNETGGAVIPFSSYEGVSKVYDLILEQKTGTYLLEYYYPGPQ  EPNKYFNLSVEANINQQTGRGEFAYFIN | Virulent | non |  |  |
|  | 11 | >WP_002657705.1 M23 family metallopeptidase [Borreliella burgdorferi]  MIIPKKKQRVEKRKKNFLFNSKKSVNFELKDFANISNIGKRRKKVFKIKNFFKKKINFFKKVSLFFYKLKIQNINHYEYK  YYYKSLRDKVFDIFSVRFDYKLVFKLNAIIFIFILTFYINIFSYYGSYVFLNRLSLPKDYFIDTFLYYSDQDIAQISSYL  PESNVSANVPGFKKNFVLKVFDHKIKPGETLSHVAARYQITSETLISFNEIKDVRNIKPNSVIKVPNMKGIVYIVKKNDS  ISSIASAYNVPKVDILDSNNLDNEVLFLGQKLFIPGGRLPKDFLKEVLGETFIYPVQGVITSGYGYRPDPFTGVISFHNG  IDIANLANTPIKASREGVVVTAGFNAGGYGKYIVISHSNGFQTLYAHLNSFAVKVGKKVSRGAVIGYMGSTGYSTGNHLH  FTIFKNGKTENPMKYLR | Virulent | non |  |  |
|  | 12 | >WP_002657819.1 hypothetical protein [Borreliella burgdorferi]  MYKNGFFKNYLSLFLIFLVIACTSKDSSNEYVEEQEAENSSKPDDSKIDEHTIGHVFHAMGVVHSKKDRKSLGKNIKVFY  FSEEDGHFQTIPSKENAKLIVYFYDNVYAGEAPISISGKEAFIFVGITPDFKKIINSNLHGAKSDLIGTFKDLNIKNSKL  EITVDENNSDAKTFLESVNYIIDGVEKISPMLTN | Virulent | 0.60 | Allergen |  |
|  | 13 | >WP_002658108.1 DNA-binding protein [Borreliella burgdorferi]  MAIFFKNKYFYLSLIFIIFLFLFVFSGFLFYSKPIIYDISPIPTSHKDIIIIKGNNLGYSTGEININNNYLVKSSIISWD  NTEIVFKITDEVNSGLIFVKGERGTSNELFLVISRQVPVKLNRKNIPFIFSEDKIILNANSSTLLQGMNLFSPFSTITIF  LETKDKLYTILPQNILDVSENRVEFVSPKTLNSSGKLYVLLGNIQSNKVSFSVKNDFFKWTLSDSKEFAIIEEIYFSQDV  SSNFDSNPQDINFNIFYLRPIENERQKITERNSEHLDFNIDNLFFENLKTNKFIFKTRVKTYKLNLEFLDAKYLESIEVN  RDINNQEYKKYVQDKKKDYLSYSYVDLMSLDSLILSKTSGSNSVYKLAKAIIDVLTSNFKIVENNLSLKDSIEEKKISSG  NLIVLTNLLFLKYDIPLRNIVGLYYDSNSLKLKEHFWFEFFLAGVGFVYFDIINAVLFKDSSKYFLNISDNYIQYGCKED  YDKNEFFDGYLDSGFLKYKSLTNGSYSLMHRFVLEDNF | Virulent | 0.522 | Allergen |  |
|  | 14 | >WP_002658111.1 integrin-binding adhesin P66 [Borreliella burgdorferi]  MKSHILYKLIIFLTTSAAIFAADALKEKDIFKTNPWMPTFGFENTSEFRLDMDELVPGFENKSKITIKLKPFEANPELGK  DDPFSAYIKVEDLALKAEGKKGDQFKIDVGDITAQINMYDFFIKISTMTDFDFNKESLFSFAPMTGFKSTYYGFPSNDRA  VRGTILARGTSKNIGTIQLGYKLPKLDLTFAIGGTGTGNRNQENDKDTPYNKTYQGILYGIQATWKPIKNLLDQNEDTKS  VIAETPFELNFGLSGAYGNETFNNSSITYSLKDKSVVGNDLLSPTLSNSAILASFGAKYKLGLTKINDKNTYLILQMGTD  FGIDPFASDFSVFGHISKAANFKKETPSDPNKKAEIFDPNGNALNFSKNTELGIAFSTGASIGFAWNKDTGEKESWAIKG  SDSYSTRLFGEQDKKSGVALGISYGQNLYRSKDTEKRLKTISENAFQSLNVEISSYEDNKKGIINGLGWITSIGLYDILR  QKSVENYPTTISSTTENNQTEQSSTSTKTTTPNLTFEDAMKLGLALYLDYAIPIASISTEAYVVPYIGAYILGPSNKLSS  DATKIYLKTGLSLEKLIRFTTISLGWDSNNIIELANKNTNNAAIGSAFLQFKIAYSGS | Virulent | 0.65 | allergen |  |
|  | 15 | >WP_002658164.1 hypothetical protein [Borreliella burgdorferi]  MLFIIFFISAPLFSKEVYYRFADYSVNYSIVGKYEITKEESEEEYGYKFTYDRNNNLILVDYIGKLSILMPSFFNASQIK  IERSKRSERRIFLNRGLAFKNNNGVYIEHIEYMSDGRIKNIFNYNRSNEMVRDKYDVSYYHFLYDNEREFSVYRFNESGI  QIKDLNNVYFTKISYDHTKFVKTVLYYDENGYIVRSKNGTFGFRLTYDANHNLIKEEYLDKSAYAVSNPFSVATKIYHYD  VDGKVIEILNYDINNNLTPDENNVAIYRYEYYFKDKDCYYKEYNYDNNNNLTISQNGYAMKKTIFYIQNNEKRIINYSNK  INKNYNRDIPTVYEEKYEIMDNFKGIAIYSYKYDDNFYLIENIFFDKNFNLIADVKGVMIYRYSYDKEGFLIAQEHFGGD  FVNPIDDFEGVSKYKFSYDSNGNMISKKNYSKDGVLVADCNFVFEYLYEYDKQNRLISQKNFGSLGQLQDDIHGVSVYKY  EYNKFGKISKQSNHGPDLRLRDDVGGFSIYKWIYDSRGNLIDFQKFDSLGNLI | Virulent | non |  |  |
|  | 16 | >WP_002658246.1 hypothetical protein [Borreliella burgdorferi]  MRFKKIFLIIFIISNLKVYSYNYAIQYKNEGIDKYYFEILNDGFGFSLSDFFDDLRSGSLIFTYVSKYNFIINLEAHMLT  YRGYKDSPKSLISRTDLIEIGFMYYFPILLLINGKNFGEIDLGIGVKNLLFGDWGGHLMQSIIHLILNQHRPIPSIKSYD  SYNYRGFLSFALNYSYMNFLNLENYMDLSYFADYFIKNSIGITLKNENIGFDIKLYSQIQNQIKSLKTYSKTQEAETGIG  INYQFYSKNFFITNNLNIKNFSTKENFLSIGGFGIIITPEEYKKISESNNEFNVISNNFYFGFDIMIPLKIRNSLFYKIN  ENINHYFSISTNYYTNYNETNSFTNQLSSGIMYEFLPQKTFNPYLISGLFFAYNQNNKDIKSISRPIRIKNILQVGIENE  LGFLFKMLKYRNTEYIFKIYSKVNYIPIAYNLDEKKLEKHSINFNYLGIGIVVK | Virulent | 0.57 | Allergen |  |
|  | 17 | >WP_002658342.1 SUMF1/EgtB/PvdO family nonheme iron enzyme [Borreliella burgdorferi]  MKEVDENSNVELFEVKLKPILGIEPKVYVFLTTIILLLSLISTLIIIPKFKNPGAYLKINSNIENTYIYLNEKYIGRTPL  NKYINATEGVLRAKRMGFKTYEQRIKIHNKFFGNYSLQINLELVDPEKIIKQRQKELSIMVKIKNINENTKLIPVFSLIS  SELKEHPKYIKKFLKDSIPYLNSTEMFKDFLNSYKAIYSIDQDNSNQEEIWNSLKTNFDLENRAIFWFLENLDKDLKILT  KNAPWVKTLAKTLDNENIQLISKNEKINIKLPGFKKINSNKIEKIQNYELNSLDSKNISLKSTYNVKEFLIQEQNVTKYE  YQDFLKENPKWALNNKENLIKEQLVDENYLKNFNQIGLNEAITGISYFSAIEYANWYSKKLPTGFKARLPISQEWELYQK  EPNKNPLNINEISKKVGFWNLMQNSSFNEIAIFKNEKNFYSENSNFYSLITEIRTYSQQNNNLLNASTKASFLKNWSSPN  IGFRLIVSKE | Virulent | non |  |  |
|  | 18 | >WP_002658697.1 DUF685 domain-containing protein [Borreliella burgdorferi]  MADDQEKLLIDEEETVQIKDLNKVTTVNNTDLLLLDDGAASSNAITFKNFLDASKDKIFKGEGLDYFKQIIKSTIAEELA  ADKDFVEKIYAKITDKLINNDSTNISNLFSKIKSRLTNSISSATLSKNDDLLIMSSSSIQKTPIPEQLLGVPSDFQSSYD  FTRNTIIYPRDYKNHRVIIDLEDYNDVDLIFYKSDDDDPIYLDFQVDVESFGEGKTLSLRYSDEREKNTIYSRNSSSTRR  ITFSIPLYKGWYVQKRAYSSGNPIPVLLKL | Non-virulent | non |  |  |
|  | 19 | >WP_010254694.1 hypothetical protein [Borreliella burgdorferi]  MKINKTFILLFLFTKFSFVQAQANQILTEISPLSILSKNGKGSVYLKVSKSSDYILTLDKSSNSDFVFKIYDISNKKYIT  DKVKRRDFKIRLDKNSLYAIIYVGTKNENIKFSLTDLDFSILSSDSLKAKTSKIEKEDLFFTLKDLPVLNLTAKLKKYVL  RIYKSNIYIAYQLENSDDIKVAEFIEDVGWFNLDSSVNRNITNIVNFDFSINSKGNLYIAFVTKSGADFASELIVKKFNS  RKWIDISPGHIENFGSLLNISIDLKDRLYLAYLREIRGEYKINLISNMGYGSIWTDVIHAYLSKGDSNVNSSNIGLISEP  FLGIFYNYKSNNEIKSEFIVNNENAWVNANIPSVYMANFIKGFFDSNFNQIIMSFVSENRPIVNICPLKSSRWINISPNV  EMEGLSADIGLYKNNLFLAFEDNNNVRLIYFKNKNWYFLNKLENFKSNVKSPQIGIYGNQGLVISTLSSNSNELFFTLIC  Q | Virulent | non |  |  |
|  | 20 | >WP_010890379.1 outer surface lipoprotein OspB [Borreliella burgdorferi]  MRLLIGFALALALIGCAQKGAESIGSQKENDLNLEDSSKKSHQNAKQDLPAVTEDSVSLFNGNKIFVSKEKNSSGKYDLR  ATIDQVELKGTSDKNNGSGTLEGSKPDKSKVKLTVSADLNTVTLEAFDASNQKISSKVTKKQGSITEETLKANKLDSKKL  TRSNGTTLEYSQITDADNATKAVETLKNSIKLEGSLVGGKTTVEIKEGTVTLKREIEKDGKVKVFLNDTAGSNKKTGKWE  DSTSTLTISADSKKTKDLVFLTDGTITVQQYNTAGTSLEGSASEIKNLSELKNALK | Virulent | 0.92 | allergen |  |
|  | 21 | >WP_012665570.1 outer surface lipoprotein OspA [Borreliella burgdorferi]  MKKYLLGIGLILALIACKQNVSSLDEKNSVSVDLPGEMNVLVSKEKNKDGKYDLIATVDKLELKGTSDKNNGSGVLEGVK  ADKSKVKLTISDDLGQTTLEVFKEDGKTLVSKKVTSKDKSSTEEKFNEKGEVSEKIITRADGTRLEYTEIKSDGSGKAKE  VLKGYVLEGTLTAEKTTLVVKEGTVTLSKNISKSGEVSVELNDTDSSAATKKTAAWNSGTSTLTITVNSKKTKDLVFTKE  NTITVQQYDSNGTKLEGSAVEITKLDEIKNALK | Virulent | 0.82 | allergen |  |
|  | 22 | WP_012686303.1 right-handed parallel beta-helix repeat-containing protein [Borreliella burgdorferi]  MEDQNNSSQEPAELSQEISDLSQETTEKNSEISGLSQKAEKDNETEEISSAKEDTTEDCTSLGLDIVFYTDEGQIFELTP  FVDISSIVLEEKIVDPSLKTAASQFSFSVSGLSEEFLNFLFFRNEDIFVKVNNNNTPVFRGILEKFFSRDLFNSIKSVSF  TVNDYSSLLKIAFENPVQFPINFVPDWLFVYNPLVKELSLVHLIIEKTKLKDLIDDDASEAILDKVLAVIIDSGEDVETI  LQALLYEFGYAYTFSSVGKLQILPIWKSEVINKDVEICSLDAESYVMSKSSSSSYDSTKVIWREGKFQSKEEAMSKKRPL  YSAPINVAGNDGKLYVAVLQKGVVYPDFADKVGSSVFQEYDPSWLDTVYKWDFKRREVWHDHYAINEHLAIISTHNLEAR  FNADSYIKLENKEYFPTKAKLWFRNTSSSQGSRYIYHFDIYGDVFYTIARNVLQTDNADDFYSKKFEYTTRFIFSEESAL  RLFEFLTNLRVKGHTIVNFRSMQNLNLTDFVKLRLENIGIDHFFLILSKKITNFDLDTKLYEYEGITWGDYTHYEYLTTS  TKIGISDYLSGVHKVVIAPFNYDGPETYQFKATGFKDEDTINGAIEFATQAGLNKIKLLKGDFYIYGQVKLNNLEIEGDD  EVFIRSAGFSKHIFTSEKSFKLTGVHICQQPMSELYINGGDLNQEELSSYLEDKSFTRPTDLMLCSSYSVQEEARSSVYV  TDANFVYIKNVTFLCARNKALNFKKTKKILLEDVNFDSTNQSFDTENVSNLTLINVNAQGSKTASVANGLNAIIRESSFS  NNTDALKLSKFSSLKITDTQFSKNTGTALHLADITSARLSNNTFTENGIGLQNRAFDLIIRDTFLRNTLALDLLKESDTD  VRTLDLSTYMENTKDKQEAA | Virulent | 0.50 | Allergen |  |
|  | 23 | >WP_012686354.1 variable large family protein [Borreliella burgdorferi]  MRKISSAILLSAFLVFISCKNNVGEAETKDDKKDSVSMFYQSLVRLGNGFIDVFNAFSGLVADAFFTADPKKSEVKTYFD  SISKKLKSTKEKLESLSNKKENSNADSADGAKSAKDGKKGDAVTSVVQEVNKWLEEMMKAAEKAAAAGDGNSKIGDVANA  GGGTGGNDASVKGIALGIKGIVDAAGNASGEKGGGGALKGVEEATGNSNGDAGKLFDTQGGDGGQQAGAADVGKASDAVS  AVSGKQIIKAIVDAAGKASGEKGGGALKGVEEAADKNNGDAGKLFDTHGGNRANAEAVGKASDAVSAVSGKQIIKAIVDA  AGKGDGDHAGAAAGTATNPIAAAIGQANQAGAAFNNNDMKKDDKIAAAIVLRGLAKGGKFAAADADNAKSAKSVIESARK  LTNG | Virulent | 0.98 | Allergen |  |
|  | 24 | >WP_012686626.1 outer surface protein C [Borreliella burgdorferi]  MKKNTLSVILMTLFLFISCNNSGKDGNASANSADESVKGPNLAEISKKITESNAVVLAVKEVETLLASIDELAKAIGQKI  ELNGGLSADGNQNGSLLAGAYAISALIKQKLDVLKGPEGLNKEIDEAKKCSEAFTKKLQDSNADLGKHDATDENAKRAIL  KTHANEDKGAKELKALLKSVESLAKAAKAASSNSVKELTSPVVAESPKKP | Virulent | 0.65 | Allergen |  |
|  | 25 | >WP_038374813.1 translocation/assembly module TamB domain-containing protein [Borreliella burgdorferi]  MNLLFLRSKTFILLILPFFIFVLIIFSINLFVQAQIYSAKFFAIKYLESKFGFKIKYDKISPYFLSSIKIDGLELSLDGK  DKILIDIVRIDLNLFKLILGDENIILNVYVKGSNFNFDINDFSLSGDLNPSNAYSDNENAVFNKILNYLYRLNINLENIN  INIKLNDNSWLNFQVKNFSLSTVDEDFLFSSVVDFSAVKNLEINLPFERVDDGILDSTFYFEGKFKKGFEDGYVNFSFFE  FKTSYFSLLEQGFQINYSKGNLKIFNLRRENFDFNLSYDKANGFVRLDALFFNVNLLDWIKLNKGFEIYKDYFDISLNGQ  LAFSYDFKDKDLRYAGIIDSSLNVDTIGKEVQGLQLEIKGDDRIVSVKNAFLKLKRGVVNYKGYYSLKDLLPMGRLNFKS  AKILNFNDLNGYLDFNKDKDIFSVKSDNFSLGNLSFQNLILKTYFLKDKIFVDYLIYLENKNSQISLKGDLNDEEFSLSL  GIKEFPLLFLKEVIPSSHLINFFPKTLFSGKYLNLVSDFNLNKFNYHKNKLKKFNFMVFSKLDNFKFVLDASGEKNFYKS  NNFDLQYNDHNLHSNLFVELFESGFNISTNFSYLNKVYPLHFNVNLKDKFIVAESPLGIKLDFNYFDSKVVYTLNVNNFK  VYNKTSDLLININFYGNYLGLNKDFKFKIDEFNIIKVSKTPAYNFNFGFKGLYEYDKLYISDVKLINKLSNLQGYGQFNL  KDNFNGSLNLFSSLNSERYFLGVNSVEYGSYFLLKFQDLDFYNFNSFSLLEGKVNGNFLLNFKKNDFKNYSLSGYLEADK  LTFLGVPVQFSMNLGLLDNKLNIYDIKAKRNKKEFLTGSLRYDLSSSIGVSNIVFNSDLFSYRFNANFRNFQSGVEEQFG  VLKTKTEGEFFFRDIKYKNESLSNLTIEFSNDFEKFNMASIDYDLINVLYHYGSGEYSFILKDYLPLSFNSSGKIIKNKI  LGNVRDIKFDSKIITKDFLDSHSLFNVDNHFILYDLVLNGEFDIDGDLYNPNINGSLNIQKGSISTEYLRASRKFGGDRA  LEIFDMPVAIQDNKIIFSNEFNLDRYSKIFVSTSLNLNFLSDTIIDYYKIDINVTGRTGVPIKFDKIALSFTGYALGDFS  IEGNADEIMFKGILNISNAWVYSLESSVVDLLINPFKRAKRLQTDINLLDFDILTDLEINFDSGVTFHWPDSNISFLQAT  ISRGDKLVIKSDTKTDDFIIKGDLNIASGFVNYNNKKFIFKSGSYISFNESRAKFDPWIKAEATNTIKDRNDKLLVTISI  DSPLSLWKIEFMSYPSRNEQEIKYLLSGSTIGGYEGGLRSAGTNAAEMAIGIVSDIALDFLIQPIEDYMRSVLNLDLLSI  KTDILKNSINSNFFKIGNPTFVDVLDNTSVKVGKYLVEGVFISGGFGFLKEQMSPFSKDLNFVVNLGIEFDSPFFLVNYE  FDYNFMKKGLDGIGNNIGISWKFKY | Virulent | 0.64 | allergen |  |
|  | 26 | >WP_038376999.1 tetratricopeptide repeat protein [Borreliella burgdorferi]  MKKNKTLVFISLFLLSVFLGGFYFYFDPNILYLLKGEKDFSKLIMGIDFYIDKKKFADAKKAMLFSSYYANTEFKWLALA  KRAKLWALNTGDYHLMGEIVNLGVKVLPGNLKLRALEVYSKLKIGLLKDAYRIANEYLLNSEEYQGLYDEVFIKNLSSDN  GILDFNKFIDKIYKEKDARIFEEIGLNLKNNAFLINAMLLYIEKKNIDSAKRILFRIKEDKSFLKELAYISYNLNDLDYT  ISNLKSIKNEEDPTLLFLLADAYFKKGDIKNAKNEYLKLYTRFPDYNVLVYLNLALIANSENDYKRAISYLNKANEVFKD  NKIINYYLANIYFGIKNYFKANEIIANYKEDPLFFKLYFALNYANSKYEAKKSYLWRLFYKTEYNSSIAQFLAWNLLLYS  DLKDLDLFFRIYNFSENKQDWYDFYKFYYYFLKRDFISSKKIIFDNKSQKYMFGIYYNLGVLSFSEKDYEEAERYFNKGI  SLLPSSFYDKDSTTPYERELVSKIYLKQGINYLYLGKMEKGKESILASYSFYETDEGRLYKNMIDTLRERNLNFD | Virulent | non |  |  |
|  |  |  |  |  |  |  |
| Periplasmic | 1 | >WP_002556748.1 flagellin FlaB [Borreliella burgdorferi]  MIINHNTSAINASRNNGINAANLSKTQEKLSSGYRINRASDDAAGMGVSGKINAQIRGLSQASRNTSKAINFIQTTEGNL  NEVEKVLVRMKELAVQSGNGTYSDADRGSIQIEIEQLTDEINRIADQAQYNQMHMLSNKSASQNVRTAEELGMQPAKINT  PASLSGSQASWTLRVHVGANQDEAIAVNIYAANVANLFSGEGAQTAQAAPVQEGVQQEGAQQPAPATAPSQGGVNSPVNV  TTTVDANTSLAKIENAIRMISDQRANLGAFQNRLESIKDSTEYAIENLKASYAQIKDATMTDEVVAATTNSILTQSAMAM  IAQANQVPQYVLSLLR | Virulent | 0.67 | allergen |  |
|  | 2 | >WP_002556893.1 flagellar basal body rod protein FlgB [Borreliella burgdorferi]  MNDFERSVDFSHRYLDVLSLRQSVISDNIANVDTPNFKRSKISFESELEKAFLNEDKNDLNLIKSSDKHLSGFKNLKYSD  VKPHRVLDHFSTMNNNGNNVDIDSEVKALVQNQMMYHLMTNVQAHYFKSINIVLK | Virulent | 0.50 | Allergen |  |
|  | 3 | >WP_002656539.1 flagellar filament outer layer protein FlaA [Borreliella burgdorferi]  MKRKAKSILFFLLSTVLFAQETDGLAEGSKRAEPGELVLDFAELARDPSSTRLDLTNYVDYVYSGASGIVKPEDMVVDLG  INNWSVLLTPSARLQAYVKNSVVAPAVVKSESKRYAGDTILGVRVLFPSYSQSSAMIMPPFKIPFYSGESGNQFLGKGLI  DNIKTMKEIKVSVYSLGYEIDLEVLFEDMNGMEYAYSMGTLKFKGWADLIWSNPNYIPNISSRIIKDDVPNYPLASSKMR  FKAFRVSKSHSSKEQNFIFYVKDLRVLYDKLSVSIDSDIDSESVFKVYETSGTESLRKLKAHETFKRVLKLREKISMPEG  SFQNFVEKIESEKPEESSPKN | Virulent | non |  |  |
|  | 4 | >WP_002657351.1 ABC transporter substrate-binding protein [Borreliella burgdorferi]  MKKIFILIVILTTFACTNKDTITLNVFNWAEYIDETLLDQFEKENNIKINYEIFHNNEEMMAKFNNTKNYYDIIVPSEYL  IQELIDEGKIEKLDYSKLPNVTKNITQNLTNLEHDPGNLYSVPAYWGLMGILYNKTKIDLNDMQGFDILFNKKYKKEITM  LDSPKDNIGVALKKLGYSINEHDTDKIKEAGELLKIQNPLLIGYFSDVPAKSLMLNGEASIQLTWSGEAQNAMLKDKNLD  FYAPENTNLWIDAFVIPIDAPNKNLAYKFINFLYENEPSYKNFKETRYNSPNKNVIKRIEEEAKNNPEMKLYLEEKFLPK  DFSKFEIFKKIPKKIKEEILKIYLNLSS | Non virulent | - |  |  |
|  | 5 | >WP_002657735.1 flagellar basal body rod protein FlgC [Borreliella burgdorferi]  MGLFSSINVASTGLTAQRLRIDVISNNIANVSTSRTPDGGPYRRQRIIFAPRVNNPYWKGPFIPDYLDNGISQGVRVASI  EKDKSPLKLKYDPAHPDSISSGDKKGYVELPNVNLVEEMVDMISASRAYEANSTVINSSKSMFRSALAILQG | Virulent | non |  |  |
|  | 6 | >WP_002657773.1 peptide ABC transporter substrate-binding protein [Borreliella burgdorferi]  MKYIKIALMLIIFSLIACISNAKKEKIVFRVSNLSEPSSLDPQLSTDLYGSNIITNLFLGLAVKDSQTGKYKPGLAKSWN  ISEDGIIYTFNLREDIVWSDGVAITAEEIKKSYLRILNKKTAAMYANLIKSTIKNAQEYFDETVPESELGIKAIDSKTLE  ITLTSPKPYFPDMLTHSAYIPVPMHIVEKYGENWTNPENIVVSGAYKLKERSINDKIVIEKNEKYYNVKNVEIDEVIFYP  IDGSVAYNMYINGELDFLQAAEKNNLEEIKIRDDYYSGLKNAMAYMVFNTTLKPLDNLKVRQALSLAIDRESLSKIVLKG  SSEPTRNLTPKFDNYSYGKQLTLFDPENAKKLLAEAGYPDGKGFPTLKYKISGGTQTVPEFLQEQFKKILNINIEIENEE  WTTFLGSRRTGNYQITSMGWIGDYFDPLTFLESLFTTENHFFGAYQYSNKEYDALIKKSHLEFDPIKRQDILREAEAIIV  EKDFPVAPLYIPKSHYLFRNDKWTGWVPNISESYLYEDIKTKK | Non-Virulent | - |  |  |
|  | 7 | >WP_002658181.1 flagellar filament capping protein FliD [Borreliella burgdorferi]  MASGFFVPGLESKYNTKEIRESMLKSDKAKIDSSFKKLESLEQEKSAWQLINRKISTLNSLAKELTSLNSPFNLMSGNSS  NSEVLTLSTRYGSKNETHKLIIDQIASADVFLSSNFDPKKVTIPEGDYIFLVGKKEINVKSNGNIDLLVKDINNKGKGFL  SAKIVKSDKNGNSRFVLQSLKEGKENKLVIKGEGLSFAKQIGILSELKTNFNPNLSDIVVNQSSSNNKLAFENNGLVLNP  LSEVSIEIPEDIEITSRSKIKFEVKYFDTGLEEPDSKIIFNPGGATFKDAKVESEDSVVDLGSDLKTPLEKKYIQMNMVK  ICSKEGSLELPLINISNNFEEVEVDVGALSNLEEINIENKANNKVIVISNVEIFDPKNRDGHLPINAKSFAENAKIKFDG  VDVERDSNVINDLVPNVTLSLKKASSDMVEAKIEPDYEGIKRVLLDFIGAYNEVLAEINIVSSNEDQPNNQKSNIVEELT  YLSDSQKEEAYKNLGILRSEFLLKNLKSKLESIIFKPYVTSDPNFSIINQMGVFTNSISSSGGLSRYLRLDEKKFDESIR  NNIDNVRELFLYDLNGDRVYDNGIAKMLGDCLSPLVASGGVIYNKIKNYDLKIFNQKNKVEDYKKKYEDRERKVEGELNT  LDFTVKRMKDQENTLKAFDFNQRNK | Virulent | 0.52 | Allergen |  |
|  | 8 | >WP_002658190.1 efflux RND transporter periplasmic adaptor subunit [Borreliella burgdorferi]  MNLIFNINLYLKKYFLVLFLVLVACVGDNKLDDKNIDKEKESSYRFPVIAMKVKKGILSDYLSLNGDVDTKVKADIFPDA  VGKITSLRIKLGAYVQKGQIVATLDPSRPGSVYLKSPVRAPISGYILNITKKIGETVNPQSNIAVVGRIDTKQILTYVSE  KYISNIKVGNDAIIEVGAYSNEKFKAKVSEISPILDSKSRTIEVYLTPIGSNLDKLIIGMFSKIKLITKRFKDVIKISRE  AVVEREGKKFVFKVDLESKSVQMLPITVLFEIDNIVALSGEVEENDLIVVEGMSALSNGSLINLVDTKEGLSAESNI | Virulent | 0.54 | Allergen |  |
|  | 9 | >WP_002658254.1 DegQ family serine endoprotease HtrA [Borreliella burgdorferi]  MEKKFFSGFLLSFLALSIGFFIGMHYLASNRSNIVFAEEKDNTVRALQDSFREVSKKILPSSVEVHATGVIKQSFPIPFF  FFDMPEFDSERKSNWAGSGVIIGRDSQKKSLFYVVTNSHVVDKATELEVVSYDKKKHKAKLIGKDEKKDIALISFESDDA  TIKVADLGDSDKLEIGDWVMAVGSPFQFSFTVTAGIVSGLQRSANPNLQSRNLFIQTDAAINRGNSGGPLVNIKGEVIGI  NAWIASNSGGNIGLGFAIPVNNIKSTVDFFLKGKKIESAWLGISFYPLKTRDSEVLKSLGVESNDVSAAIIASLYPGSPA  VKSGLRAGDIIMKVNGVSMSVFQDVTSYISDFYAGEKVNVEILRGNVKKNIEIVLAVRPKDKELSSSKMLPGFVVYPLVE  DIKAQLNLRNWIKGVVVDYIDKNLASNIRMKSGDVILSVNSKSVSNLREFYDALEVGKNTYKILRGNDSFKITF | Virulent | 0.65 | Allergen |  |
|  | 10 | >WP_012593130.1 peptide ABC transporter substrate-binding protein [Borreliella burgdorferi]  MKILIKKLKVVLFLNLILLISCVNESNRNKLVFKLNIGSEPATLDAQLINDTVGSGIVSQMFLGILDGDPRTGGYRPGLA  KSWDISDDGVVYTFHLRDNLVWSDGVAITAEGIRKSYLRILDKETGSSFVNMIKSVIKNAEEYFDGKANESELGIKALDE  KTLEITLKSPKPYFLDMLVQQTFVPVPMHVIEKYGQRWTDPENMVVSGPFKLKSRVLNEKVVLEKNNKYYNSKDVVLDSI  IFFVTDNSITAYNMYLNDELDAIFKNVPPDLLKDLKLRDDYYSMGINSTSFYSLNMKVKPLDNVKVRKALSFAIDRKTLT  ESVLNDSSIPTRRATPDYIDYSYKSNLSLFDAEMAKKLLADAGYPNGNNFPLLKVKYNTSDSQRKIAEFIQNQWKKNLNI  NVQLENEEWSTYINSRVNGNYEIIRAGWSGDYADPMTFLSIFQTENTSFSSYGYSNSEYDELLIKSDNERDIFKRQEILK  KAEAIIIERDFPAVFLNITSSSYLFRNDKWKGWEPNISERFNLSEIKPIK | Virulent | non | - |  |
|  | 11 | >WP_012686461.1 peptide ABC transporter substrate-binding protein [Borreliella burgdorferi]  MIIKKRGLLILGIATVISCSAMSKPKDDIVFGVGIGNEPTSLDPQFCSDRLGNLIINELFVGLLRGDPKTGGYRPGLAKN  WDISYDNTCYTFHLRDDIYWSDGIKITASTIRDSYIMALENELDVPHINLLTSKIKNAQEFYETKLNADEVGVKVINEQT  LEITLSRPASYFLDMLTHPIFIPIPTHIVKKFGNKWTSSENMVTSGPFKLKRRILNEEISLEKNEKFYDAKNVAINELIF  VTINDTRRIYSMYEHNEIDAIFNNIPLSLINELILREDFYSADINQIGFFSLNTNVKPLNNVKVREALALAVDRETLTYR  VLDNNFKATRKISPNINNYNYGKKLKPYNPRKAEKLLAEAGYPDGKNFPTLTIKYNKNDLEKEVASFIQSQWKKVLNIDV  EIEPETWVNHISNILKGQYEISVRSWQGDYLDPMAFFNIFETKNSHFASYGYSNPELDELIIKSDLEEDEKLRLEILKKI  EEIIIENDYPIIPIYSVAGNYLFKNDKWLGWNTNNSERFALSEIKPIEE | Virulent | non | - |  |
|  | 12 | >WP_038955294.1 flagellar basal body P-ring protein FlgI [Borreliella burgdorferi]  MNKPMLMLITFATSLLAQTNKASTGLKTDQSFNNSLSESVKLKEIADIYPTNTNFLTGIGIVAGLAGKGDSIKQKDLIIK  ILEKNNIINEIGSNNIESKNIALVNVSLQVKGNTIKGSKHKACVASILDSKDLTNGILLKTNLKNKEGEIIAIASGITQP  NNKLKGSGYTIDSVIINENQNINHSYNIILKKGNYTLINRIHKILTSKKINNKIKSDSTIEIEAKNISLLEEIENIKIET  NPKILIDKKNGIILASENAKIGTFTFSIEKDNQNIFLSKNNKTTIQVNSMKLNEFILKNSNNLSNKELIQIIQAAQKINK  LNGELILEEIDGNQN  Table S3. Docking of multi epitope vaccine with crystallographic structure of TLR 4   \| **parameters** \| **Vaccine** \| **Crystallographic Ligand KDO** \| \| --- \| --- \| --- \| \| **ZDOCK binding energy** \| -1551.49 kcal/mol \| -622 kcal/mol \| \| **Total H-bonds** \| 14 \| 6 \| \| **Total S-S bridges** \| 3 \| 4 \| \| **Total interface residues involved in interaction** \| 27 \| 21 \| \| **No. of non-bonded contacts** \| 149 \| 54 \|   Table S2. Conformational B cells   \| **No.** \| **Residues** \| **Number of residues** \| **Score** \| \| --- \| --- \| --- \| --- \| \| 1 \| A:E28, A:K29, A:A30, A:F31, A:K32, A:K33, A:R34, A:S35, A:V36, A:D37, A:F38, A:S39, A:H40, A:R41, A:K43 \| 15 \| 0.834 \| \| 2 \| A:S1, A:K2, A:K3, A:K4, A:K5, A:E6, A:E150, A:A151, A:A153, A:K154, A:S155, A:K156, A:K157, A:K158, A:K159, A:H160, A:H161 \| 17 \| 0.793 \| \| 3 \| A:I69, A:N70, A:H72, A:D73, A:T74, A:D75, A:K76, A:K79, A:S82, A:T84, A:K85, A:S86, A:S87, A:K97, A:Y99, A:S100, A:D101, A:V102, A:K103, A:P104, A:H105, A:R106, A:V107, A:G108, A:P109, A:G110, A:P111, A:G112, A:E113, A:S114, A:D115, A:D116, A:A117 \| 33 \| 0.633 \| \| 4 \| A:P54, A:T56, A:I58, A:N59, A:S60, A:K61, A:K62, A:K64, A:L65, A:G66, A:Y67, A:S68 \| 12 \| 0.616 \| | Virulent | 0.52 | Allergen |  |
